# Sources of convergence in indigenous languages: Lexical variation in Yucatec Maya

**DOI:** 10.1371/journal.pone.0268448

**Published:** 2022-05-19

**Authors:** Barbara Blaha Pfeiler, Stavros Skopeteas

**Affiliations:** 1 Centro Peninsular en Humanidades y Ciencias Sociales–Universidad Nacional Autónoma de México, Merida, Yucatán, Mexico; 2 Institute of Linguistics, Georg-August University of Göttingen, Göttingen, Lower Saxony, Germany; Leiden University, GERMANY

## Abstract

Linguistic variation in space reflects patterns of social interaction. Gravity models have been successfully used to capture the role of urban centers in the dissemination of innovations in the speech community along with the diffusion of variants in space. Crucially, the effects of the factors of a gravity model (distance and population size) depend on language situation and may result from different sources, in particular processes of vertical and horizontal convergence. In the present study, we investigate lexical variation in contemporary Yucatec Maya, an indigenous language of Mexico, spoken in a situation of generalized bilingualism. This language situation lacks some crucial ingredients of vertical convergence: no variety of Yucatec Maya has the status of a standard variety: the language of administration and education is Spanish (diglossia-with-bilingualism). The present study finds evidence of convergence processes that can be exclusively attributed to horizontal convergence. The lexical distance between speakers decreases in and between urban centers, variants with a large distribution are more likely in areas with a maximum of interactions with other areas. Even Spanish variants are distributed in the sample with a pattern that reveals processes of horizontal convergence: their distribution is accounted for through an areal bias (widespread in areas with a stronger exposition to Spanish) rather by influences from the urban centers (as centers of administration/education) to the rural areas in their surroundings.

## Introduction

### Aims

Dialectal variation in space reflects patterns of social interaction. Since social interactions are more likely between locations that are contingent in space, the amount of interactions is (inversely) correlated with geographical distance. Furthermore, social interactions are determined by human mobility, which takes place in socio-economically conditioned networks of locations, such that inhabitants of a small settlement are more likely to interact with the inhabitants of a relevant urban center than with inhabitants of another small settlement in the same distance. These basic factors are assessed by *gravity models*, in which the amount of social interactions depends on some function of the population size of the locations at issue divided by a function of their geographical distance [1–3: pp. 49–152, 4: pp. 26–29]. Gravity models have been applied to different phenomena in social sciences, economics, epidemiology, and dialectometry, which are generalized in the Eq in (1); adapted from [2: pp. 12]. The estimated amount of interaction *T* between center *i* and center *j* equals the product of a function *f*_α_ of the populations of these centers, *P*_i_ and *P*_j_, divided by a function *f*_β_ of the distance between the centers *i* and *j*. A parameter *k* is used to adjust the overall equation to the rate that applies to the phenomenon at issue. The role of the parameter *k* as well as the exact function of population size (*f*_α_) and geographical distance (*f*_β_) of the centers at issue vary in different systems [2: pp. 10–11, 4: p. 27]. In the following, we discuss their applications to linguistic data.


$$T_{ij}=k\frac{f_{\alpha }(P_{i})f_{\alpha }(P_{j})}{f_{\beta }(d_{ij})}$$ (1)

Gravity models have been applied to the dialectal diversification in geographical space, [5,6], in which case the effect of distance on social interactions is reflected in linguistic variation; see *Fundamental Dialectological Postulate* “Geographically proximate varieties tend to be more similar than distant ones”; [6,7]. In particular, the linguistic distance between two centers is approximated by a *sublinear function* of their distance in space, as found in [5] (see also discussion in [7: p. 154–158, 8]), confirmed for various language situations in [9]–including rural indigenous societies in [10,11]. Furthermore, the effect of geographical distance on linguistic variation is modulated by the ease of social interactions: geographical borders inhibiting interactions are reflected in an increase of divergence, as shown by the distances between Japonic languages in mainland Japan and languages in the island clusters of the Ryukyuan archipelago in [12] or by the increase of dialectal divergence in Norwegian in comparison to Dutch in [13,14]. In general, the function of geographical distance that displays the best fit to linguistic diversification is a logarithmic function of space. The exact effect of geographical distance depends on the ease of social interactions in the geographic region at issue, but it is quite robust in all studies examining the effect of distance.

The function of population size that fits to linguistic data depends on the role of urban centers in the language situation at issue (the concept of ‘language situation’ refers to the conditions of use of a language). Urban centers are gathering places of speakers of various origins, thus enhancing the exchange of linguistic features between varieties. These processes lead to *horizontal convergence*, which arises from the homogenization of dialectal varieties in contact to each other; [15]. Obviously, convergence requires that speakers of different origin interact with each other in their vernaculars, which may not apply to minority languages whose use is limited to closed social networks (e.g., in family contexts) in urban centers. *Vertical convergence* applies to asymmetric situations, in which a variety converges to a different variety that has a privileged status, e.g., a norm, a hegemonic variety, a variety with cultural prestige or even a different language having dominant role in public life; [15–17]. The reflexes of vertical convergence in a gravity model depend on the centers in which the ‘converged-to’ variety is represented. In the typical case of vertical convergence, dialects converge to a standard that is better represented in the administrative and educational institutions of urban centers (‘dialect-to-standard advergence’ in [17]). In such cases, population size correlates with the linguistic distance, reflecting convergence processes in and between urban centers; see British English [18], Dutch [19], Swedish [20], Norwegian [21]. The opposite direction of diffusion applies if linguistic properties that originate in rural areas are adapted from speakers of urban centers reasserting traditional local features [22: p. 385]. These phenomena are also cases of vertical convergence since they involve an asymmetry between a ‘converging’ and a ‘converged-to’ variety; however, the hosts of the privileged variety are the smaller centers in this case. Finally, cases of inverse correlation of population size with linguistic distance between centers are also reported, as for instance for Frisian in the Netherlands, in which case the urban centers are dialectally distinct from their surroundings due to the development of ‘Town Frisian’ varieties; [6,23]. The conclusion from these findings is that convergence is not completely predictable by the amount of social interactions but depends on the socio-cultural evaluation of the centers as well as intentional processes of the speakers (speaker’s attitudes and expressive function of selecting a variety); [24,25: p. 22]. Hence, the exact function of population size in the Eq in (1) when applied to dialectal diversification depends on the exact processes of convergence that take place. In many cases, population size predicts an increase of linguistic similarity between centers (as in the case of dialect levelling), but there are also cases in which population size is inversely correlated with linguistic similarity (as in the case of Frisian).

The rate of the effects of the gravity model (parameter *k* in (1)) can be influenced by various factors. For instance, [181] added the degree of similarity between the varieties at issue as an additional parameter; [26] has shown that the effects of the model in determining linguistic distances depend on the domain of communication (as reflected in different semantic fields).

In the present study, we are interested in the diffusion of dialectal variants in indigenous languages spoken in situations of diglossia-with-bilingualism (i.e., situations in which a second language is part of the repertoire of almost all members of a speech community and is selected in particular types of communication, e.g., in public occasions); [27]. We deal with Yucatec Maya, an indigenous American language spoken in the peninsula of Yucatán (Mexican states of Yucatán, Quintana Roo, and Campeche, as well as some speakers near the northern border of Belize). As in many indigenous communities of Latin America, Spanish is dominant in urban centers, while the indigenous language is still actively used in rural areas. This situation is crucial for the value of urban centers: they may be the hosts of prestige in various respects, but the indigenous cultural and linguistic heritage is rather represented by the remote villages in rural areas where the language is still vividly spoken. Furthermore, there is no established “standard variety”, since writing and use of Yucatec Maya in education are only recent developments, not yet influencing language use. Thus, this language situation lacks some crucial ingredients of the types of vertical convergence that would be reflected in the effect of population size.

The present study examines the dispersion of lexical variation in a data collection from 80 locations in the peninsula. We analyze two classes of lexical variants that have historically different sources: (a) indigenous variants that are expected to reflect patterns of spatial diffusion that arose before the generalization of the bilingualism with Spanish in the last decades, (b) Spanish variants that are used in contemporary communication in Yucatec Maya. Based on methods for aggregating variation, we examine the spatial dispersion of indigenous variants on the peninsula. We first examine the effect of geographical distance, population size and further socio-demographic predictors that are relevant for the situation at issue on the lexical distances between speakers in order to detect the determinants of possible convergence processes. Since the lexical variants in our sample cannot be evaluated with reference to a standard, we examine the properties of their distribution in order to assess the factors that determine the occurrence of variants with a wide distribution in the peninsula. Finally, we examine the distribution of Spanish variants in a separate analysis, since these variants are historically distinct and may reflect different patterns of diffusion in space. In particular, Spanish variants may reveal properties of vertical convergence, diffused from the centers of administration and education (urban centers) towards their surroundings.

### Yucatec Maya

Yucatec Maya is spoken by 796,405 speakers in the peninsula of Yucatán, in the Mexican states of Yucatán, Quintana Roo, and Campeche (census 2010; [28]), as well as by 2,869 speakers in the northern part Belize [29: p. 78]. The central, southern, and eastern parts of the peninsula are covered by tropical forest that until the beginning of the 20^th^ century was only traversed by forest trails. A different situation developed in the northwestern part of the peninsula, which was exploited for the production of henequen (sisal hemp) from the second half of the 19^th^ century; this was the reason for labor migration and the creation of a network of roads and railway lines connecting the larger henequen *fincas*, the capital of Merida, and the ports for shipment [30: p. 801, 820]. Up to the beginning of the 20^th^ century, the major road connections were between Merida and Valladolid in the north, Merida and Campeche in the West, and Merida and Peto in the center of the peninsula [30–32]. The southeastern part of the peninsula (present state of Quintana Roo) was less accessible through road connections in the past and administratively separated from the rest of the peninsula during the Caste War (1847–1933). Currently, the peninsula is part of three federal states of Mexico: Campeche, Yucatán, and Quintana Roo. The state borders do not inhibit mobility and communication, but the creation of administrative centers creates dependencies of the municipalities to the corresponding state capitals.

**Fig 1:**
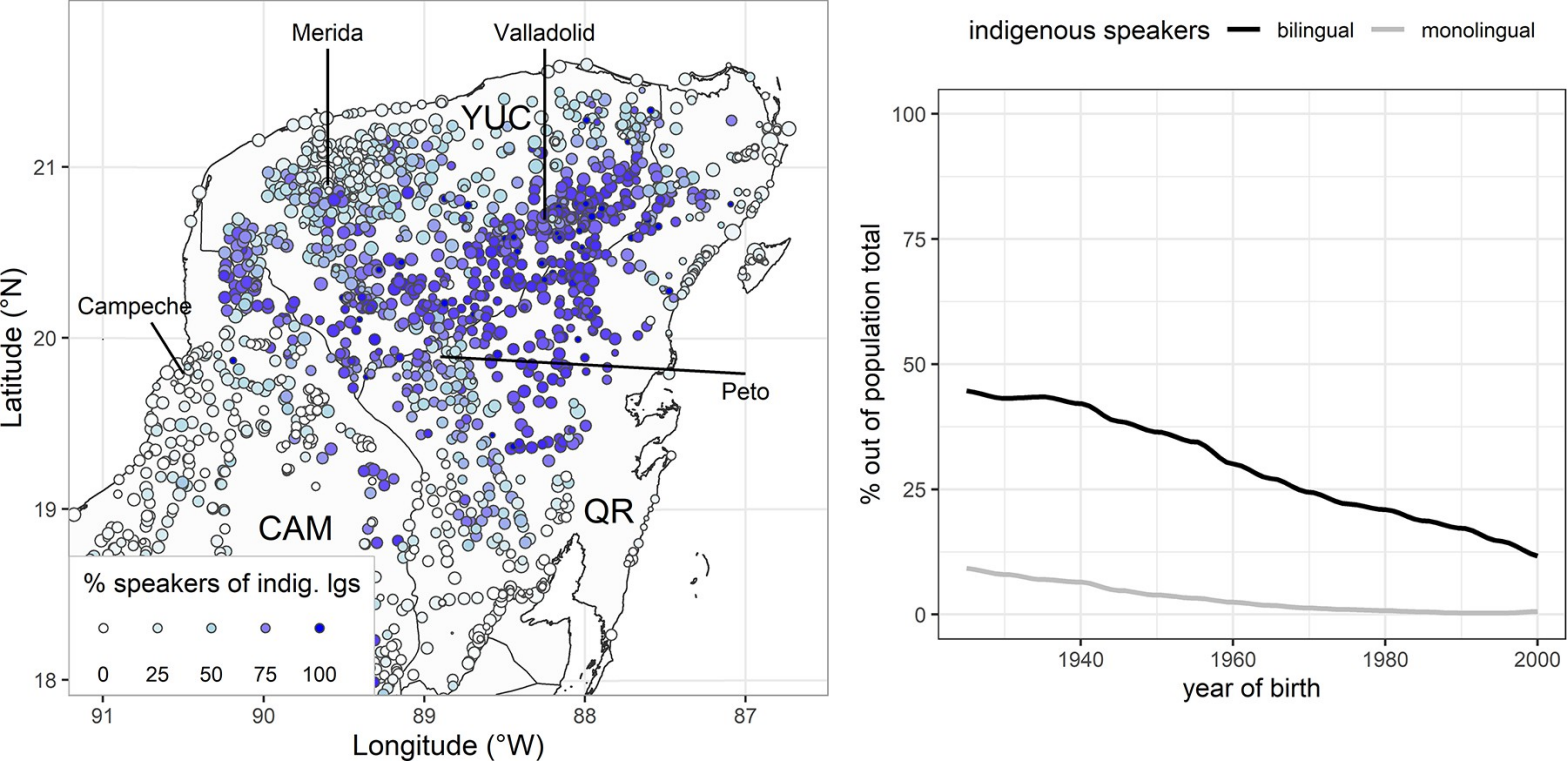
Indigenous speakers in space and time. (A) Diffusion of the indigenous languages’ speakers in space; Proportions of speakers of indigenous languages, plotted in the color scale; Point size: Logarithmized population size of the community (Mexican states: YUC = state of Yucatán; QR = state of Quintana Roo; CAM = state of Campeche). (B) Diffusion of indigenous languages’ speakers in Time. The graphs were created in *R* [36]; *ggplot2* package for plots [37], *ggmap* package for maps [38]; see package versions and further information in **S3 File**. Geographical data were downloaded from the *Database of Global Administrative Areas* (GADM, version 3.6, 2020) at https://gadm.org/.

The indigenous population is laid out in Fig 1A, showing the proportion of speakers of indigenous languages per community based on the 2010 census [27], which is informative for the situation during the period in which the language data used by our analysis were collected (2000–2007). The census data on language use was collected with the question: *¿(**nombre**) habla algún dialecto o lengua indígena*? *Si/No* ‘(name) speaks a dialect or indigenous language?’ (*Cuestionario Básico* of the *Censo de población y vivienda 2010*, section 3, question 8). The Mayan population is prevailing (blue points) in the parts of the peninsula that are covered by tropical forest. The indigenous population shrinks in the touristically exploited coastal areas, in the northwest (the ‘Metropolitan’ area around Merida [33: pp. 208–209]), as well as in the southwestern part of the peninsula (state of Campeche); see [34]. The proportion of Yucatec speakers is higher in rural areas; bilingualism with Spanish is generalized in the population, which is a general trend in the indigenous languages of Latin America [35]. The apparent-time scale in Fig 1B shows the demographic developments in the speaker community, with a radical shrinkage of the population depending on age. The proportion of monolingual speakers born after 1975 is below 1%, which offers a clear picture of the generalized bilingualism (with Spanish) in the indigenous population.

The counts of the census (Fig 1) do not distinguish between different indigenous languages; 94.2% of the indigenous population in the Mexican states of Campeche, Yucatán, and Quintana Roo are speakers of Yucatec Maya [33,39]; other indigenous languages in this area come from recent migrations, e.g., Mayan speakers from Guatemala and Chiapas. The dominance of Yucatec Maya is crucial for the language use in large bilingual centers: the indigenous language is actively used in the communication in several occasions of the everyday life, e.g., in market places, cultural events, etc.

In terms of cultural prestige, the area in the center of the peninsula (Southern State of Yucatán), especially the zone of Peto (Latitude 20.1, Longitude -88.9) is a prominent representative of the indigenous culture, hosting various cultural events and institutions such as the indigenous radio XEPET ‘la voz de los mayas’ [39: p. 312, 40: p. 270]. The main language of school education and public communication in the peninsula is Spanish, which creates a situation of diglossia-with-bilingualism; see [41]. This type of language situation is relevant for understanding the current developments, since the role of an institutionalized variety is undertaken by a different language. The representation of the indigenous language in public life changes in the recent years, with various measures towards the institutionalization of the language, e.g., the establishment of the ‘intercultural bilingual education’ program in the peninsula [42: pp. 23–26] and concomitant attempts for standardization, e.g., publishing a norm for writing in 2014 [43]; see summary and evaluation of current developments in [44,45]. However, at least in the time of the data collection examined in this article (2000–2007), the majority of the indigenous population was not reached by these initiatives, such that the incipient standardization efforts could not yet have a serious impact on language use; see also [46: p. 386] on Mayan languages in general. All participants of the present data collection were native speakers Yucatec Maya and no one had learned Maya at school.

Reflexes of the generalized bilingualism are found in various linguistic layers, from the frequency of Spanish loanwords [33,47–49] to grammatical phenomena such as the emergence of definite articles [50], the generalized use of plural marking [51], or the general use of the SVO order in neutral contexts [52]. Mayan people distinguish between the vernacular language (*xe’ek’ maaya*, lit. ‘mixed maya’) and the high-prestige authentic use of language (*jach maaya*, ‘pure Maya’, lit. ‘very Maya’) [53: §4, 54: pp. 490–491, 55: p. 38, 56]. The *jach maaya* is not a discrete variety of the language (i.e., having a certain lexicon and grammatical rules), but rather the construal of authentic usage of Yucatec Maya, as part of the folk ideology [55: p. 39]. Mayan people ascribe the use of *jach maaya* to older speakers in remote areas in the tropical forest or to the carriers of cultural heritage, e.g., to the followers of the cult of the speaking cross (*cruzo’ob*) of Quintana Roo [34: p. 372] or to other carriers of prestige such as the broadcast journalists of ‘Radio Peto’. Within the recent developments of the institutionalization of the language, individuals (e.g., teachers or intellectuals) may attempt to speak *jach maaya* by avoiding words of Spanish origin or selecting various forms of hypercorrection (e.g., avoiding phonological processes such as contraction, which reduce the transparency of morphological structures) or by selecting particular words that are considered to be ‘authentic’ because of their origin in historical sources [44]. Crucially, *jach maaya* is not a standard variety that could trigger homogenization processes, but rather a folk construal motivated by purist ideologies.

Dialectal variation was already mentioned in the sixteenth century in the *Motul Dictionary* [57]. Recent dictionaries report lexical variants in various regions [58–60]. Beyond lexicon, dialectal variation is reported for various areas of grammar, such as segmental phonology [54], tonal realization [34: p. 375], morphonological processes [61], choice of auxiliaries [55], and phraseologisms [55]. Nevertheless, all sources agree that local differences are rather limited and do not severely restrict the mutual intelligibility between speakers of different regions [53: §^4^, ^60^: p. ix, 62: p. 14].

Some studies report that the major axis of dialectal differentiation is between the western and the eastern part of the peninsula [62: p. 14, 63: p. 2]. More fine-grained distinctions have been proposed in lexicological studies, defining areas by means of the occurrence of dialectal variants. Five such areas were defined by [59]: (a) the Agave Area around Merida in the north, (b) the zone of Camino Real in the West, (c) the Eastern part of the peninsula (d) the Center of the peninsula, which contains the southern part of the state of Yucatán and center of Quintana Roo, and (e) the region Los Chenes in Campeche. A similar classification is also proposed by [34] with two differences: (a) Campeche is a single area containing Camino Real and Los Chenes; (b) the Center is divided in two separate areas: the southern part of the State of Yucatán and the center of Quintana Roo. A further area that is not captured by these classifications is the ‘Metropolitan’ area around Merida, which is characterized by the lower density of the indigenous population [33: pp. 208–209]; see northwestern part of Fig 1A.

[55] investigate the distribution of lexical and grammatical variables in the peninsula and present examples of lexical variation illustrating various patterns of distribution in geographical space. While the contrast on the East-West axis is the most frequent pattern (e.g., *k’aax* ‘grass’ in the eastern part of the peninsula vs. *xíiw* ‘grass’ elsewhere), some variables involve a contrast in the north-south axis (e.g., *hanal* ‘food’ in the north vs. *o’och* ‘food’ in the south). In line with the view in current dialectological studies [64], [55] demonstrates that the diffusion of variants in Yucatán cannot be assessed by a clear-cut distinction between dialects defined by bundles of isoglosses, since dialectal variants are dispersed in contiguous spatial regions in very different patterns.

Some aspects of the reported variation in space are intertwined with sociolectal variation. [53: p. xii] reported two varieties in the time of data collection (1930–1933), dubbed as ‘type A’ and ‘type B’. Type A was a conservative variety that was better represented in the southern part of the peninsula, i.e., Quintana Roo, in the eastern part of Campeche and the Corozal district of Belize. Type B was prevalent in the northern part of the peninsula, especially in the state of Yucatán, as well as in the remaining places of Campeche. In some places (city of Campeche and some locations in Camino Real), Type A was the variety of the old generation and Type B the variety of the young generation. Type B was characterized by several instances of fusion and deletion at the phonological level as well as by morphological simplifications.

In sum, Yucatec Maya is spoken in a large area without inhibiting geographical borders, which predicts a gradient diffusion of dialectal variants in space. The division of the peninsula in three administrative areas (federal states of Campeche, Yucatán, Quintana Roo) creates new dependencies between municipalities and the administrative centers, but the state borders do not inhibit communication between communities. With respect to mobility axes, there is a key role of the Metropolitan area (around Merida, the capital of Yucatán), which offers the main connections to Campeche in the South, to Valladolid in the East and to Peto in the center of the peninsula. Bilingualism with Spanish is generalized in the indigenous population, monolingual speakers being below 1% in the population born after 1975. The indigenous population is predominant in rural areas in the central/eastern parts of the peninsula. Dialectal variation is present at different linguistic layers (lexicon and grammar) without inhibiting intelligibility between speakers of remote locations. At least at the period of data collection examined in the present study (2000–2007), standardization initiatives were at an incipient stage without affecting the communication practices of the most part of the population.

## Materials and methods

### Data collection

The present study is based on a Yucatec Mayan dialectological data resource created at the *Universidad Autónoma de Yucatán*, Merida, México (data collected between 2000 and 2007; funded by the CONACyT 36387-H); [65]. The data was collected with Spanish prompts (orally presented by an instructor); see similar procedure in [66]. The participants were instructed to translate the Spanish prompts into Yucatec Maya in the way the language is used in their place of living. The prompts were minimal in order to avoid additional sources of variation (e.g., influence of the phonological environment): referential expressions were elicited with single words, e.g., *achiote* ‘annato shrub’, *gavilán* ‘hawk’; modifiers were elicited with simple phrases, e.g., *algo lo* *más* *corto*, *como un palo* ‘something that is the shortest, like a stick’ to elicit the intensifier; actions and modifiers of actions, were elicited through simple clauses, e.g. *lo hizo* *rápido* ‘he did it fast’ for eliciting the expression of ‘fast’, *empecé a conversar* ‘I started to talk’ for eliciting the expression of inchoative aspect. The questionnaire contained 665 entries arranged in thematic units, offering an encompassing resource for the study of various linguistic components: lexicon, phonology, morphonology, morphology, syntax.

Using prompts in Spanish circumvents the potential dialectal bias that could apply if the elicitation would be carried out in Yucatec Maya. However, translation is associated with some artefacts that must be taken into account during the interpretation of the data. A major drawback is the bias of the source language, which might foster the use of elements or structures of the language of instruction [67: p. 123, 68: p. 43, 69: p. 78]. At the level of lexical choice, which is the object of the present study, speakers tend to maximize the use of words of Mayan origin since the task is to translate the Spanish prompts into Yucatec Maya (although they were not instructed to avoid Spanish variants); see report in the Subsection ‘Spanish variants’ below. An artefact of the elicitation procedure relates to the accuracy of the translation. The speakers may use related concepts that do not exactly lexicalize the concept presented in the prompt. Hence, the faithfulness of the obtained data must be validated based on expert knowledge, to verify that the elements elicited with the same prompt correspond to the same target concept; see Subsection ‘Lexical variation’ below.

### Speaker sample

Speakers participated in the data collection on voluntary basis. They were informed that the recordings will be used for scientific purposes (documentation/description of Yucatec Maya), that their participation is voluntary, and that their consent can be withdrawn in the future. Given that the majority of the speakers were not literate, informed consents were obtained orally; see [70: p. 39] for practices in non-literate language communities. The participants gave their permission to the instructor to record the session; audio recordings took place overtly with a digital audio tape recorder that was placed in a position clearly visible to the participant. They were remunerated according to the local standards. The data were anonymized in the data resource and in all analyses; the data collection does not contain sensitive data (e.g., about the socio-economic status of the participants) that may be used to harm the individuals who contributed to the present resource.

The corpus contains 157 speakers (53 female, 104 male) from 80 locations; see **S2 File/locations**, and visualization of the sample in Subsection ‘Predictions’. The sample contains representative locations within the area hosting indigenous population, especially including the regions mentioned with respect to dialectal diversification: Agave area, Center, Northeastern area, Los Chenes, Camino Real, Metropolitan area, Belize. Within each location, the researchers who conducted the data collection sought for maximally competent speakers who were raised in the respective location (born or living at least during the last 30 years in the location at issue, with parents originating in the same region). The number of speakers per location was not strictly controlled: 76 locations were represented by 1–4 speakers, while further 4 locations were represented by 5, 6, 7, and 10 speakers respectively (**S2 File/speakers**). The year of birth of the participants was spread out in the range 1906–1989 (age range at the time of the data collection: 18–97); see properties of the distribution under ‘Explanatory variables’ below.

### Data classification

The scope of the present study is restricted to lexical variation; see [71] for a previous study on this type of data. After a qualitative inspection of the entire corpus, we identified 52 variables displaying lexical variation between elements of indigenous origin, which includes (a) cases of different stems with the same meaning and (b) cases with lexically-conditioned sound alternation. The data was classified by expert knowledge, relying on the available lexicographical sources as well as the research on the phonology of Yucatec Maya; see [66] on the comparison between expert knowledge and edit distances.

The goal of the classification is to identify lexical variation for the expression of the same concept. For instance, for the target concept ‘annato shrub’ (Spanish prompt: *achiote*, bixa orellana) speakers used the synonymous variants *k’uxub’* and *kiwi’*, which are already attested in Colonial Yucatec Maya [58,59]. The obtained data were excluded if speakers used a different concept, e.g., some speakers (*n* = 4) used *xa’ak’* ‘annato paste’ for the same concept. An instance of lexically-conditioned sound alternation are the variants *kaláant* and *kanáant* for the concept ‘protect’. This alternation is not predictable by phonological rules that generally apply in certain phonological environments. Reflexes of phonological rules, such as the debuccalization of syllabic codas (e.g., *jun-p’éel* ~ *jun-p’éej* ‘one-classifier.inanimate’) or the alternation of final nasals (*sakan ~ sakam* ‘dough’), which are pervasive in Yucatec Maya (see [61]), were not considered in the present study.

Some concepts appear in more than one prompts of the questionnaire, e.g., the concept ‘dough’ is elicited with the prompt *masa* ‘dough’ and with the prompt *una bola de masa* ‘a ball of dough’. In these cases, we only used the entry with the maximal number of valid tokens (in order to avoid biases from concepts that are overrepresented in the sample).

Indigenous variants and variants of Spanish origin are historically different and their diffusion in the population may be influenced by different factors. For instance, while the exposure to Spanish is a reasonable predictor for the occurrence of Spanish variants, it does not straightforwardly influence the choice among indigenous roots. Hence, the choice between indigenous variants and the choice between Mayan or Spanish variants were examined in two separate analyses (see Subsections on ‘Indigenous variants’ and ‘Spanish variants’). With Spanish ‘variants’ we refer to variants of Spanish origin in the collected data, which may include loans that are integrated to the Yucatec Mayan lexicon, but also instances of code-switching that are often used in spontaneous discourse as well as during data elicitation.

### Data analysis

All steps of data processing, visualization and statistical analysis were performed in R [36]; see R-script in **S3 File**. Variation between speakers was assessed by calculating the dissimilarity of their responses and visualizing their (lexical) distances in a color continuum, as in [72]. Statistic modelling was used to assess preferences in the choice between indigenous variants as well as the choice of indigenous vs. Spanish variants. The procedures used for data analysis are introduced in the following.

#### Lexical distance between speakers

The variants were dummy coded (0 = absence, 1 = presence of a certain variant) for each concept separately. Internal consistency of the data set was assessed with Cronbach’s alpha ([73]), which ranges between 0 and 1 –with higher values indicating high reliability, minimum level of adequate reliability: 0.7; [74]; see applications in dialectometry in [72: pp. 170–175; 75, 76]. A distance matrix was computed by measuring the dissimilarity between speakers based on their lexical choices, dealt with as asymmetric binary variables [77: pp. 858–859]. The dissimilarity index *D*_αβ_ between two speakers *α* and *β* is defined by the number of items in which both speakers have a different value (*d*_*αβk*_ = 1 if the values of speakers *α* and *β* in the *k*^th^ item are different or *d*_*αβk*_ = 0 if not), divided by the total number of items in which the value is present in either speaker (*δ*_*αβk*_ = 1 if the value is present either in *α* and/or in *β*; *δ*_*αβk*_ = 0 otherwise). The dissimilarity index ranges between 0 (= all values of speakers *α* and *β* are identical) to 1 (= all values are different). Values that were classified as ‘non-valid’ were excluded from this analysis.

#### Multidimensional scaling

The distances of the dissimilarity matrix (distances of each of the 157 speakers to the other 156 speakers) were reduced to three variables by *Classical Multidimensional Scaling* [72,78]. The proportion of variance of the original data that is captured by this analysis was assessed by calculating the correlation between the original dissimilarity matrix and the distance matrix resulting from the dimensions of the multidimensional scaling. The squared correlation coefficient (*R*^2^) ranges between 0 (= a zero part of the variance in the original data is captured by the analysis) and 1 (= the entire variance in the original data is captured by the analysis). The three dimensions of the multidimensional scaling were finally mapped onto the three dimensions of RGB colors, offering a visualization of the lexical distance between speakers or locations in the color continuum, and were plotted in geographical space [20,72,79–81].

#### Determinants of lexical distance between speakers

A linear model was fitted to the coefficients of dissimilarity (reflecting the lexical distance between speakers) in order to assess its determinants. The dependent variable of this model is the dissimilarity between each pair of speakers in the sample. Starting with the gravity model, the explanatory variables were the Geographical Distance and the Population Size of the locations at issue (see details about the computation under ‘explanatory variables’). Furthermore, we included two factors that may account for a part of the variation in the data, the proportion of the Indigenous population at the locations at issue, which is informative for the chance of using Maya in a certain location, and the apparent time scale of the speakers’ year of birth (Time). Since this analysis assesses the dissimilarity of pairs of speakers, the variables for Population Size, Indigenous Population, and Time contained the product of the values of both speakers (Time) or both corresponding locations (Population Size, Indigenous Population). All explanatory variables were rescaled to a [0–1] interval, such that the magnitudes of their effects are comparable.

#### Choice between indigenous variants

If homogenization of dialects proceeds through the spread of a standard variety, dialect levelling can be efficiently estimated by the differences of the local varieties to the ‘standard’; see [19,82] on Dutch, [71] on Italian, [83] on Japanese. In the absence of a standard variety, as in Yucatec Maya, this procedure cannot be applied. As discussed in the section on ‘Yucatec Maya’, the construal of *jach maaya* ‘pure Maya’ is not a discrete variety with certain vocabulary items, but a cross-dialectal idealization of authentic usage of the indigenous language. Furthermore, the available lexicographical knowledge and the resources for further Yucatecan languages do not allow an assessment of the variants based on their availability in earlier diachronic stages or in the proto-language of the Yucatecan language branch.

**Fig 2:**
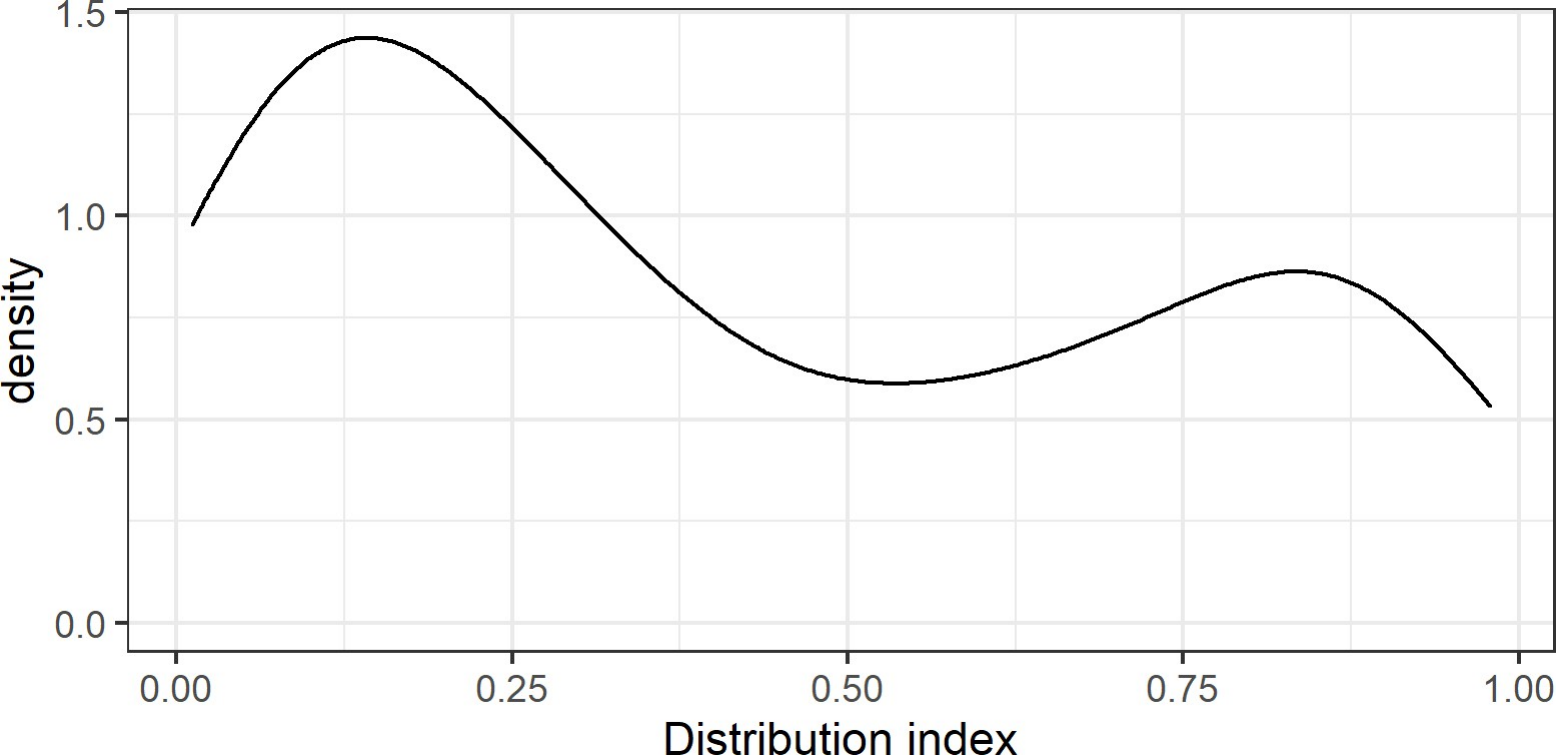
Distribution index of the variants (density). Density of the distribution indices of the indigenous variants in the collected data (mean = .42; standard deviation of the mean = .32).

Convergence processes can only be assessed by the distribution of the variants in the data collection. Some indigenous variants are widespread, while other variants only rarely occur in the sample. For instance, the prompt *calabazo* ‘squash’ was rendered as *chúuj* by 135 speakers and as *k’úum* by 13 speakers. This difference is captured by the relative frequency of the variant (variant frequency divided by the *n* of valid data of the corresponding variable), which we dub Distribution Index: DI(*chúuj*) = 135/148 = .91; DI(*k’úum*) = 13/148 = .09). The density of the distribution indices of the indigenous variants in our sample is presented in Fig 2. Variants with wide distribution in the population have high relative frequency (values close to 1 in Fig 2), while variants with limited distribution have low values (close to 0).

The Distribution Index is informative for convergence processes. For instance, if homogenization processes are at issue in urban centers, variants with wider distribution should be more frequent in places with larger population size; if homogenization increases during time, variants with wider distribution should be more often used by younger generations. On the other side, variants with limited distribution are more likely to index social meaning: speakers who want to assert local accents may select variants that are only locally distributed and are less widespread in the peninsula.

#### Generalized additive mixed-effects models

In order to assess the factors that determine the distribution of dialectal variants in geographical space we used *generalized additive mixed-effects models*, which assess non-linear predictors by means of thin plate regression splines; see [84]; see modelling linguistic data in [85], applications in dialectometry in [19,71,86–88]; computed with the package ‘mgcv’ in R [89]. These models were used (a) in order to assess the determinants of the distribution index (i.e., the choice of variants with wide or limited distribution in the peninsula) and (b) in order to assess the determinants of the choice of Spanish or Mayan words. Both models have the same fixed-effects structure.

Geographical coordinates were introduced to the model as a smooth term, which captures the non-linear effect of space on the dependent variable, while the fixed factors Population Size, Indigenous Population, and Time were modelled as linear predictors. Furthermore, these analyses contain the random effect of Concept, relating to the different lexicalizations/target words examined in the corresponding analysis.

In all calculations, we applied a stepwise procedure of forward model selection for the fixed factors, see [19]. The random-effects structure was the maximal structure that converges in a model without fixed effects, which is kept constant in all model comparisons. Model selection was based on the model fit by means of Log-Likelihood tests. Models were fit with ordinary maximal likelihood, as recommended for model comparisons with the same random structure [90, pp. 174–175, 91, p. 122]. Model comparisons for generalized additive models were computed with the package ‘itsadug’ in R [92].

#### Explanatory variables

**Fig 3:**
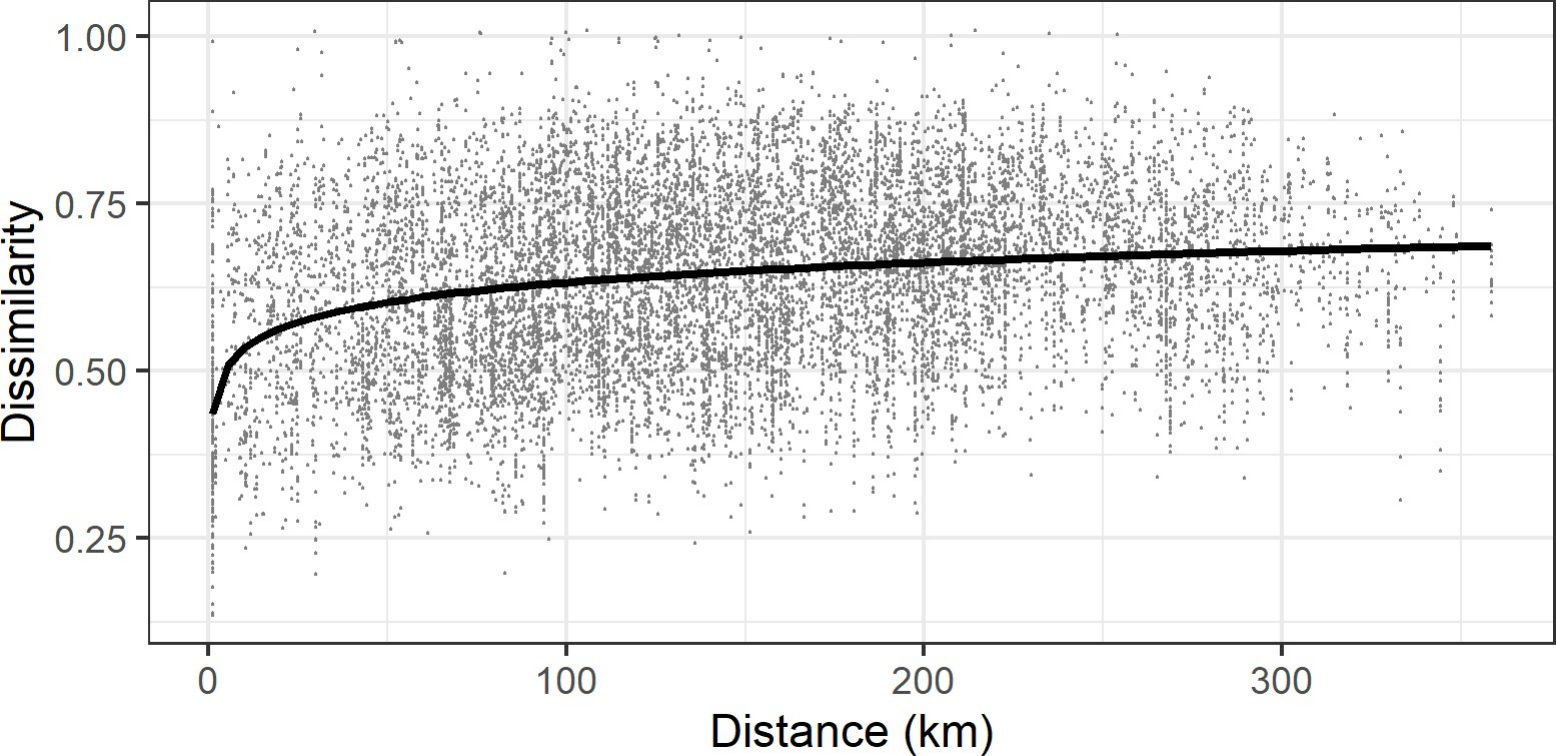
Geographical distance and dissimilarity between speakers (central tendency by logarithmic curve).

In the modelling of lexical distance between speakers, Geographical Distance was computed as the surface distance (in km) between the geographical coordinates of speakers’ locations; see Fig 3. It has been shown that linguistic distances are better captured by a sublinear function of the geographical distances [5–11]. In modeling dialectal variation, geographical distances are often logarithmized; see, e.g., [1,19]. In order to test whether logarithmization increases the goodness of fit to our data, we compared two linear models on Dissimilarity with plain or logarithmized Geographical Distance as predictor. The plain Geographical Distance has a significant effect on Dissimilarity (slope: .0004, *t* = 28.1, *p* < .001), while the logarithmized Geographical Distance reaches a higher estimate (slope: .03, *t* = 33.1, *p* < .001). The two models differ in goodness of fit, as assessed by *R*^2^ (proportion of variance explained by the model), which has a value of .06 for non-logarithmized kilometric distances and .08 for logarithmized kilometric distances. We conclude that logarithmized distances have a better fit to this data, as shown also by earlier studies on the impact of geographical distance on linguistic similarity.

**Fig 4:**
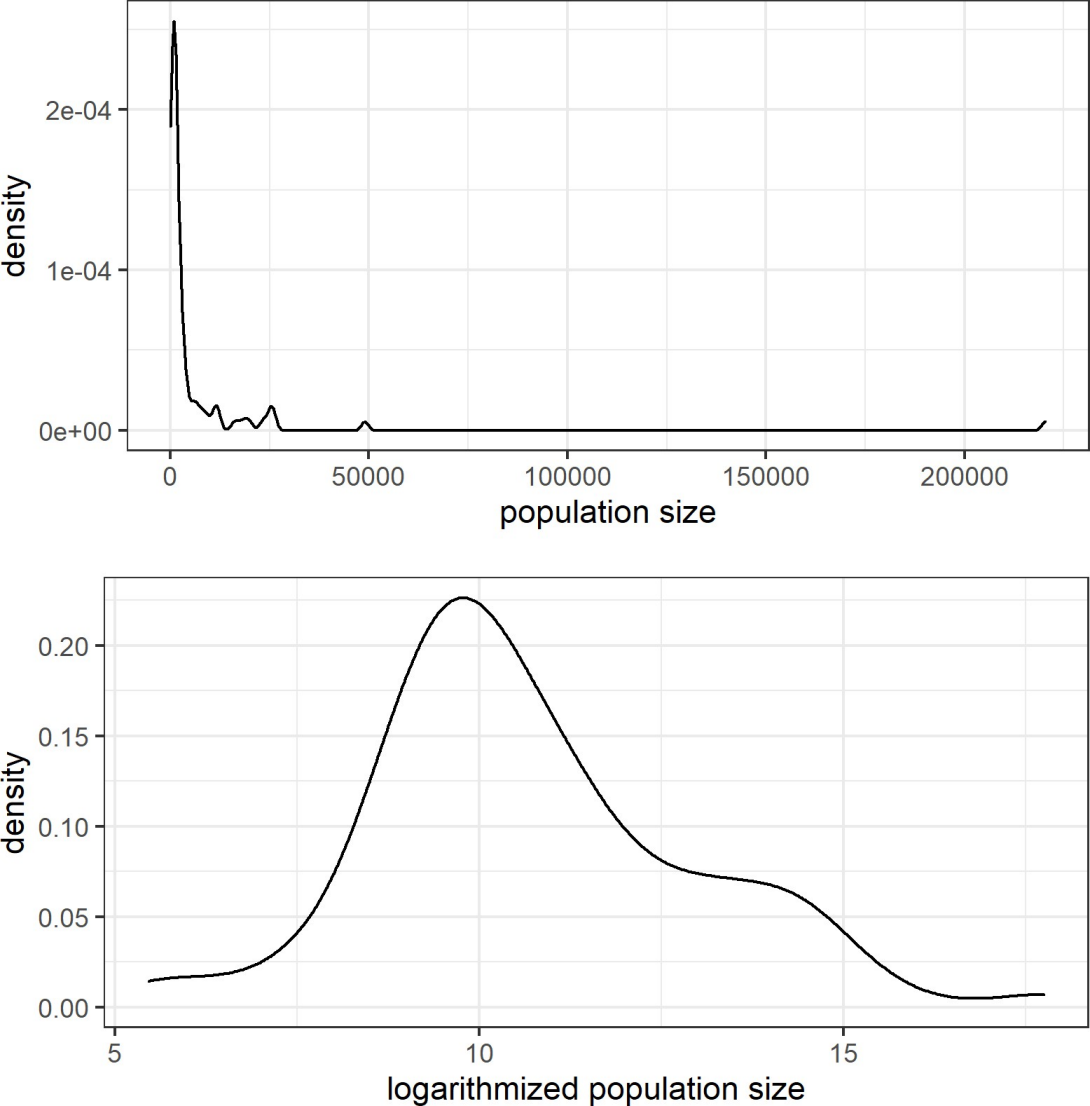
Population size (density). (A) Density of the population size of the locations in our sample. (B) logarithmized population size of the locations in our sample.

Population size is known to follow a power-law distribution and is therefore logarithmized in various studies [1,6,19,71]. The population size of the locations in our sample shows exactly this type of distribution, as shown in Fig 4A, with two very large centers (City of Campeche 220,389 inhabitants; Valladolid: 48,973 inhabitants), ten locations with more than 10,000 inhabitants and further 68 locations with less than 10,000 inhabitants. In order to avoid the outlier effects of the few large locations in our sample, we logarithmized our data (base: 2), which rendered the distribution of population size displayed in Fig 4B.

Beyond Geographical Distance and Population Size, we added two further factors in our models that are independent of the determinants of the gravity model: the birthyear of the speaker (Time) and the proportion of Indigenous Population in the location at issue. Time captures the reflexes of language change on the apparent time scale of the birthyears of the speakers in the examined sample (1906–1989). The birthyears were spread out with a symmetrical distribution, such that the mean is very close to the median ($\bar{x}$ = 1953.3, $\tilde{x}$ = 1953); see full listing in **S2 File/speakers**.

The proportion of Mayan speakers in the center at issue (Indigenous Population) is informative for effects of the decrease of Mayan speakers in certain areas of the peninsula. We considered the proportion of indigenous population (instead of the absolute count of indigenous speakers), assuming that the proportion of indigenous speakers in a community is a better predictor of the chance of social interactions in the indigenous language. For instance, the city of Campeche counts 8,598 Mayan speakers (4% out of 220,389 inhabitants) and the city of Nunkiní 4,532 Mayan speakers (77% out of 5,859 inhabitants). Using the proportions as predictors corresponds to the intuition that the chance of using Yucatec Maya in everyday life is higher in Nunkiní than in Campeche and reflects the basic assumption that convergence processes result from social interactions in the language at issue.

Correlation tests between the explanatory variables were used to determine whether these variables are independent from each other; by convention, a correlation is high if the value of Pearson’s coefficient is above |.7|; see [93]. A correlation test between the (logarithmized) Population Size and the proportion of Indigenous Population does not reveal a high correlation (*r* = –.52). The negative correlation reflects the fact that the proportion of Indigenous Population decreases in urban centers. The correlation is not high, since the proportions of Indigenous Population do not only depend on the difference between rural and urban communities, but also vary between the geographical areas of the peninsula (see Fig 1A). Time is weakly correlated with Indigenous Population (*r* = .05) and Population Size (*r* = .1) in this sample. Correlations between the fixed effects were also tested for each model separately; see reports in **S3 File**: the correlation coefficients did not reach the |.7| threshold in the examined models.

## Predictions

**Fig 5:**
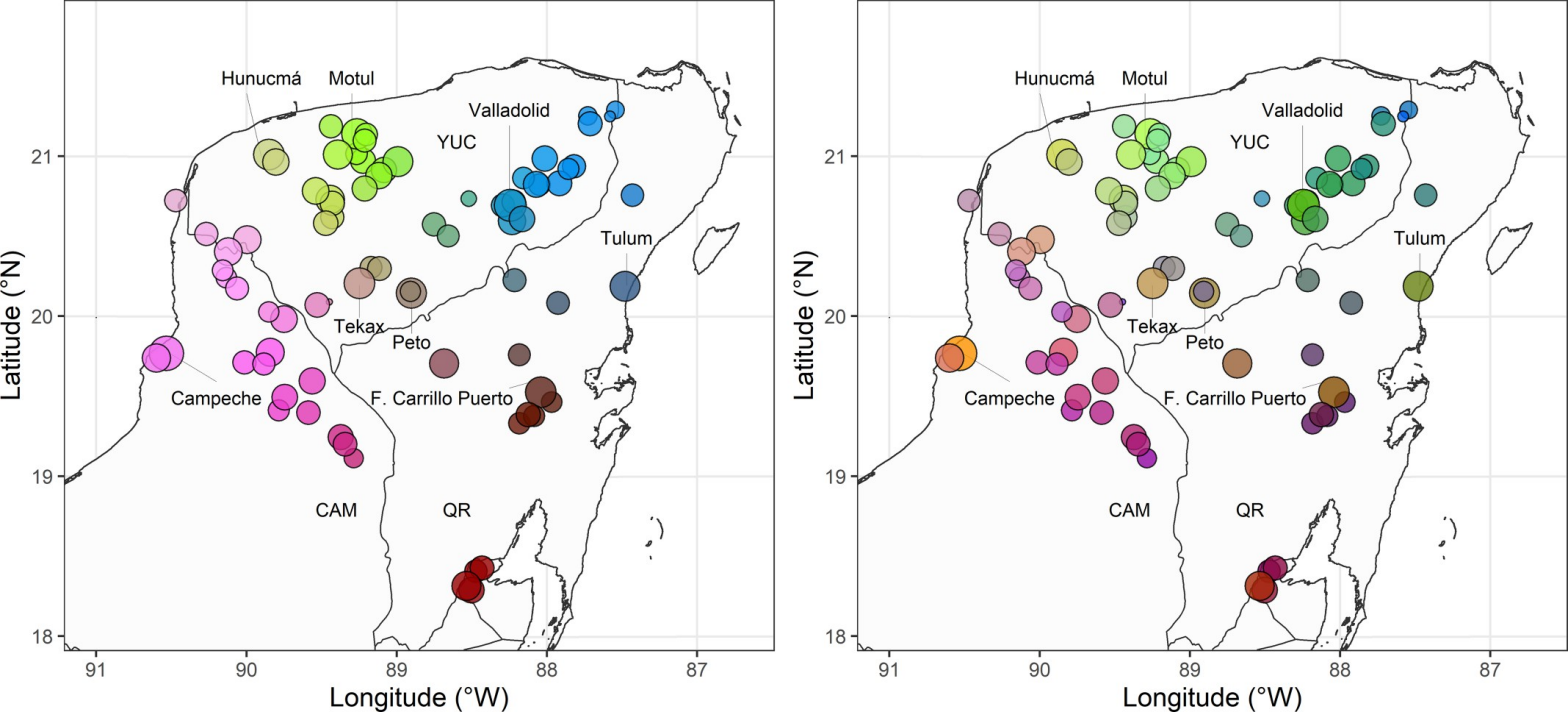
Model predictions. Predicted similarities between locations represented by RGB-Color values. Dot size: Logarithmized population size (the eight largest cities of the sample are labeled). Left Panel: Predictions of the *Wave Theory* (distance matrix containing the logarithmized distances between locations). Right Panel: Predictions of the *Gravity Model* (distance matrix containing the logarithmized distances between locations, divided by a distance matrix containing the product of logarithmized population sizes).

Given the amount of previous knowledge in dialectology about the diffusion of linguistic variants in space, we take for granted that geographical distance will influence the (dis)similarity between speakers of various locations. The factors of interest are the social variables that will be informative for the language situation at issue. With this background, the null hypothesis is a model only based on the *Fundamental Dialectological Postulate* in [6], according to which dialect variation is only determined by geographical distance. This prediction corresponds to the *Wave Theory* of language change, according to which events of change evolve in certain areas and radiate outwards in geographical space ([8,94,95: p. 721]): if the rate of diffusion of linguistic variants in geographical distance is constant, the variation will render a dialectal continuum in which variation is equally distributed in space. This prediction is illustrated for the sample locations in Fig 5A, which is based on a distance matrix with the logarithmized kilometric distances of the sample locations to each other, reduced to three dimensions by Classical Multidimensional Scaling and visualized in RGB colors (see procedure in ‘multidimensional scaling’). The outcome of the multidimensional scaling is sufficiently correlated to the original data (*R*^2^ = .74). The distance between color values represents the predicted linguistic difference between locations if the rate of diffusion of variants in space is constant. The gravity model predicts that the linguistic distances between locations are inversely correlated with the product of their population sizes, since linguistic distances are inversely correlated to the frequency of social interactions predicted by the model in (1). In order to visualize this prediction, we divided the geographical distances by the product of the logarithmized population sizes (in thousands) of each pair of locations. Both variables were rescaled to a [.001,.999] interval, population size products were additionally centered around 1. The resulting distance matrix was reduced to three dimensions by multi-dimensional scaling (*R*^2^ = .65), whose outcome is visualized in Fig 5B. The effect of population size is maximally discernible with large centers, e.g., in the city of Campeche or in Felipe Carrillo Puerto, whose color values clearly differ from their surroundings.

As discussed in the section ‘Aims’, the effects of population size may come from different sources, in particular processes of horizontal and vertical convergence that take place in and between urban centers. In the absence of a standard variety, vertical convergence to a privileged variety of the same language is not applicable. In a situation of diglossia-with-bilingualism, such that a different language has the role that is otherwise fulfilled by the standard variety, vertical convergence can only be reflected in effects of language contact, e.g., in loanwords from a contact language used in public communication.

Horizontal convergence may lead to homogenization processes in and between urban centers, which predicts an effect of Population Size on the increase of similarity between speakers (decrease of lexical distances in/between urban centers). Further reflexes of horizontal convergence may apply to the distribution of variants in the peninsula: variants with a wider distribution are more likely to appear in the nodes of mobility, i.e., in the centers that connect the different areas of the peninsula. This applies to the area of Merida that connects the main centers of Valladolid in the East, Campeche in the Southwest and Peto in the center of the peninsula (see Fig 1A).

The proportion of Indigenous Population in the communities at issue (Fig 1A) is the result of recent migration processes, which mainly took place within the 20^th^ century. Hence, we expect to find an effect of this factor in the recent strata of the vocabulary, especially in the occurrence of Spanish variants, which should be more likely in locations that offer fewer opportunities to use the language in everyday life. This is the case in large urban centers and in areas with lower density of Yucatec Mayan speakers.

The native speakers of the present sample represent a wide range of generations (year of birth: 1906–1989), which may be informative of changes in time; Fig 1B. Language changes over time by innovations that take place during transgenerational transmission: in the absence of exchange, the null hypothesis is that divergence increases over Time, giving rise to diversification between dialects or languages [96, p. 528]. Alternatively, an increase of similarity over time provides evidence that homogenization processes are in progress, as shown in cases of dialect levelling, e.g., in British English [18], Dutch [19], Swedish [20], Norwegian [21]. In the particular situation of Yucatec Maya, we have seen that the construal of *jach maaya* is part of the folk ideology and may play a role in the speech production of young educated speakers in the peninsula. Since this construal is not a discrete variety with a fixed vocabulary but rather a desire to revitalize the ‘authentic usage of language’ as it is still thought to be preserved in remote areas, it may reinforce the use of variants flagging local accents; see similar phenomena in [22].

## Results

### Spatial distribution

The total sample contains 52 (variables) × 157 (speakers) = 8,164 data points. Missing values (*n* = 1,689, 20.7%), i.e., cases in which the native speakers did not provide any data or lexicalized a different target concept (see “Lexical variation”) were excluded from the analysis. The valid dataset (*n* = 6,475, 79.3% out of total) contains variants of Mayan (*n* = 6,304, 96.4% out of valid) and Spanish origin (*n* = 171, 2.6% out of valid); see full listing in Supporting Information (**S2 File/variants**).

**Fig 6:**
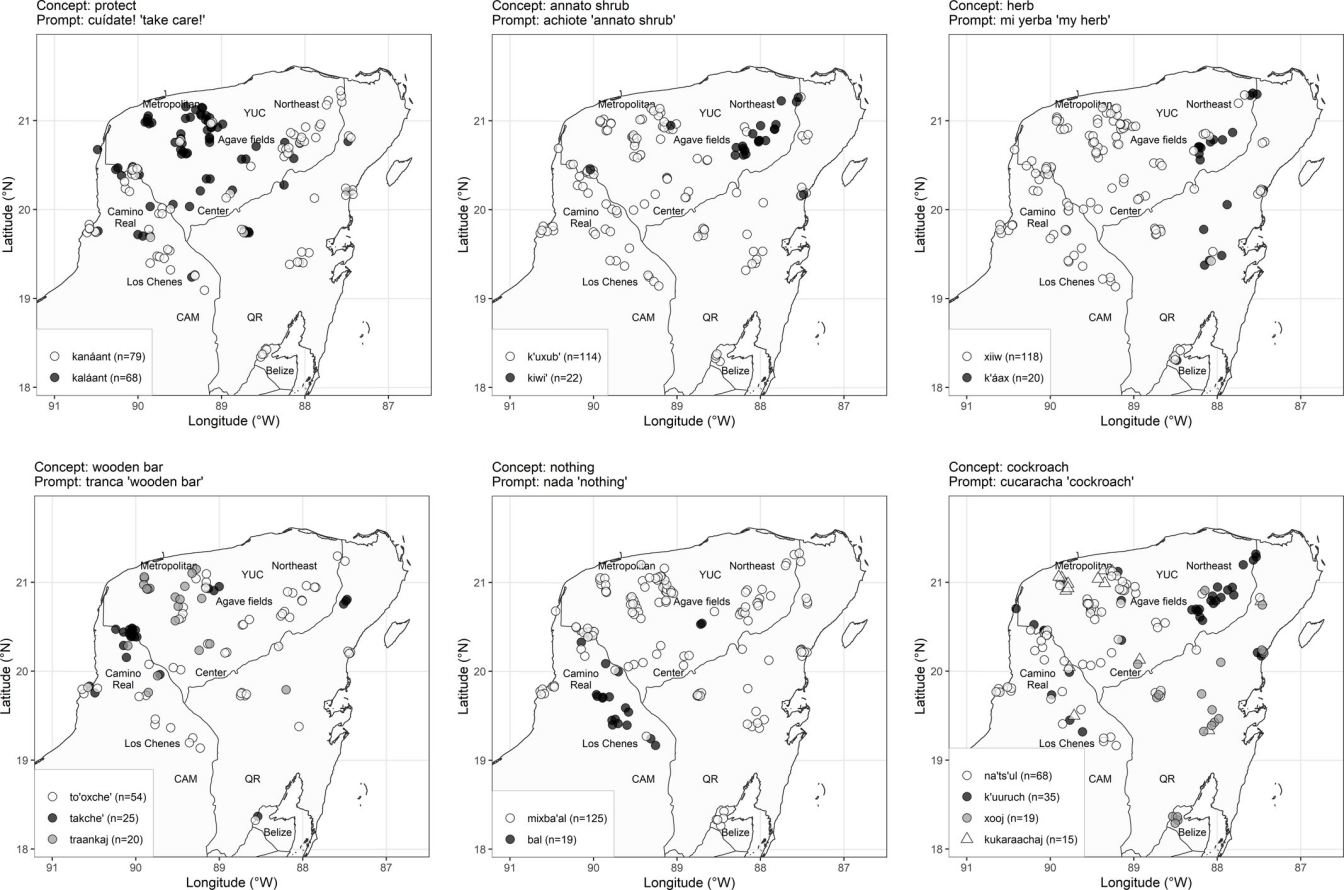
Variants in geographical space.

Fig 6 illustrates various patterns of distribution of the obtained variants in space (see figures of all target concepts in **S1 File**). The target concept is given in the title of each graph, followed by the exact prompt as used in the elicitation sessions. The distribution of the variants in space often suggests an areal bias. The target concept ‘protect’ is rendered with the stem *kaláan* in the central part of the state of Yucatán, while the preferred option otherwise is *kanáan* [59: p. 90]. Some variants are characteristic of the northeastern part of the peninsula. The variants *k’uxub’* and *kiwi’* ‘annato’ (Spanish *achiote*, bixa orellana) are synonyms, both forms being already attested in Colonial Yucatec Maya [58: pp. 322/427, 59: pp. 96/122]. The form *kiwi’* is dominant in the northeast, *k’uxub’* elsewhere. Some variables are dispersed in the northeast and in Quintana Roo; see *k’áax* for ‘herb’ vs. *xiiw* elsewhere, supporting thus the idea of an eastern variety [55,63]. Some variables are characteristic of Camino Real, e.g., *takche’* ‘wooden bar’ vs. *tóoxche’* elsewhere [55: p. 35, 59: pp. 202/451]. The speakers in Los Chenes either pattern with the speakers of Camino Real or with the speakers of the central/eastern regions of the peninsula. A discriminating property of this area is the use of *ba’al* as a negative quantifier, instead of *mix-ba’al* ‘negation-thing (= nothing)’ elsewhere. Finally, the variable *cucaracha* ‘cockroach’ illustrates a variable with three indigenous variants, namely *xooj* in the Southeast, *na’ts’ul* in central and western regions, and *k’uuruch/k’uuluch* in the Northeast (the latter is a Mayan word, already reported in the 18^th^ century in Diccionario de San Francisco [58: p. 424]). Along with the indigenous variants, the illustrative examples in Fig 6 display some Spanish variants that occur in the sample. These forms are Spanish words phonologically adapted to Yucatec Maya (Spanish stress is rendered as a long low-tone bearing vowel; open syllables at the word end are enclosed by a voiceless glottal fricative *j*). For instance, some speakers in the Northwest use the word *traankaj* (< Spanish *tranca*) for the ‘wooden bar’, speakers of various regions in the peninsula use the word *kukaraachaj* (< Spanish cucaracha) for ‘cockroach’, etc. Indigenous and Spanish variants come from different historical sources and may have different patterns of dispersion. Therefore, these variants will be examined in separate analyses in the following.

### Indigenous variants

#### Variation in geographical space

**Fig 7:**
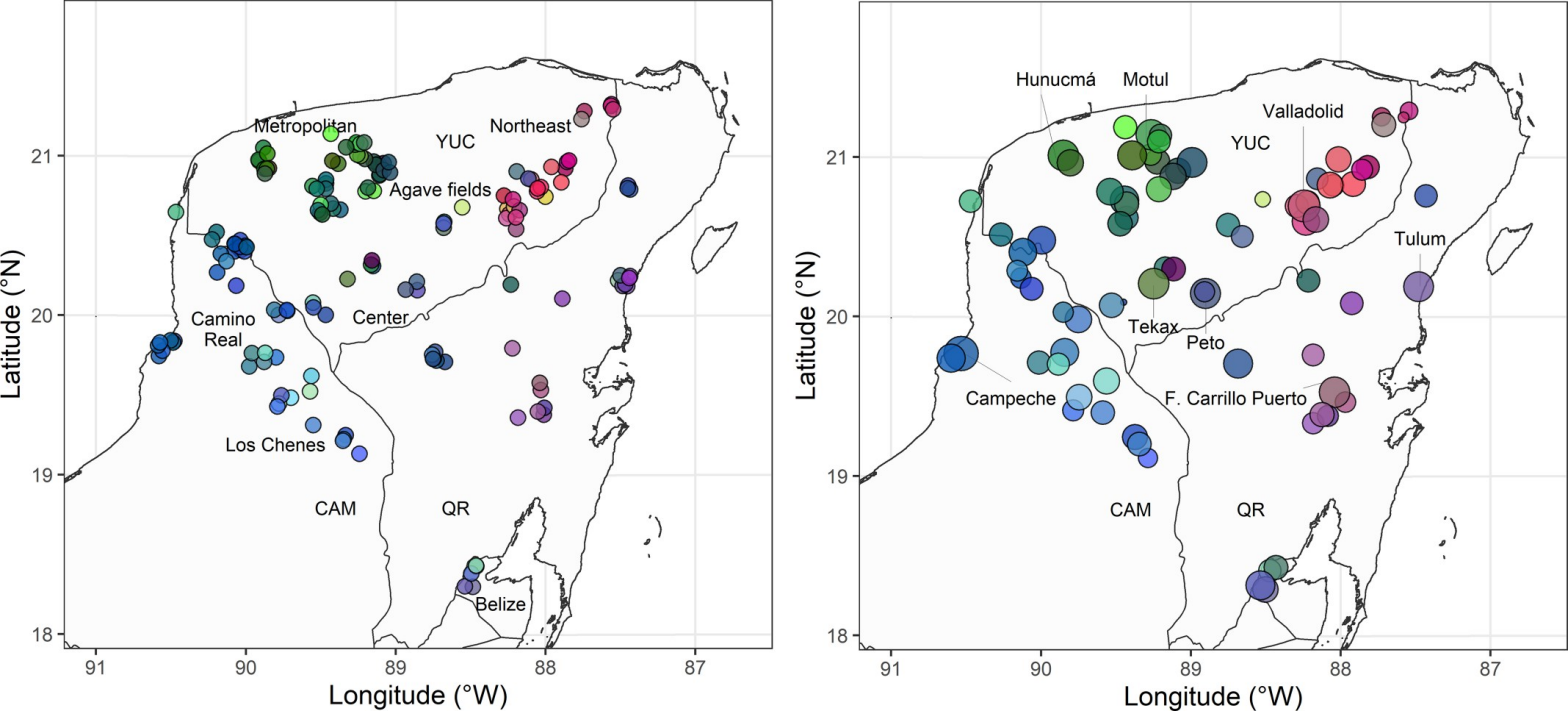
Estimates of multidimensional scaling mapped onto RGB colors. (A) Dots representing individuals. (B) dimensions resulting from multi-dimensional scaling aggregated per location; dot size: Logarithmized population size.

A dissimilarity matrix between speakers was computed with the indigenous variants by means of the procedure introduced in the ‘Data Analysis’. Reliability was tested by Cronbach’s *α* = .91, which indicates that the internal consistency is very high. The dimensions of the dissimilarity matrix were reduced to 3 dimensions by means of Classical Multidimensional Scaling (*R*^2^ = .61). The three dimensions of multidimensional scaling were mapped onto the three dimensions of RGB colors, offering a visualization of the distances in the color continuum [20,72,79]; see Fig 7A per speaker and Fig 7B per location. Dimension 1 is mapped onto red, dimension 2 to green, dimension 3 inversely to blue; mapping the direct or the inverse scores does not affect the distances between speakers [72, p. 157]. In these figures, similarity in color values between speakers reflects similarity in the choice of variants (small lexical distance).

Visual inspection suggests a major division between values of blue that are prevalent in Campeche (Camino Real and Los Chenes), values of green in northern/central Yucatán (Agave Area and western part of the Center), and values of red in the eastern part of the peninsula (with a difference between the northeast and the southeast). In order to inspect this result for possible effects of the urban centers (see predictions of the gravity model in Fig 5B), we present the same data aggregated per location in Fig 7B, in which dot size represents the population size of the corresponding community. Urban centers (large dots) generally share the color values of their respective areas, but we will statistically examine whether population size has some influence on the observed variation.

The exact linguistic variants that correlate with the color dimensions of Fig 7 were identified by means of logistic regressions, with the presence/absence of a variant as a dependent variable and the scores of the three dimensions of multi-dimensional scaling as independent variables in a linear model; [97]. The distance matrices (between speakers) of the single dimensions are weakly correlated to the input distance matrix (dimension 1, *R*^2^ = .33, dimension 2, *R*^2^ = .37, dimension 3, *R*^2^ = .33).

**Fig 8:**
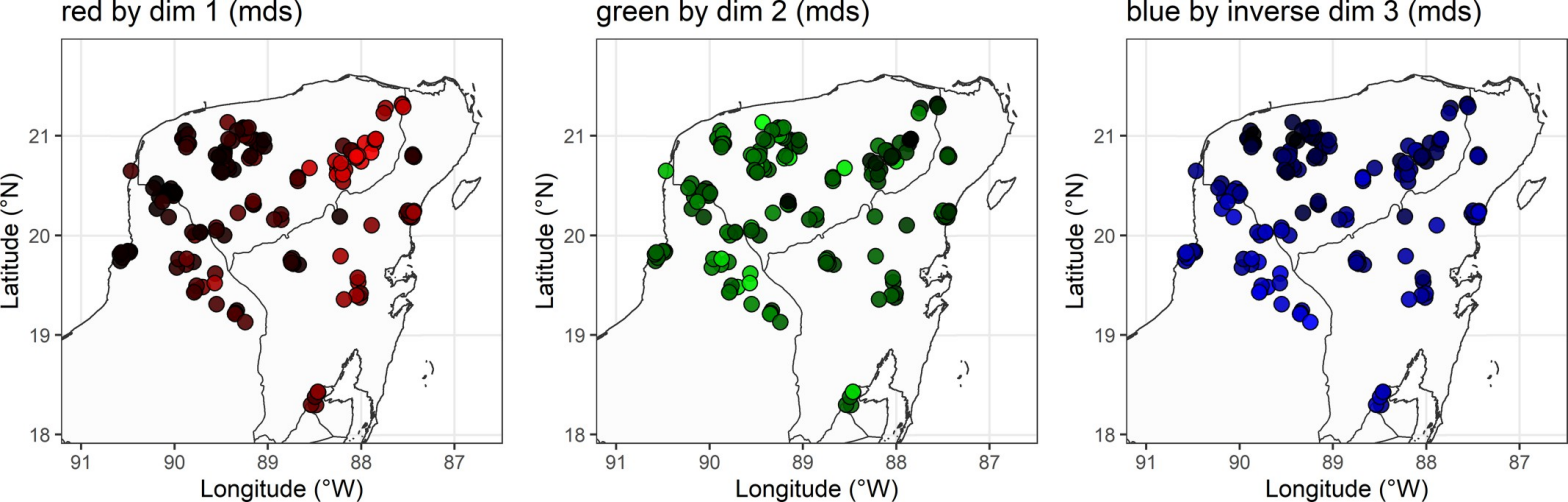
Dimensions of multidimensional scaling. The figures plot the scores of the speakers in the results of multidimensional scaling in color values. (A) red by dimension 1. (B) green by dimension 2. (C) blue by dimension 3.

The results in Table 1 show the five variants with the highest absolute *z*-scores per dimension: positive values correlate with the positive pole of the corresponding color dimension (red, green, blue, respectively), while negative values correlate with the negative pole of the same color dimension (black) (the values of the third dimension are inverted, so that the values of Fig 8 correspond to the plotted colors). The *z*-scores are the ratio of the estimate *β* of the logistic regression and its standard error, i.e., they offer an estimate of the strength of association between the dependent variable (probability of occurrence of a variant) and the corresponding independent variable (scores of the dimensions 1–3) divided by the standard error, which captures the variability of the data. Hence, the listed variants stand for the maximally discriminative variants of the plotted color dimension in the respective graph.

**Table 1:** List of the five variants per dimension which reached the highest absolute *z-*scores in the corresponding dimension in a logistic regression with the (non-) occurrence of a variant as a dependent variable and the three scores of multidimensional scaling as independent variables in a linear model (*n* speakers = 157; *n* observations = 6,304).

dimension 1		dimension 2		inverse dimension 3	
variable	variant	variable	variant	variable	variant
‘can’	*páajtal* *z* = –6.3, *p* < .001	‘drunk’	*kaal* *z* = –5.5, *p* < .001	‘protect’	*kanáant* *z* = 6.1, *p* < .001
‘herb’	*xiiw* *z* = –6.2, *p* < .001	‘sit down!’	*kulen* *z* = –5.4, *p* < .001	‘hawk’	*ch’uuy* *z* = 5.9, *p* < .001
‘annato’	*k’uxub* *z* = –6.2, *p* < .001	‘quickly’	*séeb* *z* = –5.3, *p* < .001	‘potbellied’	*p’urux* *z* = 5.8, *p* < .001
‘home’	*naj* *z* = 6.1, *p* < .001	‘teach’	*ka’ans* *z* = –4.9, *p* < .001	‘dough’	*juuch’* *z* = 5.6, *p* < .001
‘nest’	*k’u‘* *z* = –5.9, *p* < .001	‘scrambled’	*xa’ak’a’an* *z* = –4.9, *p* < .001	‘protect’	*kaláant* *z* = –5.6, *p* < .001

#### Determinants of similarity

The factors determining variation in this data were examined with a linear mixed-effects model on the Dissimilarity between pairs of speakers as a dependent variable. The examined predictors were Geographic Distance (logarithmized distance between locations in km), Time (product of the year of birth of the speakers), Population size (product of the logarithmized population sizes of the communities of both speakers), and Indigenous Population (product of the percentages of indigenous speakers in the communities of both speakers). A correlation matrix (Pearson’s correlations) between the fixed effects reveals that the highest correlation (between Indigenous Population and Population Size; *r* = -.58), does not reach the threshold .7 for ‘high’ correlations.

The estimates, standard errors and *t*-values of the fixed-effects of the model are listed in Table 2. We applied a stepwise procedure of forward model selection integrating all relevant main effects. Interaction effects were not considered in the interest of simplicity and since the argumentation of the present study relates to the direction of the main effects; hence, the model in Table 2 is a ‘minimally’ adequate model; see [19] for a previous study with this approach. The improvement in the informativity of the model is assessed by the AIC (= Akaike Information Criterion) decrease, which is the difference of the AIC value of a model with the factor at issue minus the AIC value of a model without this factor. An AIC decrease means that the informativity of the model increases, estimating the trade-off between the goodness of fit of the model and the number of parameters applied to explain the data. Model selection was based on the results of an analysis of variance between models, whose *F*-values and the corresponding *p*-values are listed in the two last columns of Table 2.

**Table 2:** Linear mixed-effects model on the Dissimilarity between speakers (*n* = 12,077).

coefficients	estimate	SE	*t*-value	model comparison		
				AIC-decrease	*F*	*p*
Intercept	.470	.015	30.863			
Geographic Distance (log_2_)	.237	.014	16.624	–712.7	736.2	.001
Time	.021	.009	2.254	–51.8	53.9	.001
Population Size (log_2_)	–.098	.016	–5.990	–476.3	487.7	.001
Indigenous Population (%)	.043	.008	5.301	–148.9	151.8	.001

The findings indicate that Dissimilarity increases along with Geographic Distance, which is in line with the *Fundamental Dialectological Postulate* in [6]. The negative effect of Population Size indicates that Dissimilarity decreases in and between larger centers, which provides evidence for convergence processes in these centers. Indigenous Population has a significant impact on Dissimilarity, i.e., linguistic divergence increases in areas with higher proportion of Yucatec Mayan population. Finally, the significant effect of Time (birthyear of the speakers) indicates that Dissimilarity increases over time, such that younger speakers have more differences to each other than older speakers, which is evidence for increasing divergence.

#### Choice of variant

**Fig 9:**
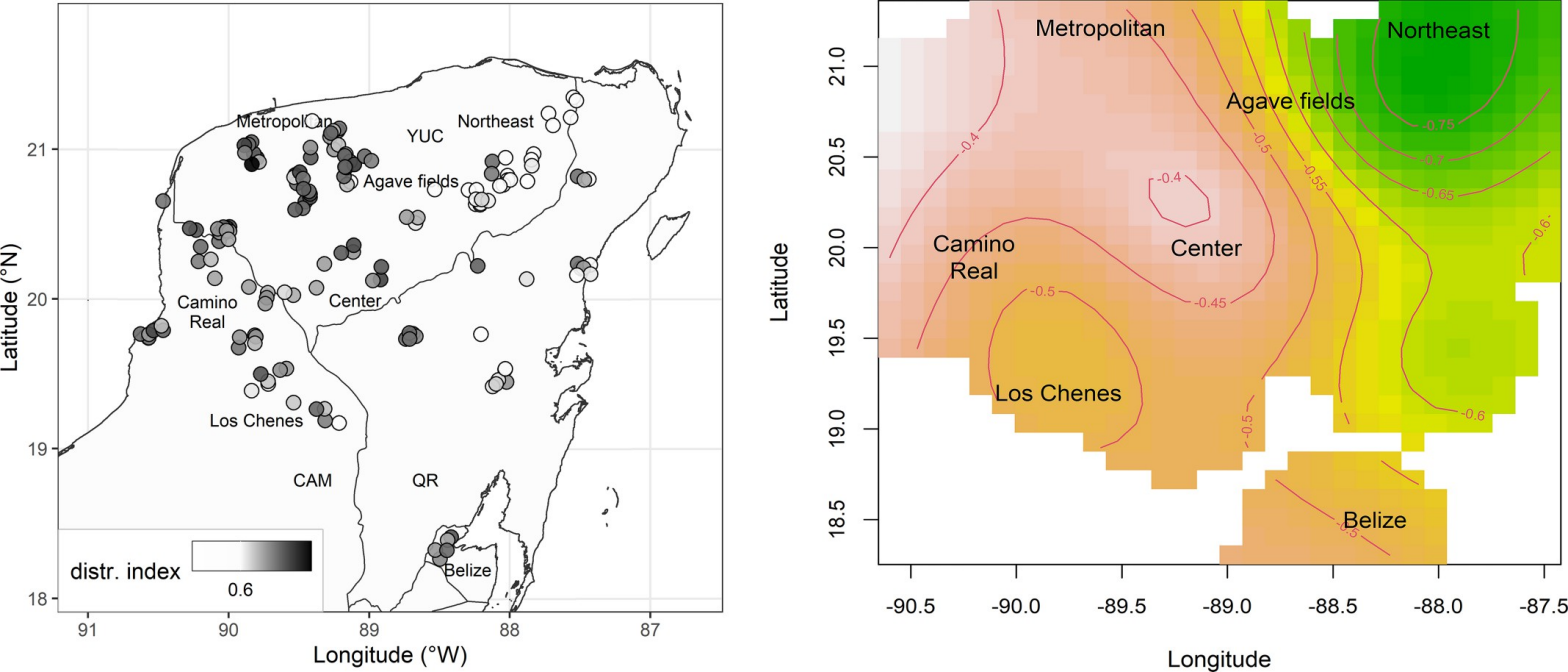
Diffusion of indigenous variants in the population. (A) Average distribution index per speaker. (B) Contour plot of Space coefficients (estimates of the smooth term in GAM).

In order to assess the distribution of the variants, we calculated the Distribution Index of each variant (i.e. its relative frequency; see ‘Choice between indigenous variants’ in the Section ‘Method’). The average distribution indices per speaker are plotted in Fig 9A. These averages show to what extent the speaker selected variants with wide distribution in the population (high average). The dispersion of these averages in geographical space reveals that variants with wider distribution are more frequent at the center and the Northwest (white areas), while the variants with narrow distribution are more frequent in the East as well as in the Southwest (green areas).

A generalized additive mixed-effects model was fitted to the Distribution Indices of all obtained indigenous variants. This model assessed the properties of a dependent variable that ranges within a standard unit interval (0,1) with a beta regression. The factor Space is a (non-linear) interaction of the coordinates (longitude and latitude) fit by a thin plate regression smoother. The random factor Concept captures the variation due to the 52 alternative target concepts. The examined parametric fixed factors were Time (birthyear of the speaker), Population size (logarithmized population size in speaker’s community), and Indigenous Population (percentage of indigenous speakers in speaker’s community). All variables were rescaled to a [.001, .999] interval in order to meet the requirement of the beta regression that the values of the dependent variable fall within a (0,1) interval. The correlation matrix between the parametric fixed factors reveals that the correlation coefficient is below the threshold for high correlations (|.7|) in all permutations (highest correlation: Indigenous Population and Population Size, *r* = -.67).

The coefficients of Space (Fig 9B) display the values of the non-linear parameters of longitude/latitude (‘smooth term’ of Space) that achieve maximal accuracy with least complexity–given the amount of variation that is explained by the further factors of the model. Similarly with the descriptive facts (Fig 9A), the coefficients of Space in Fig 9B show a peak at the center and the northwestern part of the peninsula and a valley in the eastern area, i.e., variants with limited distribution more often occur in the east. The number of base functions for assessing the smooth term of Space was set to *k* = 15; the obtained degrees of freedom (10.3 in Table 3) indicate that the granularity of the adopted number of functions was sufficient for assessing the variation in the data (since the effective number of base functions that capture the non-linearity of the facts is lower than the adopted parameter *k*). The model fit was significantly improved by including a non-linear factor of Space, as seen by the significance level (*p* < .001).

**Table 3:** Generalized additive mixed-effects model of maximal fit on the distribution indices of the selected variants (*n* = 6,304).

parametric coefficients	estimate	SE	*z*-value	model comparison		
				AIC-decrease	*χ* ^2^	*p* <
Intercept	.740	.121	6.114			
Time	–.137	.062	–2.188	–2.69	2.391	.05
Population Size (log_2_)	–	–	–	1.13	.765	–
Indigenous Population (%)	–	–	–	.62	1.225	–
smooth terms	eff. df	ref. edf	*χ*^2^-value	*p* <		
Space	10.3	12.4	119.4	.001		
Concept	50.5	51.0	5045.7	.001		

Model comparison started with a model containing the smooth term of Space and the random effect of Concept. This model was augmented with adding the parametric fixed-factors and testing their significance in a Log-Likelihood Test (forward model selection procedure). The maximal goodness of fit (minimal AIC value) was reached by a model with the fixed factor Time; see Table 3. The negative effect of Time indicates that the birthyear is inversely correlated with the distribution index of the variants, which is evidence that younger speakers more often selected variants with limited distribution. The factors Population Size and Indigenous Population did not reach significance.

The results of the generalized additive mixed-effects model show that the major determinant of variants with wider distribution is Space, since there is a bias for variants with wider distribution in the center and the northwest of the peninsula (Fig 9B), i.e., in the areas that are the most important nodes of the mobility axes. Furthermore, variants with wider distribution are less frequent in the data from young speakers. There is no evidence that Population Size and proportion of Indigenous Population influence the choice of variants with wide/narrow distribution in the population.

### Spanish variants

We consider as ‘Spanish variants’ forms with Spanish origin that may be adapted to the Yucatec Maya phonology. These variants are sometimes identical to the Spanish prompt, e.g., *traankaj* as a translation of the Spanish prompt *tranca* ‘wooden bar’, or different Spanish words that are integrated to Maya, as for instance the conjunction *aastaj* (< Span. *hasta*), which is used as a general temporal conjunction in Yucatec Maya, and also occurs as a translation of the Spanish preposition *cuando* ‘when’ (prompt: *cuando vengas*, *vamos* ‘when you will come, we will leave’). In the sample of the present study, we encountered 171 Spanish tokens (2.6% out of total valid 6,475).

Since speakers were instructed to translate the Spanish prompts into Yucatec Maya (see instruction under ‘data collection’), they generally refrain from using Spanish variants unless they cannot find an adequate translation in Yucatec Maya or unless the Spanish expression is the most frequent way for rendering the propositional content in the everyday communication (as it is the case, e.g., with higher numerals in most Mayan languages). The frequency of Spanish words in our data is lower than the frequency of Spanish in spontaneous communication: a corpus study on conversation data reports 1,253 Spanish tokens in a total corpus of 13,345 words (9%) [47: p. 372]. This difference confirms the intuition that a translation task has a bias towards avoiding the element in the prompt. Data obtained by translation is rather informative for the lexical competence than for the language use of the speakers.

**Fig 10:**
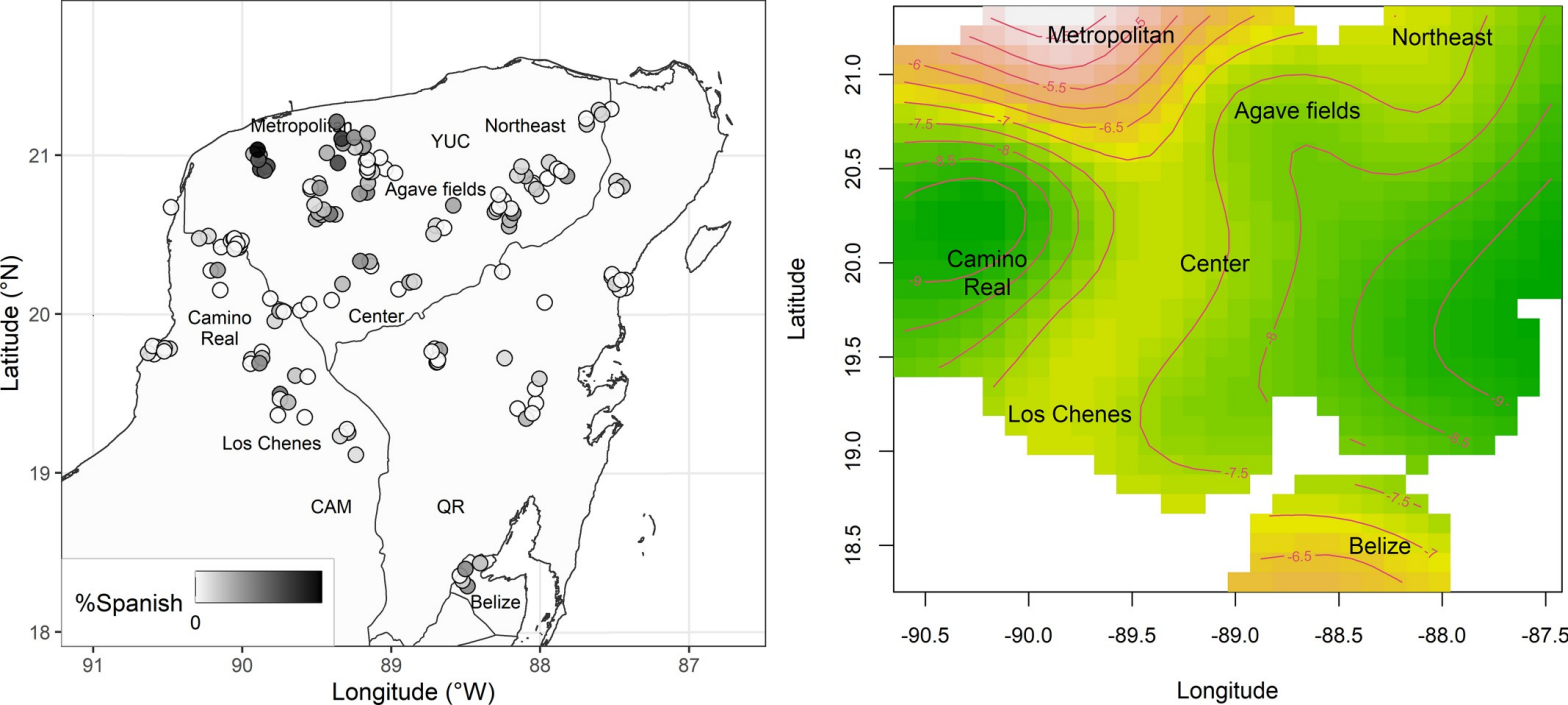
Distribution of Spanish variants in space. (A) Average Spanish variants per speaker. (B) Contour plot of Space coefficients (estimates of the smooth term in GAM).

The consistency across concepts was assessed with Cronbach’s alpha, which indicated a high reliability (*α* = .76) (five concepts had to be excluded due to missing values). The average Spanish variants per speaker are plotted in Fig 10A, which reveals an areal bias: Spanish variants most frequently occur in the Metropolitan area, which is the area with lowest density of indigenous speakers; see Fig 1A.

A generalized additive mixed-effects model was fitted on the variable Language (Maya or Spanish), capturing the origin of the transcribed entry (0 = Maya; 1 = Spanish). The factorial structure contained the smooth term Space (non-linear interaction of the geographical coordinates fit by a thin plate regression smoother), the random factor Concept (relating to the 52 target concepts) and the parametric fixed factors Time (birthyear of the speaker), Population size (logarithmized population size of the community), Indigenous Population (percentage of indigenous speakers of the community). All variables were rescaled to a [.001, .999] interval. The correlation analysis reveals that Pearson’s coefficient does not reach the threshold for very strong correlations in the combinations between fixed factors (highest correlation: between Indigenous Population and Population Size, *r* = -.674).

The coefficients of Space in Fig 10B confirm the peak of the use of Spanish variants in the northwestern part of the peninsula. Starting with a model only containing the smooth term Space and the random factor Concept, we identified the model of maximal fit in Table 4 by means of forward model selection. The significant effect of TIME indicates that the use of Spanish variants increases with younger speakers. The further factors (Population Size, Indigenous Population) do not significantly increase the informativity of the statistic model.

**Table 4:** Generalized additive mixed-effects model of maximal fit on the occurrence of Spanish variants (*n* = 6,475).

parametric coefficients	estimate	SE	*z*-value	model comparison		
				AIC-decrease	*χ* ^2^	*p* <
Intercept	-6.918	.645	-10.728			
Time	1.348	.559	2.412	–3.95	2.98	.05
Population Size (log_2_)	–	–	–	1.94	.001	–
Indigenous Population (%)	–	–	–	1.85	.007	–
smooth terms	eff. df	ref. edf	*χ*^2^-value	*p* <		
Space	12.06	13.48	132.5	.001		
Concept	36.44	51.00	332.1	.001		

The generalized additive mixed-effects model shows that a major determinant of the choice of Mayan and Spanish variants is Space, with a bias for Spanish variants in the Metropolitan area. The significant positive effect of Time shows that Spanish variants are more likely with younger speakers, but there is no evidence that Population Size and Indigenous Population have an influence on the choice of Spanish variants in this data.

## Discussion

Contingency in geographical space is an important predictor of lexical variation in Yucatec Maya, which adds evidence to a long paradigm of studies on the diffusion of dialectal variants (see [5,9] for various languages and [1,11] for previous research on rural societies). The lexical distance between speakers is better captured by the logarithmized geographical distance (Fig 3), which confirms the view that linguistic distance is a sublinear function of geographical distance, such that divergence increases rapidly in close distances and less rapidly in remote distances [5–11]. The aggregation of the variants in Fig 7 reveals a gradient division between (a) the speakers of Campeche (Camino Real and Los Chenes), as originally suggested by [34], (b) the speakers of northern and central Yucatán (Agave Area and western part of the Center), and (c) the eastern regions, including the Northeast and the eastern part of the Center (Quintana Roo), as already suggested in earlier studies [55,62,63].

Two major aspects of the language situation at issue are (a) the diglossia-with-bilingualism with Spanish and (b) the absence of a standard variety. Under these circumstances, the spread of indigenous variants can only be accounted for by processes of horizontal convergence. Variants with limited distribution occur more frequently in the eastern part of the peninsula (Fig 9). The dispersion of local variants in this area is in line with the historical fact that this part of the peninsula was less accessible up to the beginning of the 20^th^ century, while the central and western parts were more accessible in terms of road connections and mobility. Variants with a wider distribution in the population occur more often in the center and northwestern part of the peninsula. These two areas share in common that they have the largest array of connections to other areas. The center coincides with the area of Peto in the southern part of the state of Yucatán, which is an important mobility node connecting the eastern part of the peninsula (Quintana Roo) to the areas in the state of Yucatán. The northwestern area is crucial for mobility, since this is the area around the capital of Merida, connecting Campeche, Quintana Roo, and Yucatán. In conclusion, widespread variants are more likely in the nodes of mobility, which is in line with processes of homogenization due to horizontal convergence.

In the language situation at issue, urban centers are not the hosts of linguistic/cultural prestige for the indigenous culture (see also [46] on Mayan languages in general). This means that urban centers host processes of horizontal convergence (since speakers of various local varieties interact in these centers), but not of vertical convergence (due to prestige). Our data reveals that Population Size had a negative impact on the Dissimilarity between speakers (Table 2), which means that similarity increases in and between urban centers. Previous studies have shown that dialect levelling is weaker in languages spoken in environments with a language of public life that differs from the local vernaculars, as e.g., the Catalan of Aragon [87], or the Dutch of Belgium [82]. In Yucatán, the indigenous population is well represented in the urban centers and the indigenous language is actively used in various occasions of the everyday life, e.g., in marketplaces and cultural events–even if it is not the language of the administration. The vivid language use in the larger centers is a possible source of processes of horizontal convergence (in and between urban centers), that accounts for the effect of Population size on reducing Dissimilarity between speakers in our data (Table 2). We did not find evidence that Population size affects the occurrence of the variants of wider distribution. This result means that the distribution of these variants is captured by the areal biases discussed in the previous paragraph: they are better represented in the areas that represent the most important nodes of mobility in the peninsula, which means that have the largest amount of contacts with other areas.

The factor Time captures the reflexes of language change on the apparent time scale of the birthyears of the speakers in our sample (1906–1989). In our results, Time had a significant effect on the Dissimilarity between speakers (Table 2), which indicates that divergence increases over time. This result is in a sharp contrast to findings of studies reporting that dialectal differences are levelled out for younger speakers such that dialectal varieties converge with time; see [20] on Swedish, [82] on Dutch. The occurrence of variants with wider distribution involves a negative effect of Time, which indicates that younger speakers more often use variants of limited distribution in this data (Table 3). This result is in line with the lack of standardization processes in this language situation; the preference for variants of limited distribution hints to a tendency to reassert local accents; see [22]. Recall that the new wave of individuals seeking for the *jach maaya* ‘pure Maya’, as described in [44,45], predicts a tendency towards local variants. The speaker sample examined in the present study is representative for the maximally competent speakers of the sample locations, born in a period in which the language was still used by a substantial part (more that 20%) of the population in the peninsula (see percentages of the year of birth range 1906–1989 in Fig 1B). This sample is not informative for recent developments that are associated with the radical shrinkage of the speaker population or the consequences of institutionalization (through the representation of Yucatec Maya in school education).

The distribution of Spanish variants in the peninsula is accounted for through the geographical bias for Spanish variants in the Metropolitan area and an effect of Time, such that Spanish words are more likely with younger speakers (Table 4). In the language situation at issue, Spanish borrowings are expected to be dispersed by means of processes of vertical convergence (towards the institutionalized language), starting with urban centers in which Spanish is better represented and spreading to their satellites. This expectation is not confirmed by the data, since Population Size and the proportion of Indigenous Population did not have a significant effect (Table 4). The illustrative Spanish variants in Fig 6 are informative for this data pattern, especially the spatial distribution of the variants *traankaj* ‘wooden bar’ and *kukaraachaj* ‘cockroach’. The areal bias suggests that these translations are not spontaneous replacements by speakers who do not recall the Yucatec Mayan words, but rather Spanish borrowings that have been established in certain regions. The fact that these borrowings appear in the Metropolitan area is not accidental, since this area has a longer history of language contact to Spanish due to labor migration already in the 19th century; see [98] on areal biases in borrowings.

## Conclusions

The present study analyzed the lexical variation in Yucatec Maya with the aim of identifying reflexes of the language situation in the distribution of lexical variants. This language is spoken in a situation of diglossia-with-bilingualism and does not have a standard variety (since the language of education and administration is Spanish). Processes of vertical convergence between varieties of Yucatec Maya are not applicable in this language situation, but they may apply to the relation of local vernaculars with Spanish (as reflected in Spanish borrowings).

Our aim was to draw inferences from the effects of the gravity model to the properties of the language situation at issue. We found evidence for convergence processes in and between urban centers (effect of Population Size on the decrease of Dissimilarity), which can only be due to processes of horizontal convergence, since this language is actively used in the everyday life in the urban centers of the peninsula. Furthermore, variants with wide distribution in the peninsula more often occur in the areas that have the greatest amount of interactions with other areas, which are the central area of Peto and the Metropolitan area. This distribution is explained by processes of horizontal convergence since these areas share in common that they are important nodes connecting the different parts of the peninsula. Our study involves the surprising findings that (a) divergence increases with younger speakers and (b) younger speakers preferred variants with limited distribution in the peninsula. Presumably, this result may be due to the emerging trends in the speech community to reinforce the local vernaculars seeking for an ‘authentic’ use of Yucatec Maya. In view of the further findings of this study, the absence of evidence for increasing convergence during time fits to the overall picture that processes of dialect levelling do not apply to a population that did not learn the language at school and is not influenced by an established standard variety. Finally, the distribution of Spanish variants does not reveal an effect of Population Size: the distribution of these variants in the sample is mainly explained by the areal bias, which indicates that most variants in the dataset are established borrowings in certain areas.

## Supporting information

S1 File Feature distribution in space (pdf file). (PDF)

S2 File (PDF)

S3 File Data processing, visualizations, statistical analyses (R markdown file). (RMD)

S1 Data Forms, speakers, locations, prompts (excel spreadsheets). (XLSX)
