# Supplementary material for: Sources of convergence in indigenous languages: Lexical variation in Yucatec Maya
Source: PLoS One. 2022 May 19;17(5):e0268448. doi: 10.1371/journal.pone.0268448 (PMC9119476; doi:10.1371/journal.pone.0268448)

Gravity models and sources of convergence in indigenous languages: Lexical variation in Yucatec Maya

S1 Supplementary Material: Features in Space

Fig 1

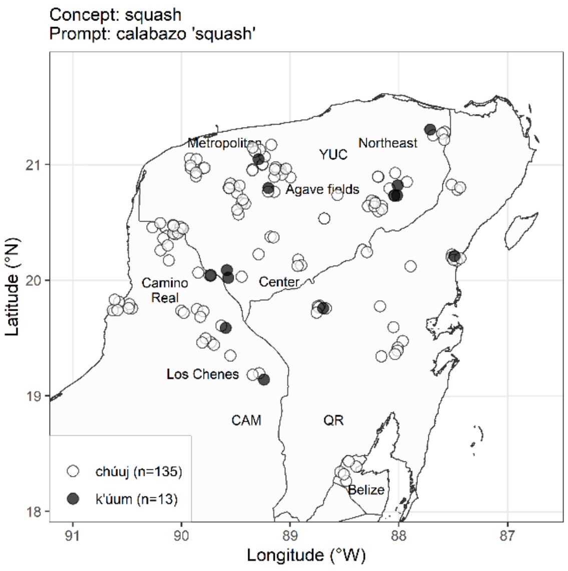

Fig 2

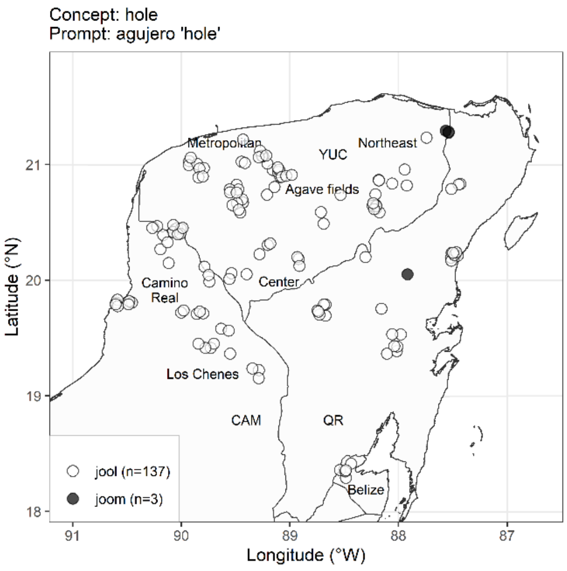

Fig 3

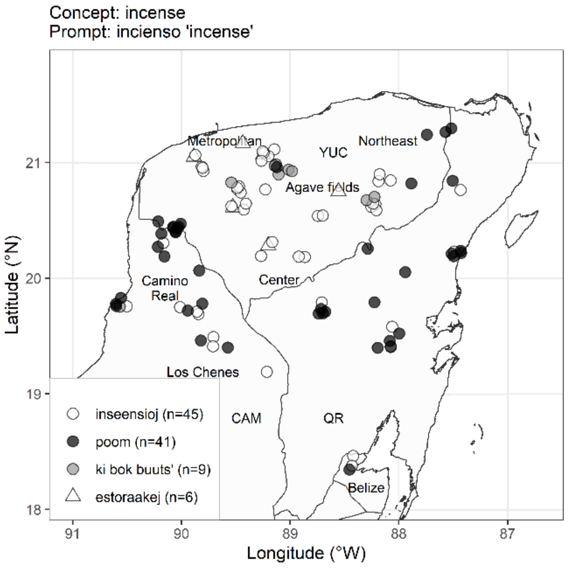

Fig 4

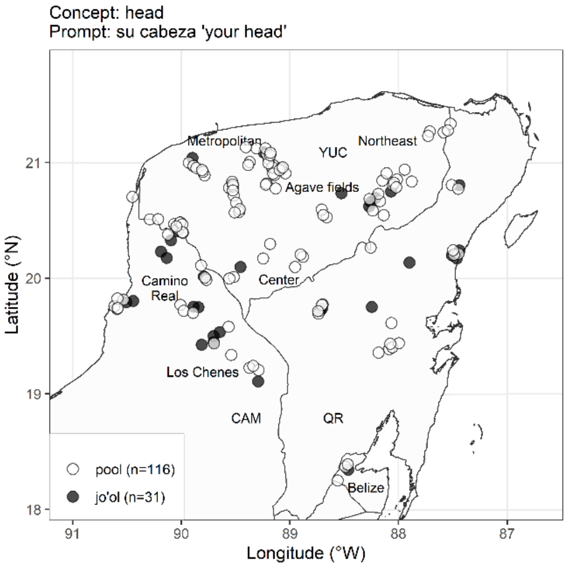

Fig 5

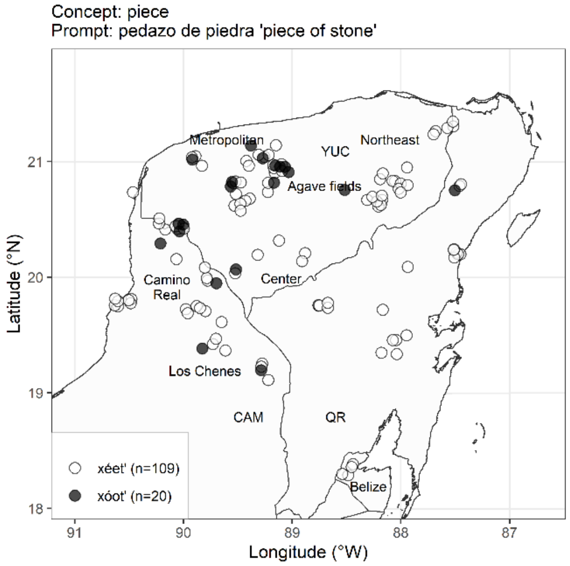

Fig 6

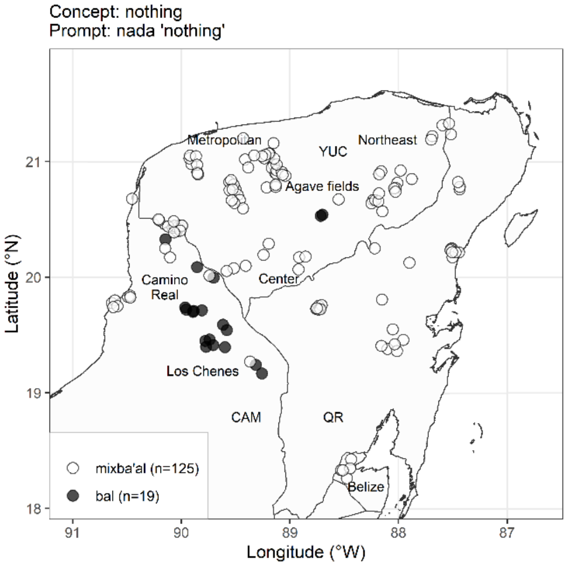

Fig 7

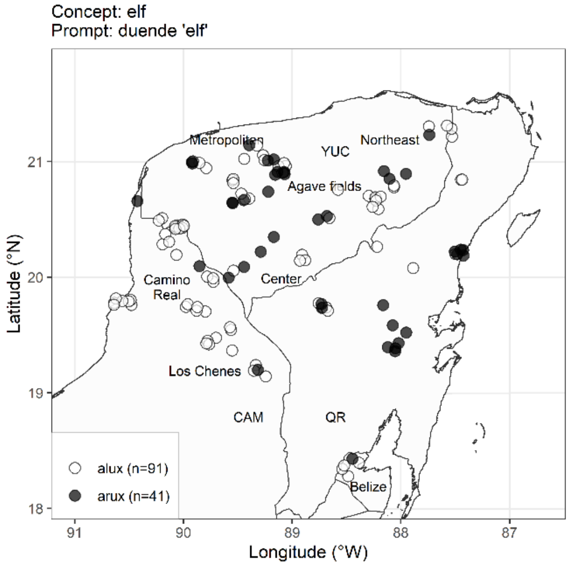

Fig 8

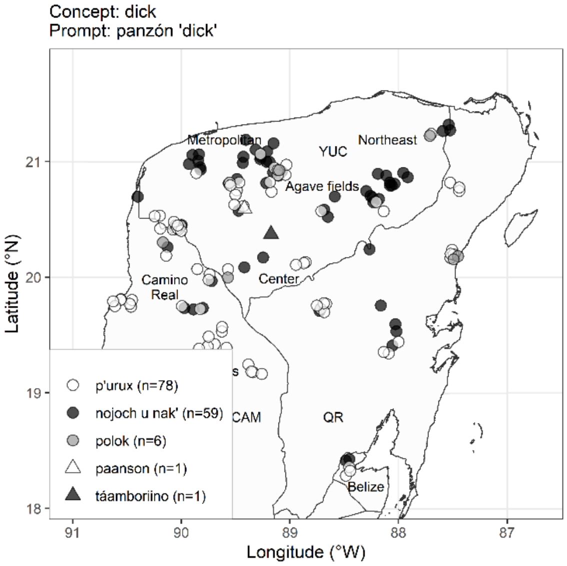

Fig 9

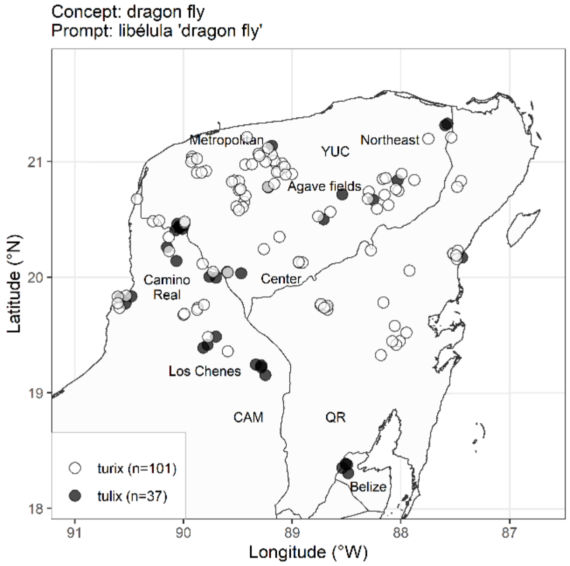

Fig 10

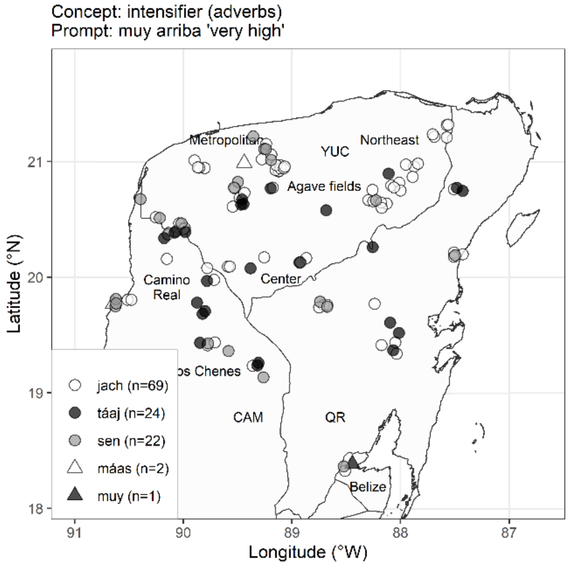

Fig 11

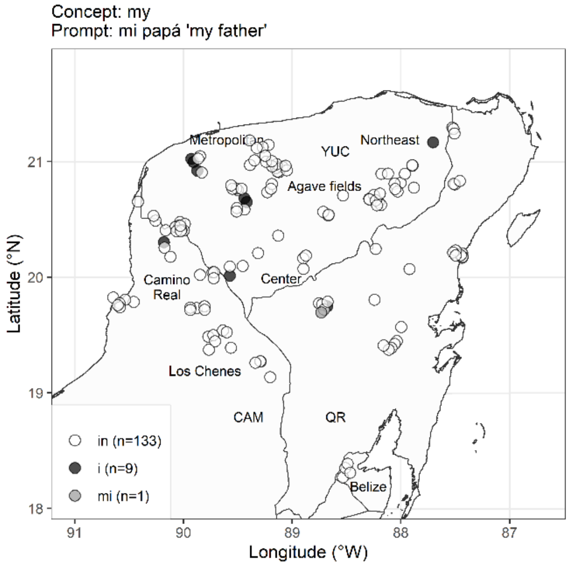

Fig 12

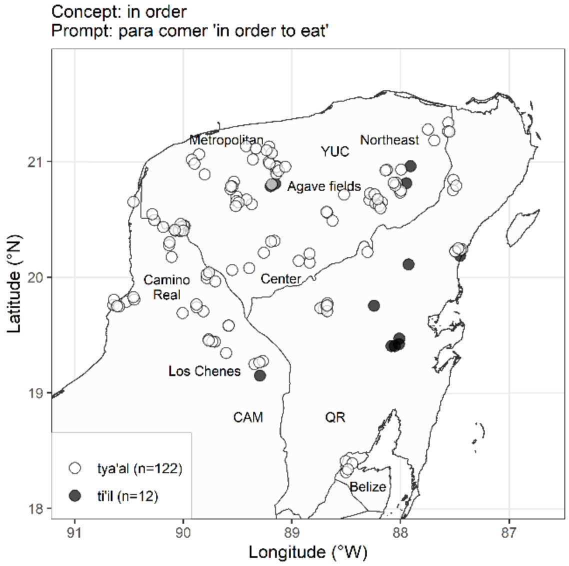

Fig 13

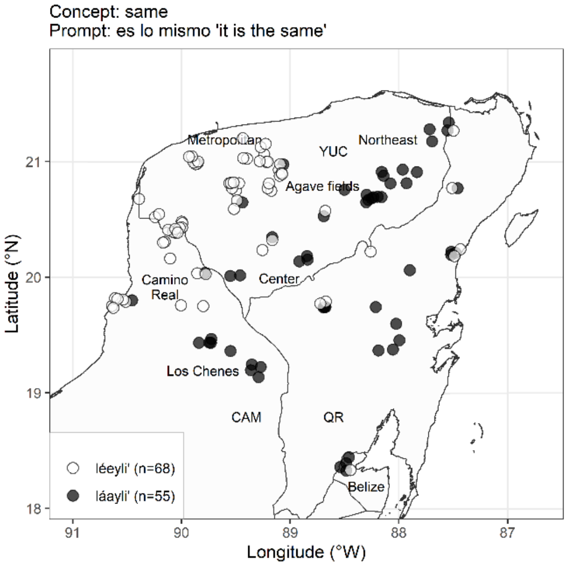

Fig 14

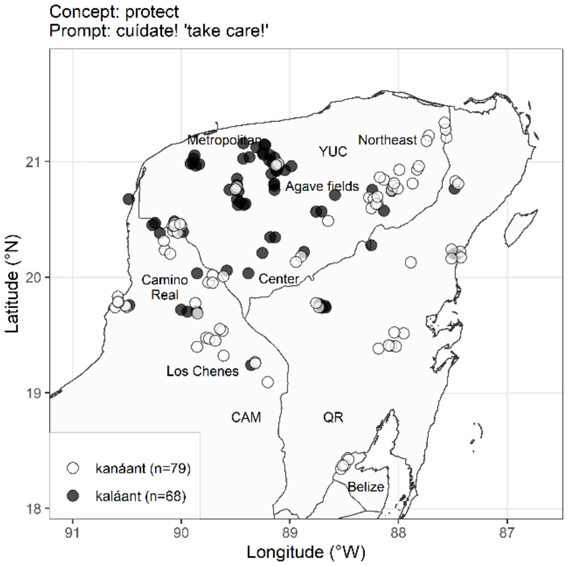

Fig 15

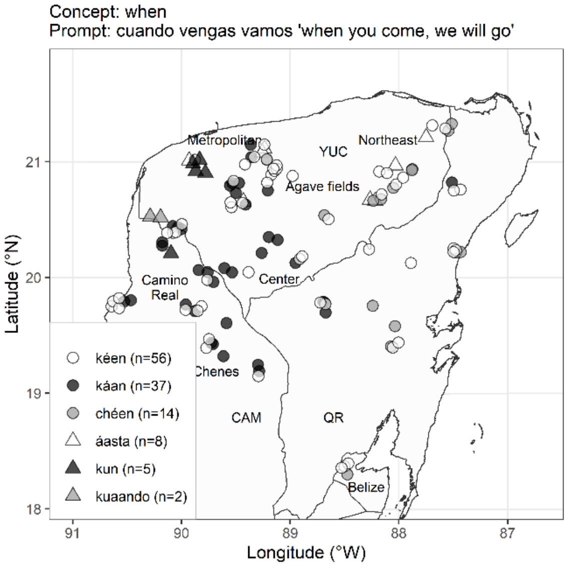

Fig 16

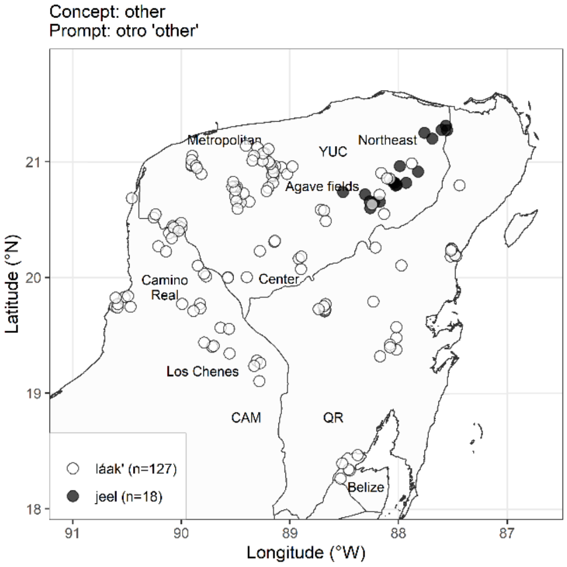

Fig 17

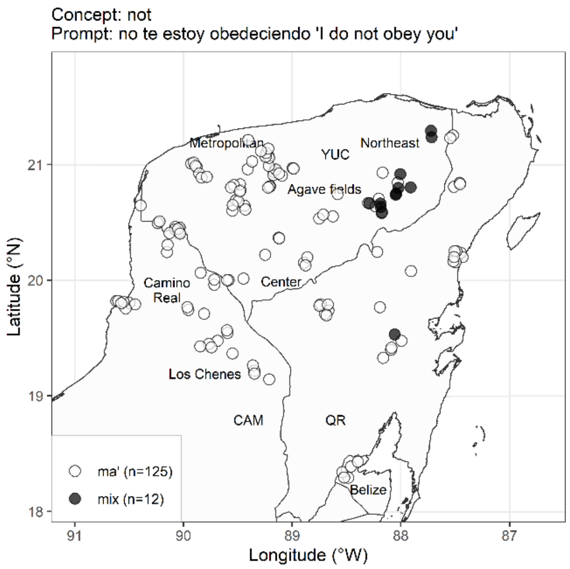

Fig 18

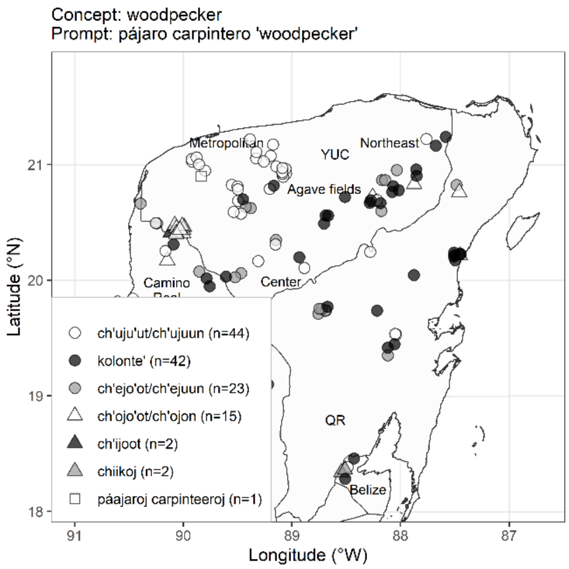

Fig 19

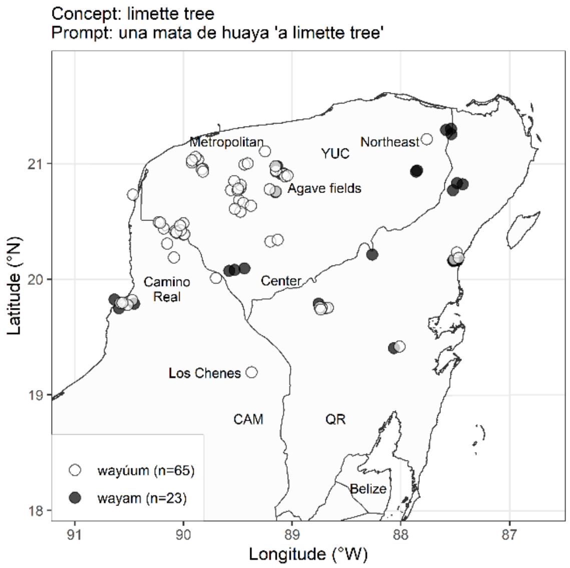

Fig 20

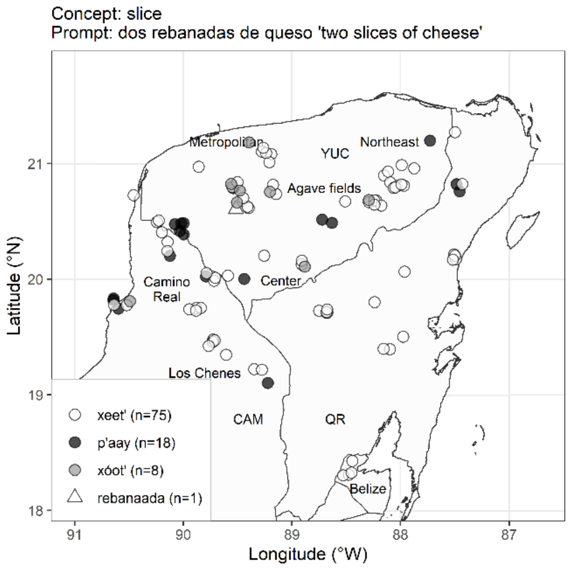

Fig 21

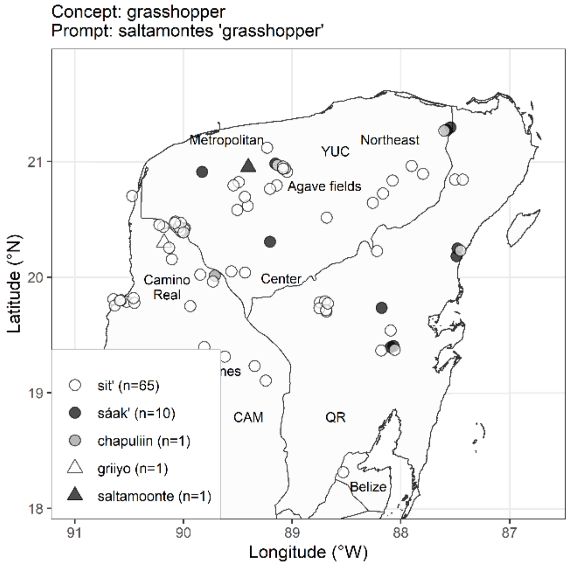

Fig 22

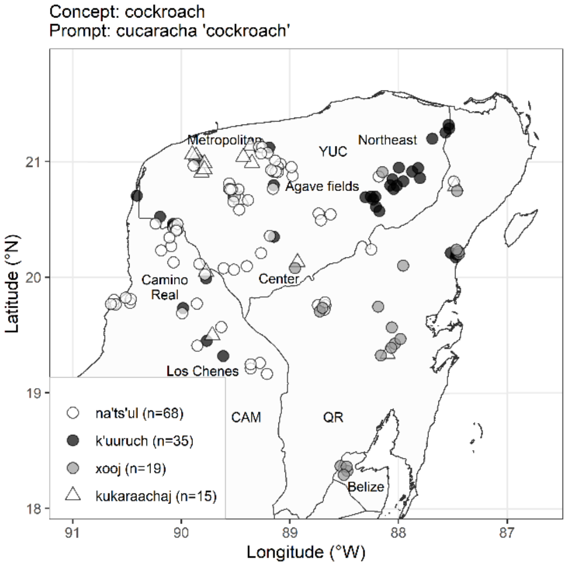

Fig 23

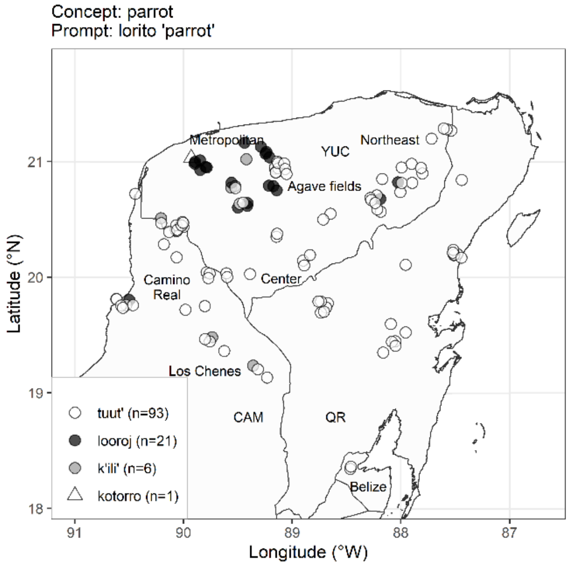

Fig 24

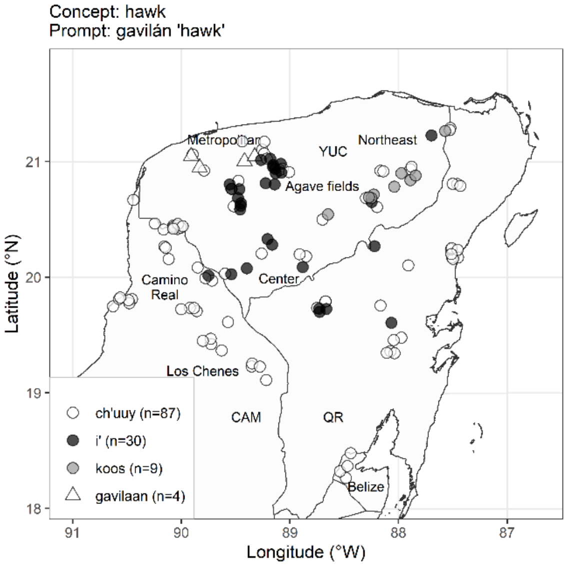

Fig 25

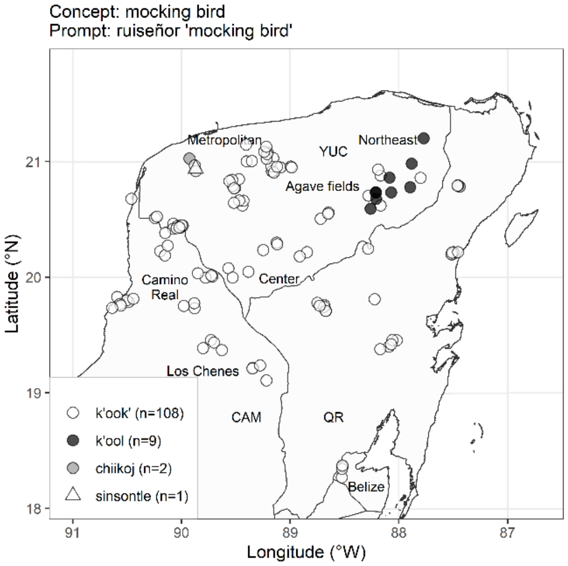

Fig 26

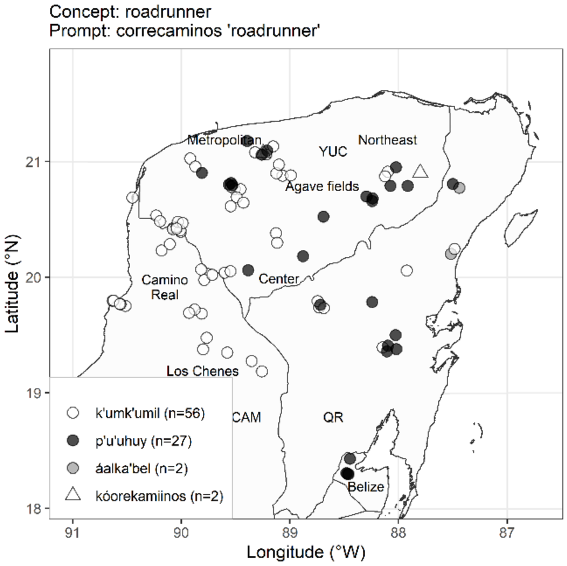

Fig 27

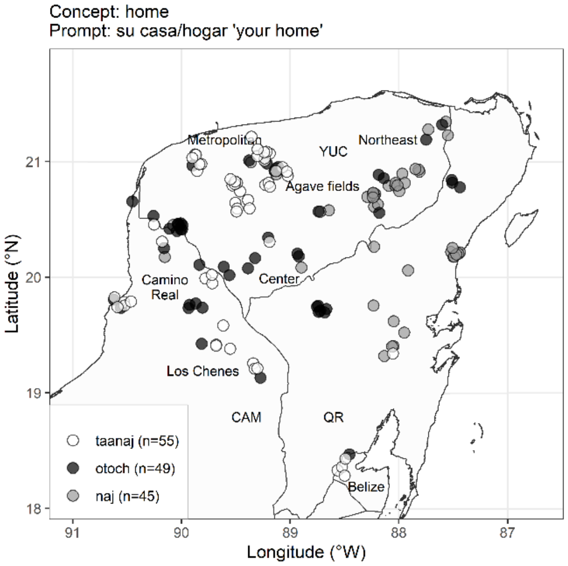

Fig 28

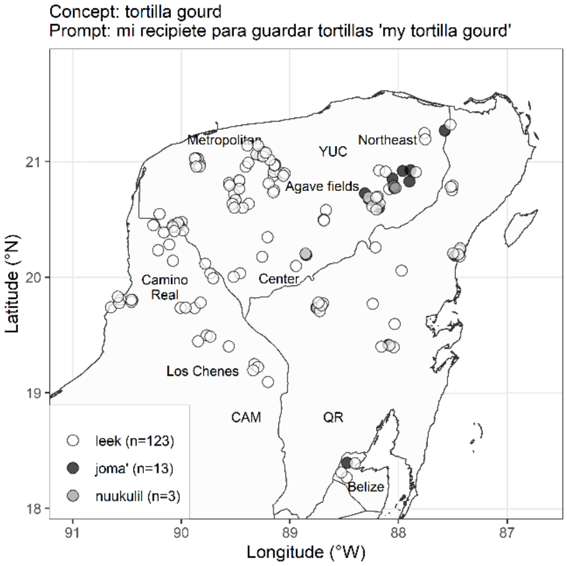

Fig 29

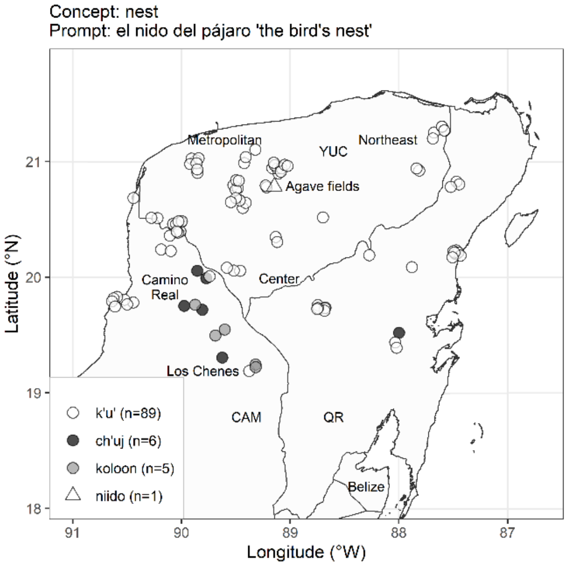

Fig 30

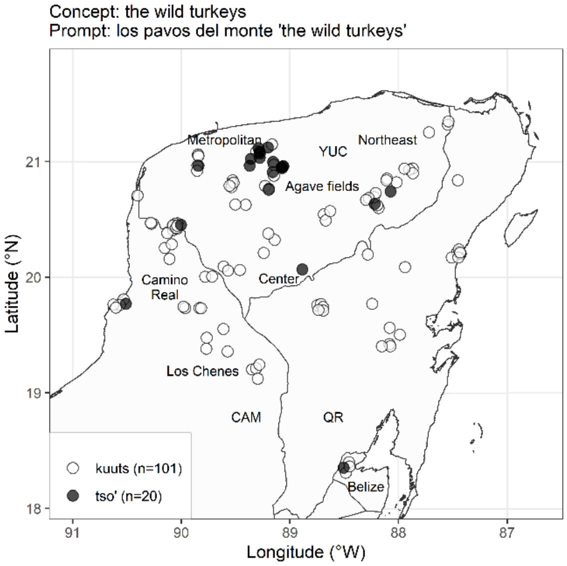

Fig 31

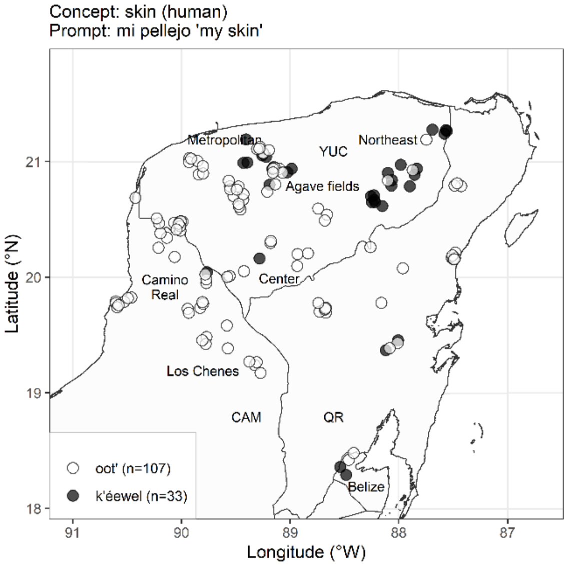

Fig 32

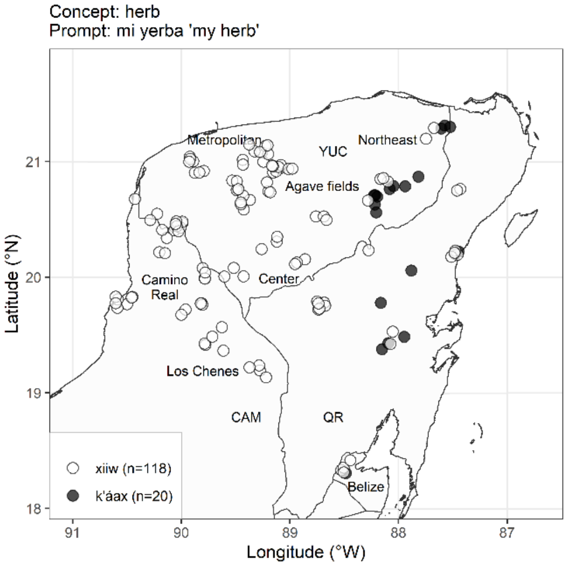

Fig 33

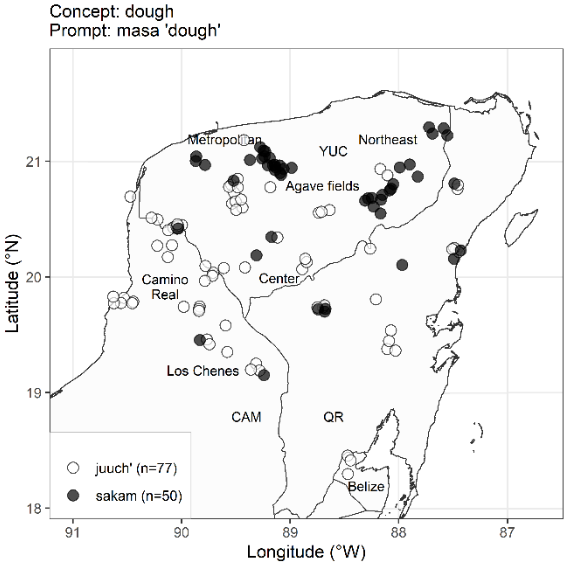

Fig 34

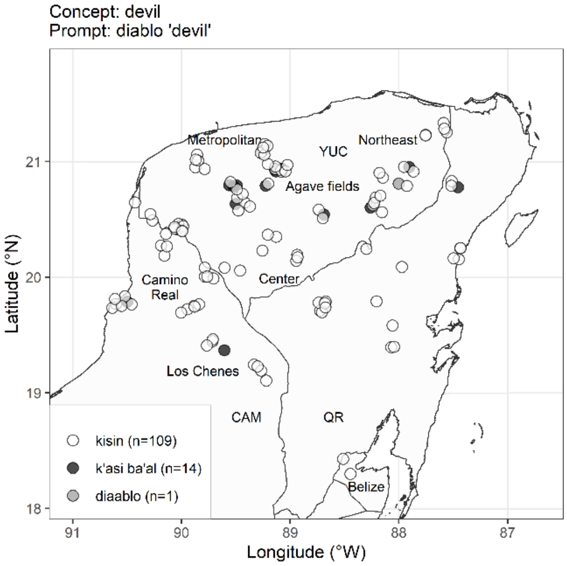

Fig 35

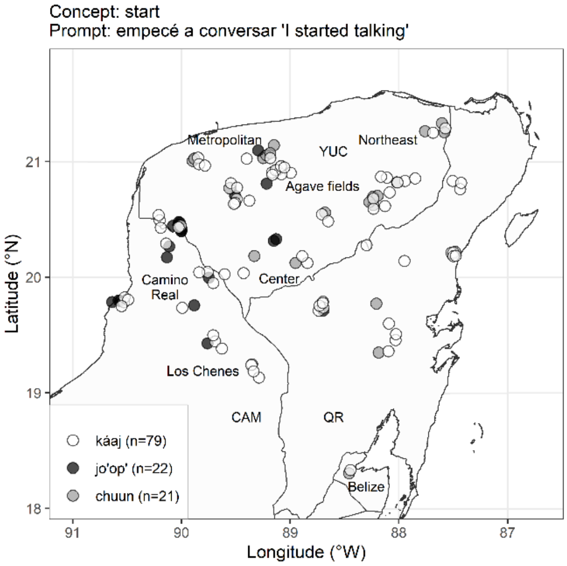

Fig 36

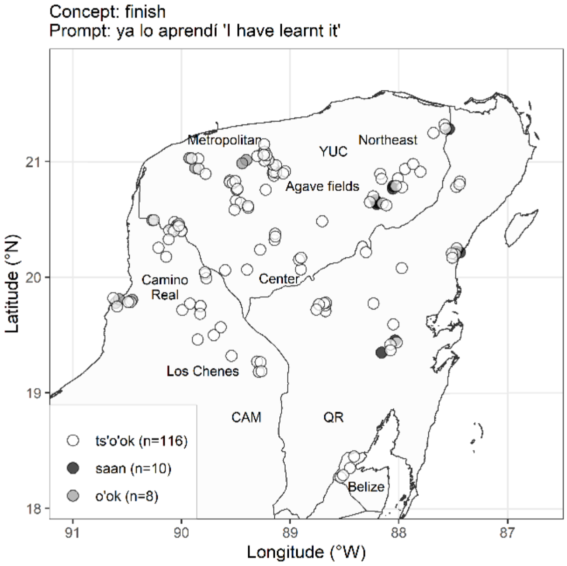

Fig 37

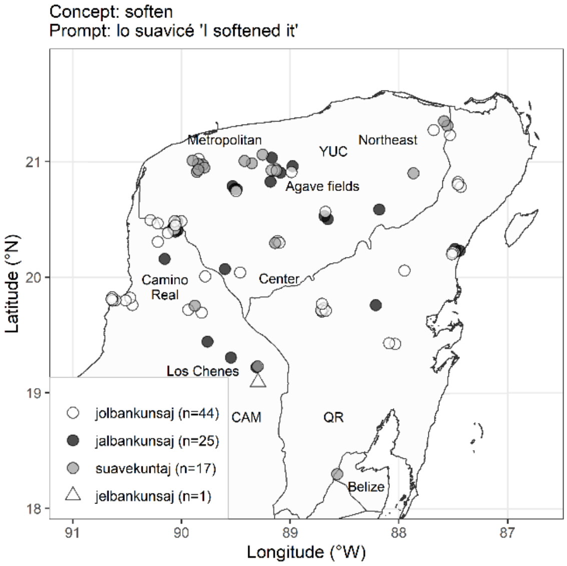

Fig 38

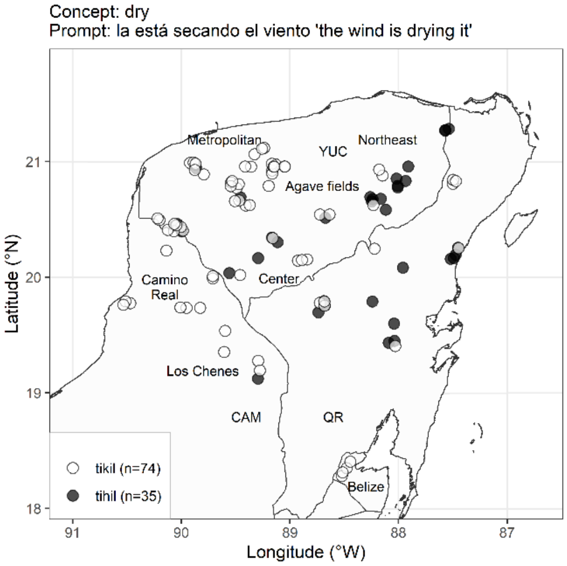

Fig 39

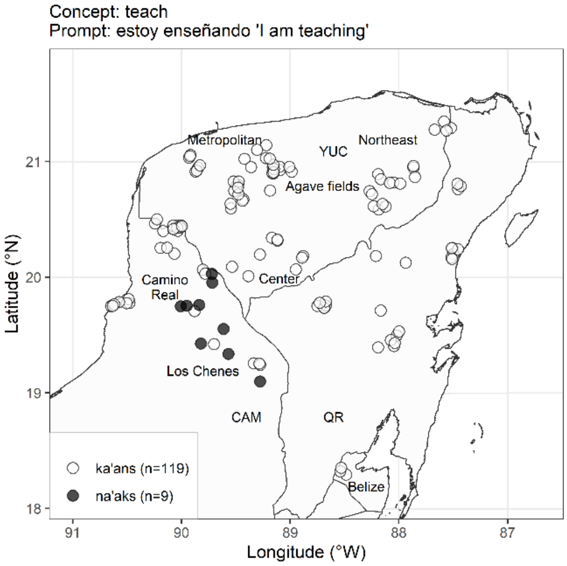

Fig 40

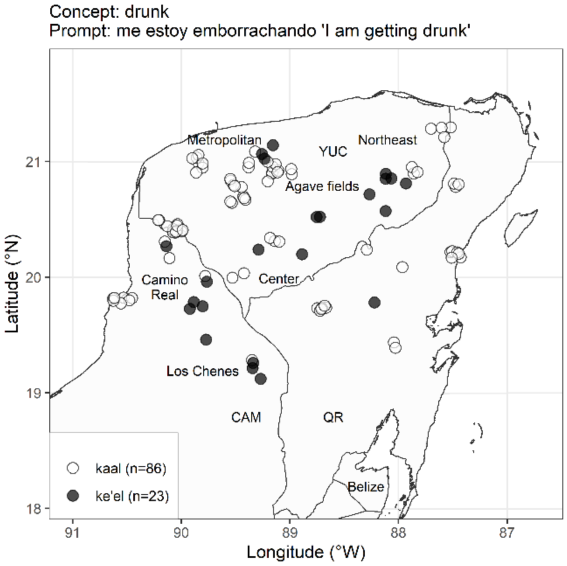

Fig 41

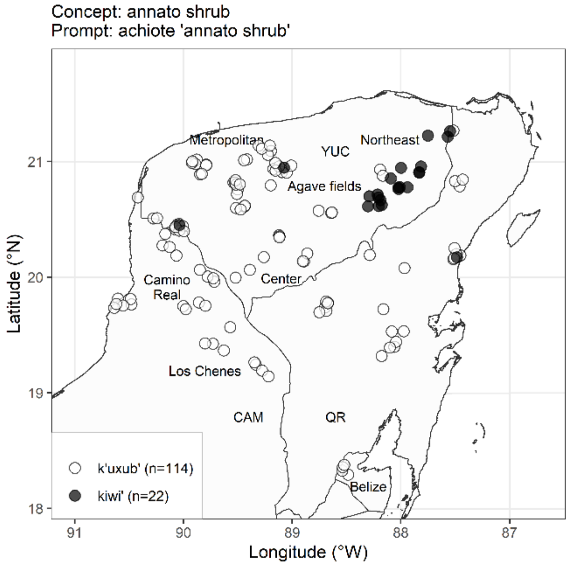

Fig 42

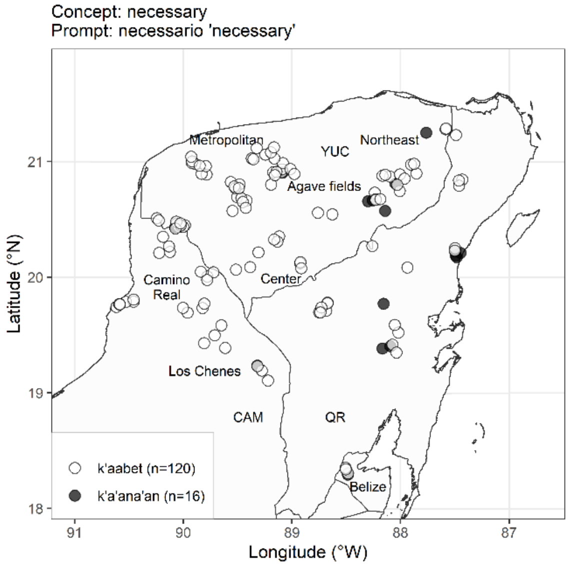

Fig 43

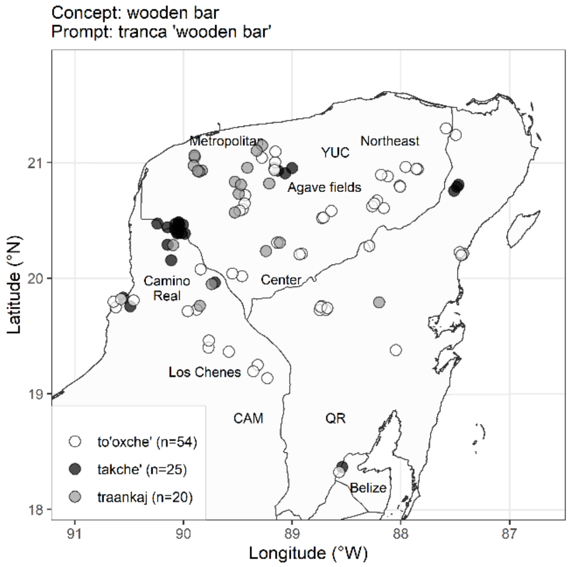

Fig 44

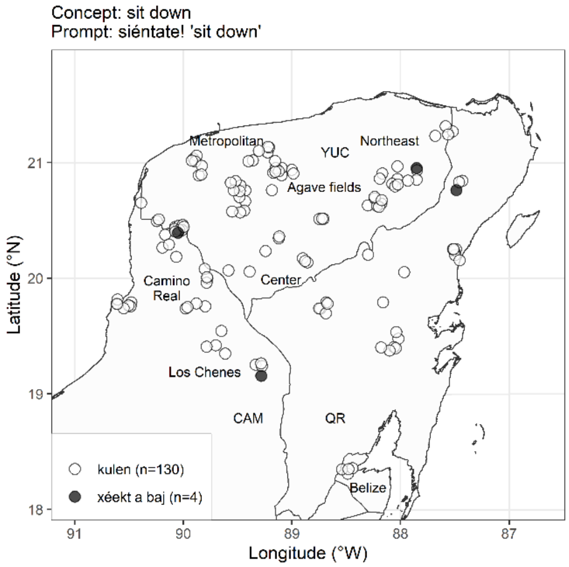

Fig 45

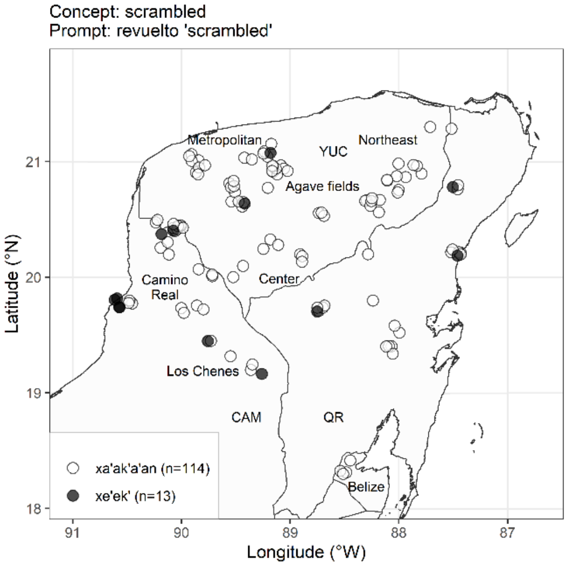

Fig 46

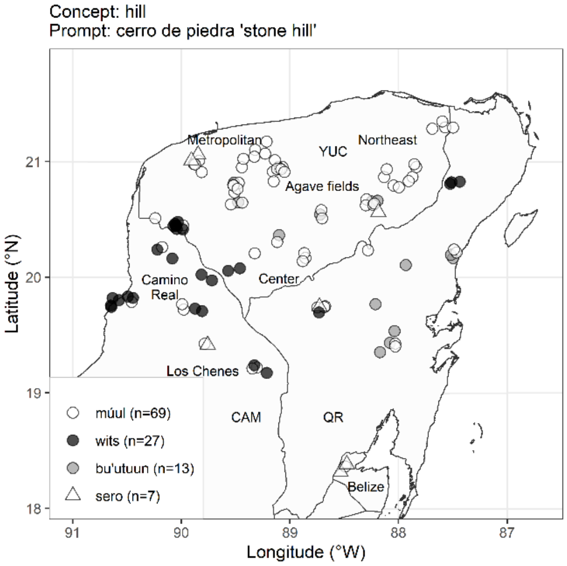

Fig 47

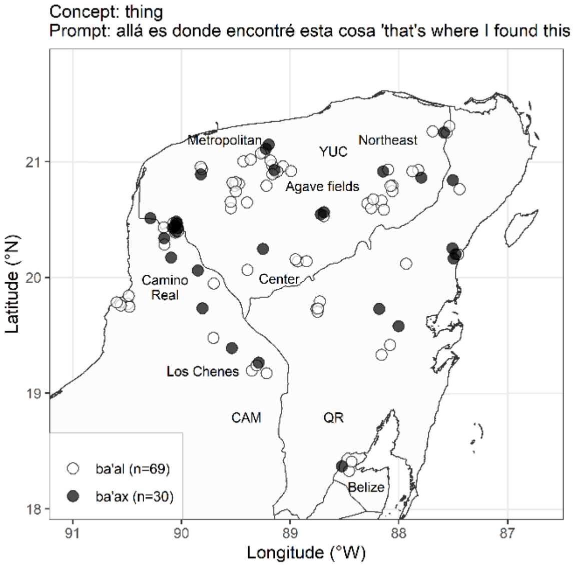

Fig 48

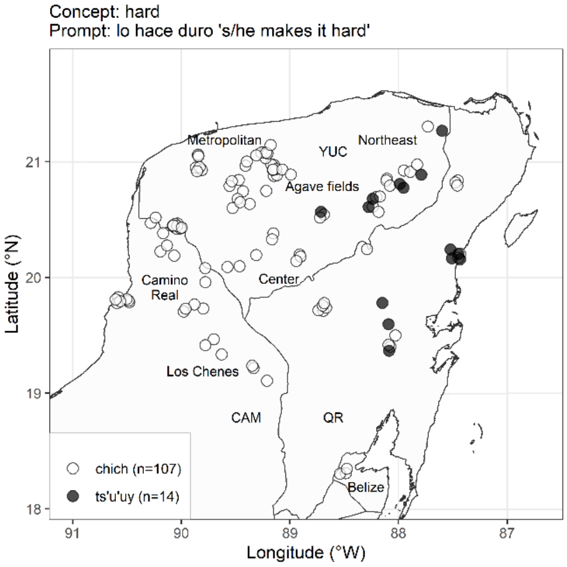

Fig 49

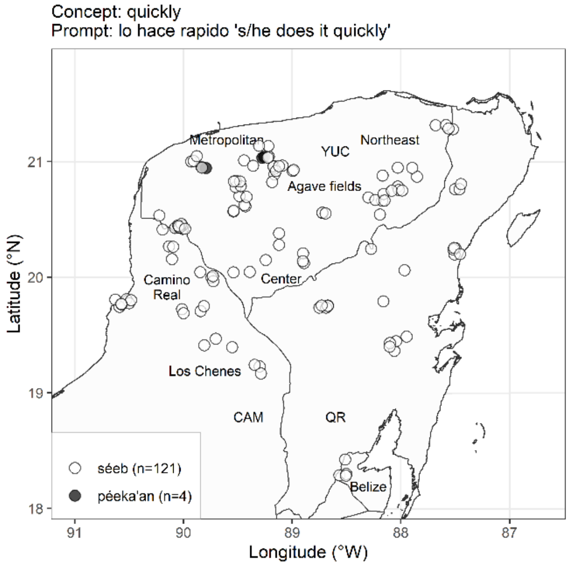

Fig 50

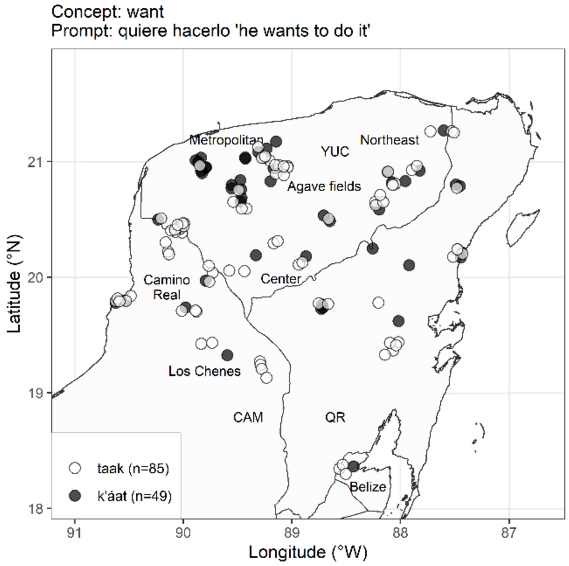

Fig 51

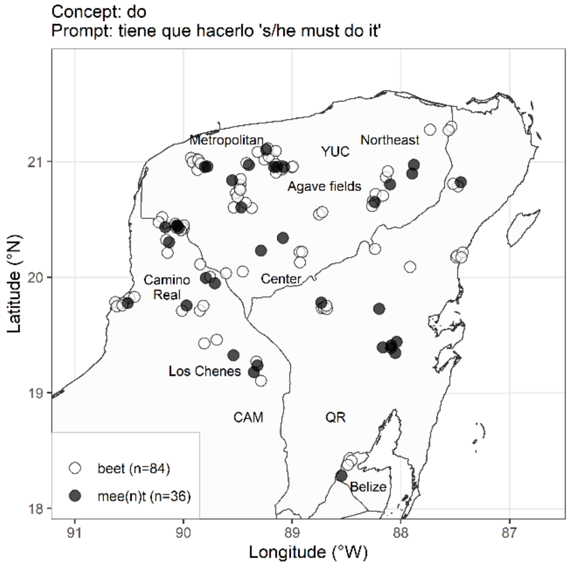

Fig 52

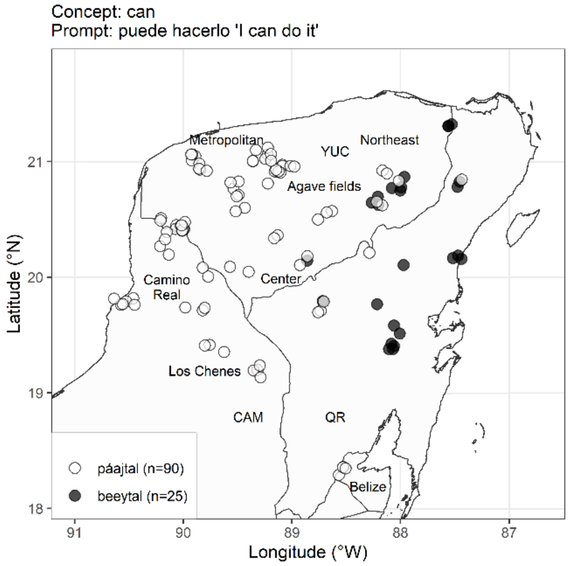

Supplement: S1 File — (PDF) [file pone.0268448.s001.pdf]
