# Supplementary material for: Sources of convergence in indigenous languages: Lexical variation in Yucatec Maya
Source: PLoS One. 2022 May 19;17(5):e0268448. doi: 10.1371/journal.pone.0268448 (PMC9119476; doi:10.1371/journal.pone.0268448)
Supplement: S2 File — (PDF) [file pone.0268448.s002.pdf]

Sources of convergence in indigenous languages: Lexical variation in Yucatec Maya

Supplementary Material 3: Data processing

Barbara Blaha Pfeiler, Stavros Skopeteas

March 18, 2022

Contents

1 Global **2**

1.1 Libraries . . . 2

1.2 Global settings . . . 3

2 Map functions **3**

2.1 General . . . 3

2.2 Map of Yucatán . . . 3

2.3 Categorical features . . . 4

2.4 Gradient features . . . 5

2.5 MDS values to RGB colours . . . 5

2.6 Features coded on a single RGB color dimension . . . 6

2.7 GAM coefficients of space . . . 6

3 Data processing functions **7**

3.1 Matrix of dummy coded variants . . . 7

3.2 Reducing dimensions of a distance matrix with MDS . . . 7

3.3 Logistic regression . . . 8

4 Data **9**

4.1 Demographic data (general) . . . 9

4.2 Speakers and Locations (in the sample) . . . 11

4.3 Linguistic data . . . 15

5 Predictions **17**

6 Spatial diffusion **18**
6.1 Descriptives . . . 18
6.2 Plot features . . . 19
6.3 Indigenous Variants . . . 19
6.4 Spanish Variants . . . 30

7 Sources **33**
7.1 References . . . 33
7.2 R and package versions . . . 33

1 Global

1.1 Libraries

io

```
library(readxl)
library(writexl)
```

data processing

```
library(reshape2)
library(varhandle)
library(dplyr)
```

graphs

```
library(ggplot2)
library(ggmap)
library(pdp)
```

geography

```
library(gmt)
library(sp)
library(geosphere)
```

statistics

```
library(MASS)
library(cluster)
library(psych)
library(scales)
library(regclass)
library(lme4)
library(mgcv)
library(itsadug)
```

1.2 Global settings

Setting active directory

```
setwd(dirname(rstudioapi::getActiveDocumentContext()$path))
```

Global graph settings

```
theme_set(theme_bw())
```

Setting the seed to a fixed value in order to control jitter values

```
myseed = 2000
```

2 Map functions

2.1 General

2.1.1 Default settings

```
jitter <- position_jitter(width = 0.05, height = 0.05)
colors = c("white","black","grey60","white","black","grey60","white","black","grey60")
shapes = c(21,21,21,24,24,24,22,22,22)
gglegend = theme(
    legend.position = c(0.002, 0.002),
    legend.justification = c("left", "bottom"),
    legend.box.just = "right",
    legend.box.background = element_rect(color="grey", size=.5)
  )
```

2.1.2 Geographical data

The .rds files are downloaded at [1] and saved in a directory "data" under the root directory.

```
  gadm.MEX <- readRDS("data/gadm36_MEX_1_sf.rds")
  gadm.BLZ <- readRDS("data/gadm36_BLZ_1_sf.rds")
  gadm <- rbind(gadm.MEX,gadm.BLZ)
```

2.2 Map of Yucatán

basic map, geographical data

```
  yuc.map.bare <- ggplot(data = gadm) +
                  geom_sf(fill = "grey99",color = "grey20",size = 0.3) +
                  coord_sf(xlim = c(-91, -86.7), ylim = c(18.1, 21.8), expand = T)+
                  scale_x_continuous(labels = abs,
                                     breaks = seq(-180,180,by=1),
```

```
                                     minor_breaks = seq(-180,0,by=.1))+
                   scale_y_continuous(labels = abs,
                                     breaks = seq(0, 100, by=1),
                                     minor_breaks = seq(0,100,by=0.2))+
                   labs(x="Longitude (°W)",y="Latitude (°N)")
```

Labeled maps:

- labels for states
- labels for reported dialectal areas
- labels for city names

```
coord <- read_excel("S2_data.xlsx", sheet = "maplabels")
  states <- droplevels(subset(coord,class=="states"))
  states2 <- droplevels(subset(coord,class=="states2"))
  areas <- droplevels(subset(coord,class=="areas"))
  cities <- droplevels(subset(coord,class=="cities" & type == "text"))
  cities2 <- droplevels(subset(coord,class=="cities2" & type == "text"))
  cities.sgm <- droplevels(subset(coord,class=="cities" & type == "segment"))
  cities.sgm2 <- droplevels(subset(coord,class=="cities2" & type == "segment"))
yuc.map.states <-  yuc.map.bare+
    annotate("text",x=states$x,y=states$y, label = states$label,size=3)
yuc.map.areas <-  yuc.map.states+
    annotate("text",x=areas$x,y=areas$y, label = areas$label,size=3)
yuc.map.cities <-  yuc.map.states+
    annotate("text",x=cities$x,y=cities$y, label = cities$label,size=3)+
    annotate("segment",x=cities.sgm$x,y=cities.sgm$y,
             xend=cities.sgm$xend, yend=cities.sgm$yend,size=.05)
```

2.3 Categorical features

The “plot.map” function visualizes categorical features (e.g., variants) on the map of Yucatán.

```
plot.map <-   function(data.subset,feature)
  {
    data.subset <- droplevels(subset(data.subset,Concept_ID == feature))
    data.subset <- droplevels(subset(data.subset,Variant != "N.V."))
    Variant.freq <- data.frame(xtabs(~Variant,data.subset))
    Variant.freq <- Variant.freq[order(-Variant.freq$Freq),]
    Variant.freq$labels <- paste0(Variant.freq$Variant," (n=", Variant.freq$Freq,")")
    Variant.sum <- sum(Variant.freq$Freq)
    colnames(Variant.freq)[2] <- "Variant.freq"
    data.subset <- merge(data.subset,Variant.freq,by="Variant",all.y = T)
    data.subset$Variant <- factor(data.subset$Variant,
                              levels=factor(Variant.freq$Variant))
    title <- concepts[concepts$Concept_ID == feature, ]$Label
    set.seed(myseed)
    geo.plot <<- yuc.map.areas+
      geom_point(data = data.subset,
                 aes(x = Longitude, y = Latitude, fill=Variant,shape=Variant),
                 size=3,
```

```
                 alpha=.7,
                 position=jitter)+
      scale_fill_manual(values=colors, labels=Variant.freq$labels)+
      scale_shape_manual(values=shapes, labels=Variant.freq$labels)+
      labs(fill = element_blank(),shape = element_blank())+
      gglegend+
      ggtitle(label=title)+
      theme(plot.title = element_text(size = 11))
    png(paste0("figs/map_",feature,".png"),width =3000, height=3000, res=600)
      print(geo.plot)
    dev.off()
}
```

2.4 Gradient features

The "plot.map.gradient" function visualizes gradient features (e.g., frequencies of variants) on the map of Yucatán.

```
plot.map.gradient <- function(data,legend,midpoint)
  {
      caption <- substitute(data)
      plot.gradient <<- yuc.map.areas +
                        geom_point(data = data,
                                    aes(x = Longitude, y = Latitude,fill=Variant.p),
                                    size=3,
                                    shape=21,
                                    alpha=.9,
                                    colour="black",
                                    position=jitter)+
                        scale_fill_gradient2(low="white",
                                    midpoint=midpoint,
                                    high="black",
                                    mid="grey99",
                                    guide = guide_colourbar(direction = "horizontal",
                                                          frame.colour="black",
                                                          barwidth = 4,
                                                          barheight = 1),
                                                          breaks=c(0,midpoint,1),
                                                          name=legend)+
                        gglegend
    png(paste0("figs/mapgr_",caption,".png", sep=""),width =3000, height=3000, res=600)
      print(plot.gradient)
    dev.off()
  }
```

2.5 MDS values to RGB colours

The "plot.mds" function visualizes mds dimensions in RGB colors on the map of Yucatán.

```
plot.mds <- function(mds,type,map) {
  name <- substitute(mds)
```

```
  set.seed(myseed)
  if(type == "location"){
    mds.ggplot <<- map+
      geom_point(data = mds, aes(x = as.numeric(Longitude),
                                 y = as.numeric(Latitude),
                                 size=log2(as.numeric(Size)),
                                 fill=I(rgb)),shape=21,alpha=.8,position=jitter)+
      theme(axis.title=element_text(size=12))+
      guides(size = "none",fill="none")+
      scale_size(range = c(1,7))
      }
  else{
    mds.ggplot <<- map+
      geom_point(data = mds, aes(x = as.numeric(Longitude),
                                 y = as.numeric(Latitude),
                                 fill=I(rgb)),size=3,shape=21,alpha=.8,position=jitter)+
      theme(axis.title=element_text(size=12))+
      guides(size = 'none',fill='none')
      }
  png(paste0("figs/mds_",name,".png"),width =3000, height=3000, res=600)
   print(mds.ggplot)
  dev.off()
}
```

2.6 Features coded on a single RGB color dimension

The “plot.mds.dim” function visualizes data coded in one of the RGB color dimensions on the map of Yucatán.

```
plot.mds.dim <- function(fit,variable,color,label,caption){
  set.seed(myseed)
  plot.dim <<- yuc.map.bare +
              geom_point(data = fit, aes(x = Longitude, y = Latitude, fill=I(variable)),
                 size=3,shape=21,alpha=.9,position=jitter)+
              ggtitle(label=label)+
              theme(legend.position = "none")
  png(paste0("figs/mds_",caption,".png"),width =2000, height=2000, res=600)
    print(plot.dim)
  dev.off()
}
```

2.7 GAM coefficients of space

The “plot.gam” function plots the GAM coefficients to the map of Yucatán.

```
plot.gam <- function(model) {
    caption = substitute(model)
    png(paste0("figs/gam_",caption,".png", sep=""),width =3000, height=3000, res=500)
        vis.gam(model,plot.type="contour", color="terrain",too.far=0.15,
                              view=c("Longitude","Latitude"),
                              main="",cex.axis=0.9, cex.lab=1.0)
```

```
        text(-89.6,21.2, label = "Metropolitan",cex=1)
        text(-90.2,19.95, labels = "Camino\nReal", xpd=TRUE,cex=1)
        text(-88,21.2, labels = "Northeast", xpd=TRUE,cex=1)
        text(-88.7,20.8, labels = "Agave fields", xpd=TRUE,cex=1)
        text(-89.8,19.2, labels = "Los Chenes", xpd=TRUE,cex=1)
        text(-89.1,20, labels = "Center", xpd=TRUE,cex=1)
        text(-88.3,18.5, labels = "Belize", xpd=TRUE,cex=1)
    dev.off()
}
```

3 Data processing functions

3.1 Matrix of dummy coded variants

The “feature2dummy” function converts a variable with a set of values to a dummy-coded matrix.

```
feature2dummy <- function(data.set,feature.set) {
    feature.dta <- data.frame(Speaker_ID = levels(factor(data.set$Speaker_ID)))
    rownames(feature.dta) <- feature.dta$Speaker_ID
    for(i in 1:length(feature.set)) {
      feature.subset <- data.set[data.set$Concept_ID == feature.set[i],]
      rownames(feature.subset) <- feature.subset$Speaker_ID
      if (length(levels(factor(feature.subset$Variant))) > 1) {
        feature.dummy <- to.dummy(feature.subset$Variant, feature.set[i])
        feature.dummy.2 <- cbind(rownames(feature.subset),feature.dummy)
        feature.dta <- cbind(feature.dta,
                             feature.dummy.2[match(feature.dta$Speaker_ID,
                                                   rownames(feature.subset)),
                                                   colnames(feature.dummy)])
      }
      feature.dta[is.na(feature.dta)] <- 0
    }
    feature.dta <- subset(feature.dta,select=-c(Speaker_ID))
    dummy.matrix <<- feature.dta
}
```

3.2 Reducing dimensions of a distance matrix with MDS

The “dist2mds” function:

- applies MDS (3-dimensional) to a distance matrix,
- rescales the mds dimensions to a [0,1] scale,
- plots the three mds dimensions to the RGB color dimensions,
- reports the r-square by comparing the input distances with the output (mds) distances; see [2].

```
dist2mds <- function(dm,coordinates){
              caption <- substitute(dm)
              mds.all <- cmdscale(as.dist(dm),eig=TRUE, k=3,x.ret = T)
              mds <- data.frame(mds.all$points)
              dist.output <- dist(mds.all$points, diag=TRUE, upper=TRUE)
```

```
                r <- cor(c(as.dist(dm)), c(dist.output))
                rsquared <<- r * r
                mds$d1 <- scales::rescale(mds$X1,to=c(0,1))
                  dist.output.1 <- dist(mds$X1, diag=TRUE, upper=TRUE)
                  r1 <- cor(c(as.dist(dm)), c(dist.output.1))
                  rsquared1 <<- r1 * r1
                mds$d2 <- scales::rescale(mds$X2,to=c(0,1))
                  dist.output.2 <- dist(mds$X2, diag=TRUE, upper=TRUE)
                  r2 <- cor(c(as.dist(dm)), c(dist.output.2))
                  rsquared2 <<- r2 * r2
                mds$d3 <- scales::rescale(mds$X3,to=c(0,1))
                  dist.output.3 <- dist(mds$X3, diag=TRUE, upper=TRUE)
                  r3 <- cor(c(as.dist(dm)), c(dist.output.3))
                  rsquared3 <<- r3 * r3
                mds$rgb <- rgb(r = mds$d1,g = mds$d2,b = 1-mds$d3)
                mds$Longitude <- coordinates[match(rownames(mds),
                                             rownames(coordinates)),"Longitude"]
                mds$Latitude <- coordinates[match(rownames(mds),
                                             rownames(coordinates)),"Latitude"]
                mds$Location <- coordinates[match(rownames(mds),
                                             rownames(coordinates)),"Location"]
                mds$Size <- coordinates[match(rownames(mds),
                                             rownames(coordinates)),"Pop.size"]
                mds <<- mds
}
```

3.3 Logistic regression

Examining the relation between Variants in the data (dependent variable) and mds-dimensions (independent variable) in logistic regressions, output in an excel table

```
mds2regression <-   function(fit,data){
    title <- substitute(fit)
    mds.corr.table <- data.frame(variable = character(),
                                 d1.b = numeric(),d1.z = numeric(),d1.p = numeric(),
                                 d2.b = numeric(),d2.z = numeric(),d2.p = numeric(),
                                 d3.b = numeric(),d3.z = numeric(),d3.p = numeric())
    data.fit <- cbind(data,fit)
    for (i in 1:(ncol(data)))
    {
      variable <- colnames(data.fit)[i]
      fit.frame <- data.frame(xtabs(~data.fit[,i],data.fit))
      min.obs = 1
      if((fit.frame[1,]$Freq > min.obs)&(fit.frame[2,]$Freq > min.obs))
        {
          variable.glm <- summary(glm(as.factor(data.fit[,i])~
                                     d1+d2+d3,family="binomial",
                                     data.fit))
            d1.b <- round(variable.glm$coef[2],digits=3)
            d2.b <- round(variable.glm$coef[3],digits=3)
            d3.b <- round(variable.glm$coef[4],digits=3)
            d1.z <- round(variable.glm$coef[10],digits=3)
```

```r
            d2.z <- round(variable.glm$coef[11],digits=3)
            d3.z <- round(variable.glm$coef[12],digits=3)
            d1.p <- round(variable.glm$coef[14],digits=3)
            d2.p <- round(variable.glm$coef[15],digits=3)
            d3.p <- round(variable.glm$coef[16],digits=3)
            mds.corr.table.row <- data.frame(variable, d1.b,d1.z,d1.p,
                                             d2.b,d2.z,d2.p,
                                             d3.b,d3.z,d3.p)
      mds.corr.table <- rbind(mds.corr.table,mds.corr.table.row)
      }
    }
    write_xlsx(mds.corr.table, paste0("mds_logregr_",title,".xlsx"))
  }
```

4 Data

The data used in this study are available in the excel file "S2.data.xlsx". Information about the contents of sheets and columns is found in the excel sheet "notes".

```r
  info <- read_excel("S2_data.xlsx", sheet = "notes")[1:3]
```

4.1 Demographic data (general)

In order to create the demographic maps, you will need to download the excel sheets with population statistics from the Mexican States of Yucatán (Campeche, CAM, Yucatán, YUC, Quintana Roo, QR) [3].

The analyses use the following information, see [4]:

- POBTOT: total population (of the location at issue)
- P3YM_HLI: persons (3-130 years old) speaking an indigenous language

Procedure:

- bind the data from all states in a single dataframe
- drop lines without longitude or without information about the indigenous speakers
- convert to GPS data
- calculate proportion of population speaking indigenous languages out of population total per location.

```r
YUC.ddta.2 <- rbind(data.frame(read_excel("data/ITER_04XLS10 CAM.xls")),
                  data.frame(read_excel("data/ITER_31XLS10 YUC.xls")),
                  data.frame(read_excel("data/ITER_23XLS10 QR.xls")))
YUC.ddta <- subset(YUC.ddta.2,select=c("NOM_MUN","NOM_LOC",
                              "LONGITUD", "LATITUD","POBTOT","P3YM_HLI"))
YUC.ddta <- droplevels(subset(YUC.ddta,(LONGITUD != "")&(P3YM_HLI != "*")))
YUC.ddta$LONGITUD.GPS <- as.numeric(char2dms(paste0(substr(YUC.ddta$LONGITUD, 1, 3),
                              "d", substr(YUC.ddta$LONGITUD, 4, 5),"\'",
                              chs = substr(YUC.ddta$LONGITUD, 6, 7),"\"W")))
YUC.ddta$LATITUD.GPS <- as.numeric(char2dms(paste0(substr(YUC.ddta$LATITUD, 1, 2),
                              "d",substr(YUC.ddta$LATITUD, 3, 4),"\'",
                              chs = substr(YUC.ddta$LATITUD, 5, 6),"\"N")))
```

```
YUC.ddta$POBTOT.NUM <- as.numeric(YUC.ddta$POBTOT)
YUC.ddta$P3YM_HLI.NUM <- as.numeric(YUC.ddta$P3YM_HLI)
YUC.ddta$PROP.MAYA <- YUC.ddta$P3YM_HLI.NUM/YUC.ddta$POBTOT.NUM
YUC.ddta <- droplevels(subset(YUC.ddta, is.na(POBTOT.NUM) == F))
```

```
demog.ggplot <- yuc.map.bare+
  theme(panel.background = element_blank(),
        panel.border = element_rect(colour = "grey60", fill=NA, size=0.5))+
  geom_point(data = YUC.ddta,
             aes(x = LONGITUD.GPS,
                 y = LATITUD.GPS,
                 fill=100*PROP.MAYA,
                 size=log(POBTOT.NUM)),
             shape=21,
             color="grey30",
             alpha=1)+
  scale_fill_gradient2('% speakers of indig. lgs',
                       low = "white",
                       high = "blue",
                       mid="lightblue",
                       na.value = "grey50",
                       midpoint=50,
                       guide = guide_legend(
                         direction = "horizontal",
                         title.position = "top",
                         label.position = "bottom",
                         label.hjust = 0.5,
                         label.vjust = 0))+
  scale_size(range = c(.1,3))+
  guides(size = 'none')+
  theme(axis.title=element_text(size=12))+
  theme(plot.title=element_text(size=20))+
  annotate("text",x=states2$x,y=states2$y, label = states2$label,size=5)+
  annotate("text",x=cities2$x,y=cities2$y, label = cities2$label,size=3.5)+
  annotate("segment",x=cities.sgm2$x,y=cities.sgm2$y,
             xend=cities.sgm2$xend, yend=cities.sgm2$yend,size=.6)+
  theme(
    legend.position = c(0.002, 0.002),
    legend.justification = c("left", "bottom"),
    legend.box.just = "right",
    legend.box.background = element_rect(color="grey", size=.5)
    )

png(paste0("figs/yuc.population.png"),width =3000, height=3000, res=600)
  demog.ggplot
  dev.off()
```

Population statistics

The data analysed below are extracted from the table “05_01B_ESTATAL.xls”, downloaded at [5].

- aggregated per age groups, starting with 10 years old, age groups of 5 years

- calculating percentages of bilingual and monolingual indigenous speakers out of the total speakers with language specification (excluding speakers that are unspecified for this feature)
- creating a plot with percentages of bilingual and monolingual indigenous speakers.

```
yuc.pop.states <- data.frame(read_excel("data/yuc.age.xlsx"))
  yuc.pop <- aggregate(. ~ age, yuc.pop.states[2:8], FUN=sum)
  yuc.pop$birth <- 2010 - as.numeric(as.character(substr(yuc.pop$age,1,2)))
  yuc.pop$total <- yuc.pop$Indlang + yuc.pop$Spanish
  yuc.pop$p.bilingual <- 100*yuc.pop$Indlang.Spanish / yuc.pop$total
  yuc.pop$p.monolingual <- 100*yuc.pop$Indlang.NoSpanish / yuc.pop$total
yuc.age <- yuc.pop[,c("birth","p.bilingual","p.monolingual")]
  yuc.age.m <- melt(yuc.age,id=c("birth"))

age.plot <- ggplot(yuc.age.m,
                    aes(x=birth, y=value,group=variable,colour=variable)) +
                    ylim(0,100)+
                    xlim(1925,2000)+
                    stat_smooth(span = 0.2,se=F,aes(y=value),size=1) +
                    scale_colour_manual(name="indigenous speakers",
                      values=c("black","grey"),labels=c("bilingual","monolingual"))+
                    labs(x="year of birth",y="% out of population total")+
                    theme(axis.text.x=element_text(angle=90, hjust=1, vjust=.5))+
                    theme(axis.title.x=element_text(size=13))+
                    theme_bw()+
                    theme(legend.position="top")
png(paste0("figs/yuc.age.png"),width =3000, height=3000, res=700)
  age.plot
  dev.off()
```

```
  grid.arrange(demog.ggplot,age.plot, ncol = 2)
```

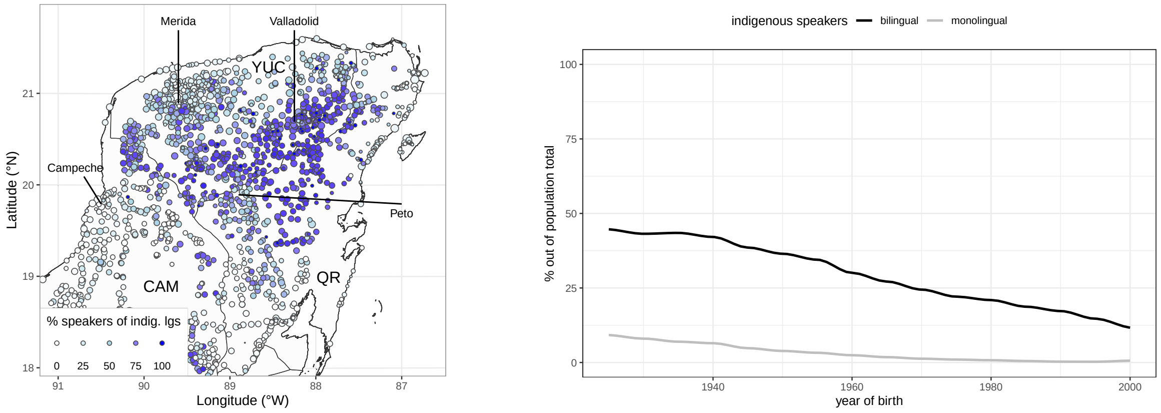

Figure 1: Proportions of indigenous speakers out of population total in the Yucatecan Peninsula (left panel), monolingual/bilingual speakers and year of birth (right panel)

4.2 Speakers and Locations (in the sample)

Dataframe speakers containing information about the speaker sample, with the following variables:

```
droplevels(subset(info,Sheet=="speakers"))
```

Sheet	Column	Content
speakers	Speaker_ID	unique identifier of speaker
speakers	Location	name of the speaker's location
speakers	Birthyear	year of birth of the speaker
speakers	Gender	male (M) or female (F)

Importing "speakers", aggregating by location

```
  speakers <- read_excel("S2_data.xlsx", sheet = "speakers",
                         col_types = c("text","text","numeric","text"))
  head(speakers)
```

Speaker_ID	Location	Birthyear	Gender
S001	San Narciso	1961	M
S002	San Pedro	1937	F
S003	Becal	1931	M
S004	Becal	1958	M
S005	Becal	1962	M
S006	Becal	1946	M

```
  speaker.location <- data.frame(xtabs(~Location,speakers))
  speaker.location <- speaker.location[order(speaker.location$Freq),]
```

The dataframe "locations" contains a list of locations, with geographic and demographic information.

```
droplevels(subset(info,Sheet=="locations"))
```

Sheet	Column	Content
locations	Location_ID	unique identifier of the sample location
locations	Location	name of location
locations	Latitude	Latitude of the location (based on googlemaps)
locations	Longitude	Longitude of the location (based on googlemaps)
locations	Pop.size	Population size of the location, based on the available census data
locations	Ind.size	Size of the indigenous population of the location

Importing locations, caluclating the proportion of indigenous population out of population total.

```
  locations <- read_excel("S2_data.xlsx", sheet = "locations",
              col_types = c("text","text","numeric","numeric","numeric","numeric"))
  locations$Ind.prop <- 100*locations$Ind.size/locations$Pop.size
  locations$Pop.size.log <- log2(as.numeric(locations$Pop.size/1000))
  head(locations)
```

Location_ID	Location	Latitude	Longitude	Pop.size	Ind.size	Ind.prop	Pop.size.log
L001	San Pedro	18.33950	-88.49521	11746	587	4.997446	3.5540976
L002	San Narciso	18.30497	-88.52152	2423	484	19.975237	1.2767944
L003	Patchacan	18.40316	-88.47325	1373	274	19.956300	0.4573316
L004	Chan Ch'en	18.44052	-88.42751	715	143	20.000000	-0.4839849
L005	Pach Huitz	19.14901	-89.24913	266	230	86.466165	-1.9105018
L006	Xmaben	19.23579	-89.31440	1228	1025	83.469055	0.2963106

Testing distributions

```
size.density <- ggplot(locations, aes(x = Pop.size)) +
                                  xlab("population size") +
                                  geom_density()
size.log.density <- ggplot(locations, aes(x = log2(Pop.size))) +
                                  xlab("logarithmized population size") +
                                  geom_density()
  grid.arrange(size.density,size.log.density, ncol = 2)
```

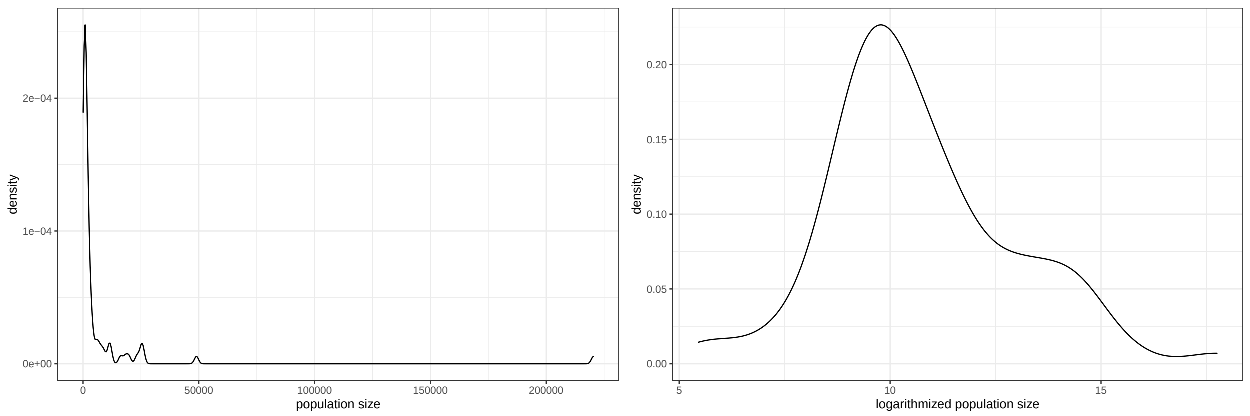

Figure 2: Density of population sizes (left panel) and of logarithmized population sizes (right panel) of the sample locations

```
png(paste0("figs/size.density.png"),width =1600, height=800, res=300)
  print(size.density)
  dev.off()
png(paste0("figs/size.log.density.png"),width =1600, height=800, res=300)
  print(size.log.density)
  dev.off()
```

Population size and indigenous proportions

```
print(corr.test(locations$Pop.size.log,locations$Ind.prop),short=FALSE)
```

```
## Call:corr.test(x = locations$Pop.size.log, y = locations$Ind.prop)
## Correlation matrix
## [1] -0.52
## Sample Size
```

```
## [1] 80
## These are the unadjusted probability values.
##   The probability values  adjusted for multiple tests are in the p.adj object.
## [1] 0
##
##  Confidence intervals based upon normal theory.  To get bootstrapped values, try cor.ci
##       raw.lower raw.r raw.upper raw.p lower.adj upper.adj
## NA-NA     -0.67 -0.52     -0.34     0     -0.67     -0.34
```

Speakers and Locations

```
locations <- merge(locations,speaker.location,by="Location",all.y = T)
rownames(locations) <- locations$Location
speaker.dta <- merge(speakers,locations, by ="Location",all.x=T)
  rownames(speaker.dta) <- speaker.dta$Speaker_ID

speakers.summaries <- c("n speakers","in n locations",
            "birthyear range","birthyear average","birthyear median",
            "n women","n men")
result <- c(nrow(speakers),length(unique(speakers$Location)),
            paste(min(speakers$Birthyear),"-",max(speakers$Birthyear)),
                  round(mean(speakers$Birthyear),1),median(speakers$Birthyear),
                  nrow(speakers[speakers$Gender == "F",]),
                  nrow(speakers[speakers$Gender == "M",]))
data.frame(speakers.summaries, result)
```

speakers.summaries	result
n speakers	157
in n locations	80
birthyear range	1906 - 1989
birthyear average	1953.7
birthyear median	1953
n women	53
n men	104

Population size and indigenous proportions

```
print(corr.test(speaker.dta$Birthyear,speaker.dta$Ind.prop),short=FALSE)
```

```
## Call:corr.test(x = speaker.dta$Birthyear, y = speaker.dta$Ind.prop)
## Correlation matrix
## [1] 0.05
## Sample Size
## [1] 157
## These are the unadjusted probability values.
##   The probability values  adjusted for multiple tests are in the p.adj object.
## [1] 0.5
##
##  Confidence intervals based upon normal theory.  To get bootstrapped values, try cor.ci
##       raw.lower raw.r raw.upper raw.p lower.adj upper.adj
## NA-NA      -0.1  0.05      0.21   0.5      -0.1      0.21
```

```r
print(corr.test(speaker.dta$Birthyear,speaker.dta$Pop.size.log),short=FALSE)
```

```
## Call:corr.test(x = speaker.dta$Birthyear, y = speaker.dta$Pop.size.log)
## Correlation matrix
## [1] 0.14
## Sample Size
## [1] 157
## These are the unadjusted probability values.
##   The probability values  adjusted for multiple tests are in the p.adj object.
## [1] 0.07
##
##  Confidence intervals based upon normal theory.  To get bootstrapped values, try cor.ci
##       raw.lower raw.r raw.upper raw.p lower.adj upper.adj
## NA-NA     -0.01  0.14      0.29  0.07     -0.01      0.29
```

4.3 Linguistic data

Variants: list of elicited variants per concept, classified for language of origin

```r
  droplevels(subset(info,Sheet=="variants"))
```

Sheet	Column	Content
variants	Concept_ID	unique identifier of the sample concepts
variants	Variant	variant occurring as lexicalization of the concept at issue
variants	Language	origin of the variant: Maya or Spanish

```r
  variants <- data.frame(read_excel("S2_data.xlsx", sheet = "variants",
                                   col_types = c("text","text","text")))
  head(variants)
```

Concept_ID	Variant	Language
C001	chúuj	Maya
C001	k'úum	Maya
C002	jool	Maya
C002	joom	Maya
C003	ki bok buuts'	Maya
C003	poom	Maya

Importing "forms", containing the elicited tokens per speaker/concept

```r
  droplevels(subset(info,Sheet=="forms"))
```

Sheet	Column	Content
forms	Speaker_ID	speaker identifier
forms	Concept_ID	concept identifier

Sheet	Column	Content
forms	Variant	variant produced for the concept at issue by the speaker at issue

Creating a unique key for tokens occurring as responses to a specific Concept.

```
forms <- data.frame(read_excel("S2_data.xlsx", sheet = "forms",
                     col_types = c("text","text","text")))
  forms$Variant_ID <- paste0(forms$Concept_ID,forms$Variant)
  variants$Variant_ID <- paste0(variants$Concept_ID,variants$Variant)
head(forms)
```

Speaker_ID	Concept_ID	Variant	Variant_ID
S001	C001	chúuj	C001chúuj
S001	C002	jool	C002jool
S001	C003	N.V.	C003N.V.
S001	C004	pool	C004pool
S001	C005	xéet’	C005xéet’
S001	C006	mixba’al	C006mixba’al

Adding basic biographical/demographic data to the “forms” dataframe.

```
  forms$Language <- variants[match(forms$Variant_ID, variants$Variant_ID),"Language"]
  speaker.dta2 <- subset(speaker.dta,select = c("Speaker_ID","Birthyear",
                                       "Location","Latitude","Longitude",
                                       "Pop.size.log","Ind.prop"))
  forms <- merge(forms,speaker.dta2,by="Speaker_ID")
```

Concepts: list of concepts and the corresponding prompts in the questionnaire

```
droplevels(subset(info,Sheet=="concepts"))
```

Sheet	Column	Content
concepts	Concept_ID	unique identifier of the sample concepts
concepts	Concept	target concept
concepts	Prompt	Spanish prompt used for eliciting the concept
concepts	English	English translation of the Spanish prompt

Importing the data frame “concepts” from the excel table and creating labels (to be used for plots of features)

```
concepts <- data.frame(read_excel("S2_data.xlsx", sheet = "concepts"))
head(concepts)
```

Concept_ID	Concept	Prompt	English
C001	squash	calabazo	squash
C002	hole	agujero	hole

Concept_ID	Concept	Prompt	English
C003	incense	incienso	incense
C004	head	su cabeza	your head
C005	piece	pedazo de piedra	piece of stone
C006	nothing	nada	nothing

```
  concepts$Label <- paste0("Concept: ",
                           paste(concepts$Concept,"Prompt: ",sep="\n"),
                           concepts$Prompt," '",
                           concepts$English,"'")
```

Data summary

```
data.summaries <- c("n variants","n forms","n concepts")
result <- c(nrow(variants),nrow(forms),nrow(concepts))
data.frame(data.summaries, result)
```

data.summaries	result
n variants	149
n forms	8164
n concepts	52

5 Predictions

Calculating distances between places: Dij is the distance between center i and center j - creating a matrix with the logarithmized distances between locations - removing the upper triangle and the diagonal of the matrix (to exclude duplicates and self distances) - reducing the distances with multidimensional scaling

```
Dij <- log2(distm(locations[,c("Longitude","Latitude")]))
  rownames(Dij) <- locations$Location
  colnames(Dij) <- locations$Location
  Dij[upper.tri(Dij,diag=T)] <- NA
  Dij.dist <- as.dist(scales::rescale(Dij,to=c(.001,.999)))
dist2mds(Dij.dist,locations); pred.by.distance <- mds
  print(paste0("R-squared, all dimensions: ",round(rsquared,digits=2)))
```

```
## [1] "R-squared, all dimensions: 0.74"
```

Population sizes

- creating a vector with logarithmized population sizes (in thousands)
- calculating population size products
- rescaling population size products to a (0,1) interval
- centering population size products around 1
- since the input to mds is a “distance” matrix, the factors of the gravity model must be reversed: ratio of distances to population sizes
- reducing dimensions of variation of the matrix with multidimensional scaling

```r
  Pop.vector <- locations$Pop.size.log
  PiPj <- Pop.vector %o% Pop.vector
  rownames(PiPj) <- locations$Location
  colnames(PiPj) <- locations$Location

  PiPj.scaled <- rescale(PiPj,to=c(.001,.999))
  PiPj.centered <- PiPj.scaled-mean(PiPj.scaled)+1
  PiPj.dist <- as.dist(PiPj.centered)
  gravity.dm <- Dij.dist/PiPj.dist

  dist2mds(gravity.dm,locations); pred.by.gravity <- mds
```

```r
  print(paste0("R-squared, all dimensions: ",round(rsquared,digits=2)))
```

```
## [1] "R-squared, all dimensions: 0.65"
```

```r
  plot.mds(pred.by.distance,"location",yuc.map.cities); mds.distance <- mds.ggplot
  plot.mds(pred.by.gravity,"location",yuc.map.cities); mds.gravity <- mds.ggplot
```

```r
grid.arrange(mds.distance,mds.gravity, ncol = 2)
```

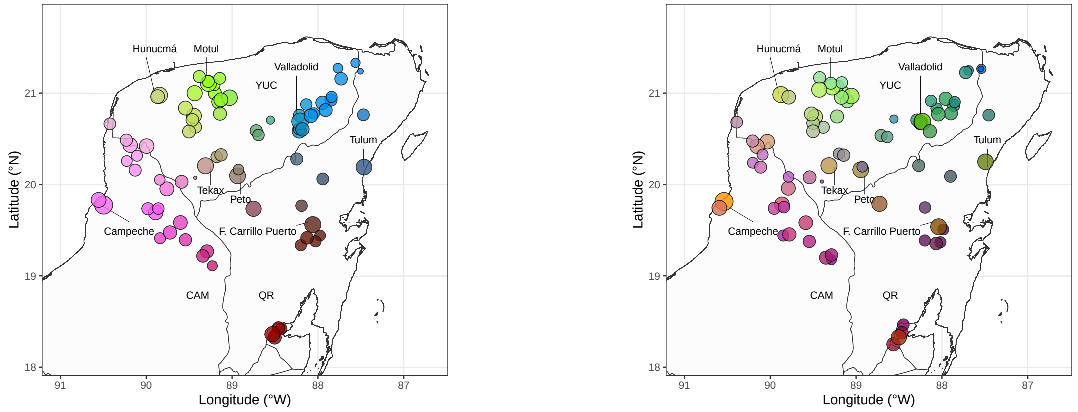

Figure 3: Predictions, wave model (left panel), gravity model (right panel)

6 Spatial diffusion

6.1 Descriptives

- creating a unique list of features occurring in the "form" data frame.
- removing non-valid values
- creating subsets with the variants of Mayan and Spanish origin
- calculating a speaker/cells matrix

```r
features <- unique(forms$Concept_ID)
valid.dta <- droplevels(subset(forms,Variant != "N.V."))
```

```
  maya.dta <- droplevels(subset(valid.dta,Language == "Maya"))
  spanish.dta <- droplevels(subset(valid.dta,Language == "Spanish"))
  speakers.xtabs <- data.frame(xtabs(~Speaker_ID,forms))
```

Data summary

```
indig.summaries <- c("n features",
                     "n speakers",
                     "n tokens",
                     "n valid",
                     "n non-valid",
                     "% valid out of n tokens",
                     "n Mayan",
                     "% Mayan out of n valid",
                     "n Spanish",
                     "% Spanish out of n valid")
indig.results <- c(length(features),
                      nrow(speakers.xtabs),
                      nrow(forms),
                      nrow(valid.dta),
                      nrow(forms)-nrow(valid.dta),
                      round((nrow(valid.dta)*100/nrow(forms)),digits=0),
                      nrow(maya.dta),
                      round((nrow(maya.dta)*100/nrow(valid.dta)),digits=0),
                      nrow(spanish.dta),
                      round((nrow(spanish.dta)*100/nrow(valid.dta)),digits=0))
data.frame(summaries = indig.summaries, results = as.character(indig.results))
```

summaries	results
n features	52
n speakers	157
n tokens	8164
n valid	6475
n non-valid	1689
% valid out of n tokens	79
n Mayan	6304
% Mayan out of n valid	97
n Spanish	171
% Spanish out of n valid	3

6.2 Plot features

```
  for(i in 1:length(features)){
    plot.map(forms,as.character(unique(forms$Concept_ID)[i]))}
```

6.3 Indigenous Variants

6.3.1 Lexical distances between speakers

Dummy coding of variants

```
  feature2dummy(maya.dta,features)
  data <- dummy.matrix
  lex.dist <- daisy(data, metric = "gower",
                        type = list(asymm = c(1:(length(data)))))
```

Reliability (Cronbach's alpha)

```
data.num <- data %>% mutate_all(funs(type.convert(as.factor(.))))
summary(psych::alpha(data.num,check.keys=TRUE))
```

```
##
## Reliability analysis
##  raw_alpha std.alpha G6(smc) average_r S/N  ase mean   sd median_r
##       0.91       0.9    0.99     0.072 9.3 0.01 0.54 0.11    0.065
```

```
  dist2mds(lex.dist,speaker.dta); lex.mds <- mds
  print(paste0("R-squared, all dimensions: ",round(rsquared,digits=2),"; ",
               "dimension 1: ",round(rsquared1,digits=2),"; ",
               "dimension 2: ",round(rsquared2,digits=2),"; ",
               "dimension 3: ",round(rsquared3,digits=2)))
```

```
## [1] "R-squared, all dimensions: 0.61; dimension 1: 0.33; dimension 2: 0.37; dimension 3: 0.03"
```

```
  plot.mds(lex.mds,"speaker",yuc.map.areas); plot.lex.mds.speaker <- mds.ggplot
```

aggregated per location

```
  lex.mds.2 <- subset(lex.mds,select=c("Location",
                                       "d1","d2","d3",
                                       "Longitude","Latitude",
                                       "Size"))
  lex.mds.aggr <- aggregate(x = lex.mds.2[,2:7],
                            by = list(lex.mds.2$Location), FUN = mean)
    rownames(lex.mds.aggr) <- lex.mds.aggr$Group.1
    lex.mds.aggr$rgb <- rgb(r = lex.mds.aggr$d1,
                            g = lex.mds.aggr$d2,
                            b = 1-lex.mds.aggr$d3)
    plot.mds(lex.mds.aggr,"location",yuc.map.cities); plot.lex.mds.location <- mds.ggplot
```

```
grid.arrange(plot.lex.mds.speaker,plot.lex.mds.location,ncol = 2)
```

```
    plot.mds.dim(lex.mds,rgb(r = mds$d1,g = 0,b = 0),
                 "red","red by dim 1 (mds)","mds.lex.d1")
    plot.mds.d1 <- plot.dim
    plot.mds.dim(lex.mds,rgb(r = 0,g = mds$d2,b = 0),
                 "green3","green by dim 2 (mds)","mds.lex.d2")
    plot.mds.d2 <- plot.dim
    plot.mds.dim(lex.mds,rgb(r = 0,g = 0,b = 1-mds$d3),
                 "blue3","blue by inverse dim 3 (mds)","mds.lex.d3")
    plot.mds.d3 <- plot.dim
    mds2regression(lex.mds,data)
```

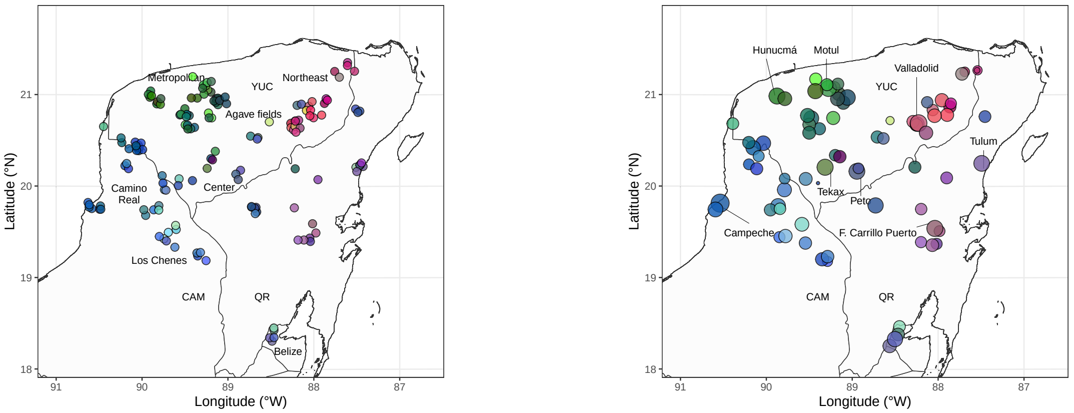

Figure 4: Estimates of multidimensional scaling mapped onto RGB colours, per speaker (left panel) and per location (right panel)

```
grid.arrange(plot.mds.d1,plot.mds.d2,plot.mds.d3,ncol = 3)
```

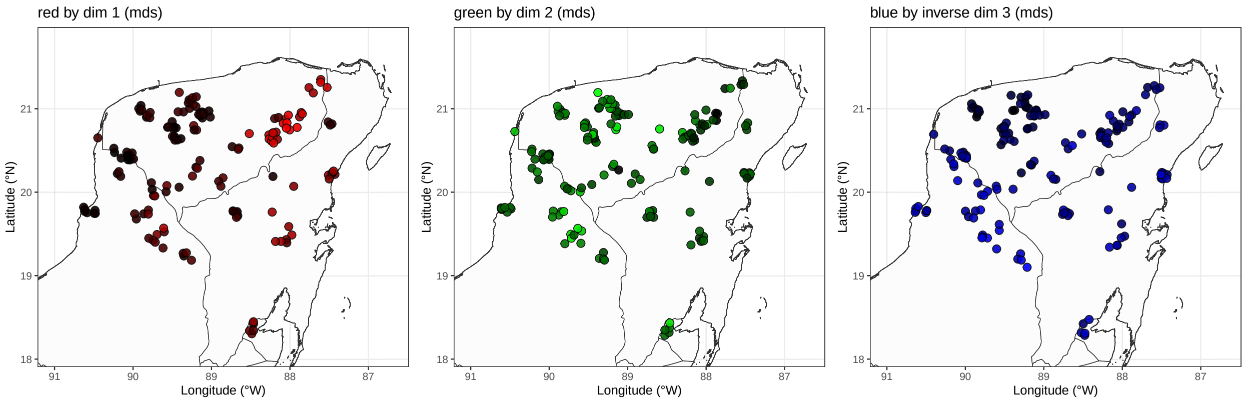

Figure 5: MDS dimensions

6.3.2 Linear model on similarity

Distance in dialect and distance in space

- create a matrix of linguistic distances
- remove self-distances
- remove duplicates

```
lex.dist.0 <- melt(as.matrix(lex.dist), varnames = c("sp1", "sp2"))
lex.dist.1 <- droplevels(subset(lex.dist.0,sp1 != sp2))
p <- t(apply(lex.dist.1[,c(1,2)],1,FUN=sort))
p <- paste(p[,1],p[,2],sep="|")
rmv <- which(duplicated(p))
sim.dta <- lex.dist.1[-c(rmv),]
sim.dta$similarity <- 1-sim.dta$value
```

Geographical distance

- calculating kilometric distances between speakers out of coordinates
- setting distance of speakers in the same location to 1km
- logarithmizing distances
- rescaling to (0,1)

```
sim.dta$sp1.Longitude <- speaker.dta[match(sim.dta$sp1,
                                     speaker.dta$Speaker_ID),"Longitude"]
sim.dta$sp1.Latitude <- speaker.dta[match(sim.dta$sp1,
                                     speaker.dta$Speaker_ID),"Latitude"]
sim.dta$sp2.Longitude <- speaker.dta[match(sim.dta$sp2,
                                     speaker.dta$Speaker_ID),"Longitude"]
sim.dta$sp2.Latitude <- speaker.dta[match(sim.dta$sp2,
                                     speaker.dta$Speaker_ID),"Latitude"]
sim.dta$Distance.km <- geodist(sim.dta$sp2.Latitude,
                                     sim.dta$sp2.Longitude,
                                     sim.dta$sp1.Latitude,
                                     sim.dta$sp1.Longitude,units="km")
sim.dta[sim.dta$Distance.km < 1, ]$Distance.km <- 1
```

```
sim.dist.lm <- lm(sim.dta$value ~ Distance.km,data=sim.dta)
sim.logdist.lm <- lm(sim.dta$value ~ log2(Distance.km),data=sim.dta)
  summary(sim.dist.lm)
```

```
##
## Call:
## lm(formula = sim.dta$value ~ Distance.km, data = sim.dta)
##
## Residuals:
##      Min       1Q   Median       3Q      Max
## -0.44664 -0.09508  0.00256  0.09495  0.42373
##
## Coefficients:
##              Estimate Std. Error t value Pr(>|t|)
## (Intercept) 5.758e-01  2.501e-03   230.3   <2e-16 ***
## Distance.km 4.267e-04  1.519e-05    28.1   <2e-16 ***
## ---
## Signif. codes:  0 '***' 0.001 '**' 0.01 '*' 0.05 '.' 0.1 ' ' 1
##
## Residual standard error: 0.1298 on 12244 degrees of freedom
## Multiple R-squared:  0.06058,    Adjusted R-squared:  0.0605
## F-statistic: 789.6 on 1 and 12244 DF,  p-value: < 2.2e-16
```

```
  summary(sim.logdist.lm)
```

```
##
## Call:
## lm(formula = sim.dta$value ~ log2(Distance.km), data = sim.dta)
##
## Residuals:
##      Min       1Q   Median       3Q      Max
```

```
## -0.41642 -0.09439  0.00253  0.09440  0.56449
##
## Coefficients:
##                     Estimate Std. Error t value Pr(>|t|)
## (Intercept)        0.4355082  0.0062259   69.95   <2e-16 ***
## log2(Distance.km) 0.0295894  0.0008943   33.09   <2e-16 ***
## ---
## Signif. codes:  0 '***' 0.001 '**' 0.01 '*' 0.05 '.' 0.1 ' ' 1
##
## Residual standard error: 0.1283 on 12244 degrees of freedom
## Multiple R-squared:  0.08207,    Adjusted R-squared:  0.082
## F-statistic:  1095 on 1 and 12244 DF,  p-value: < 2.2e-16
```

```
  AIC(sim.dist.lm)
```

```
## [1] -15256.96
```

```
  AIC(sim.logdist.lm)
```

```
## [1] -15540.36
```

```
sim.dta$Distance <- scales::rescale(log2(sim.dta$Distance.km),to=c(0,1))

#test distribution
jitter3 <- position_jitter(width = 0.01, height = 0.01)

distance.plot <- ggplot(sim.dta, aes(x = Distance.km, y = value)) +
          geom_point(size=0.01,colour="grey50",position=jitter3)+
          xlab("Distance (km)")+ylab("Dissimilarity")+
          stat_smooth(method = lm, formula = y ~ log2(x), size = 1,colour="black")
distance.plot
```

```
png(paste0("figs/distance.plot.png"),width =1600, height=800, res=300)
  print(distance.plot)
dev.off()
```

Population size

- logarithmized products of the population size of speakers' pair locations
- rescaling to (0,1)

```
sim.dta$sp1.Size <- speaker.dta[match(sim.dta$sp1,
                                  speaker.dta$Speaker_ID),"Pop.size.log"]
sim.dta$sp2.Size <- speaker.dta[match(sim.dta$sp2,
                                  speaker.dta$Speaker_ID),"Pop.size.log"]
sim.dta$Size.products <- sim.dta$sp1.Size * sim.dta$sp2.Size
sim.dta$Size <- scales::rescale(sim.dta$Size.products,to=c(0,1))
```

%Indigenous population

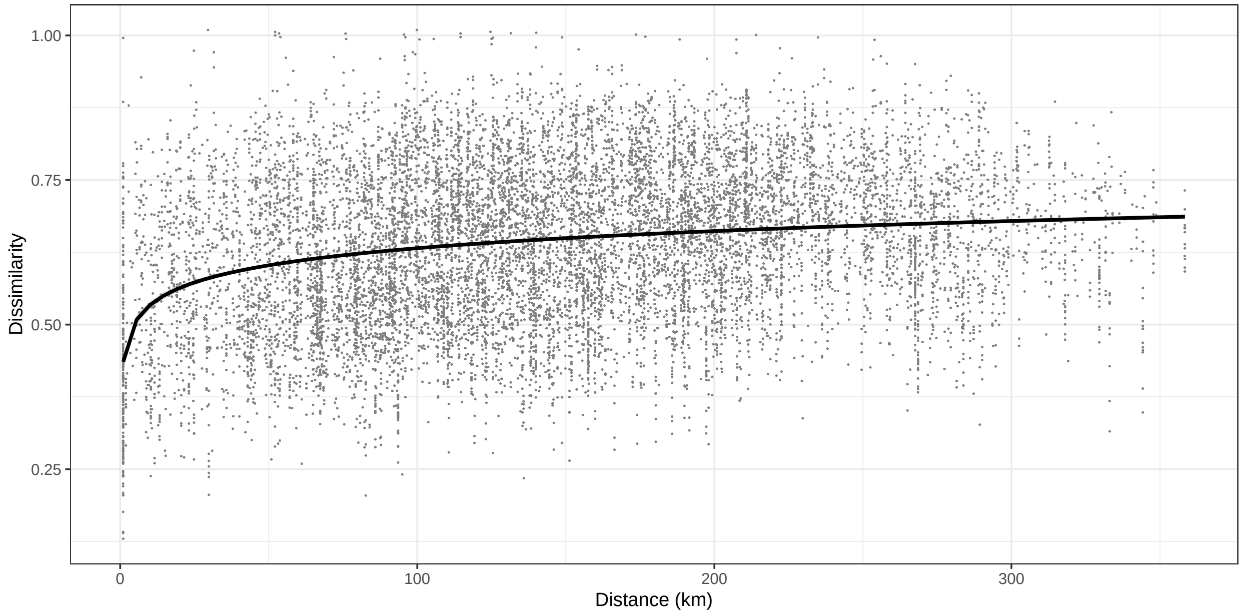

Figure 6: Dissimilarity and geographical distance (logarithmic curve)

```
sim.dta$sp1.Indig <- speaker.dta[match(sim.dta$sp1,
                                  speaker.dta$Speaker_ID),"Ind.prop"]
sim.dta$sp2.Indig <- speaker.dta[match(sim.dta$sp2,
                                  speaker.dta$Speaker_ID),"Ind.prop"]
sim.dta$Indig <- scales::rescale(sim.dta$sp1.Indig * sim.dta$sp2.Indig,to=c(0,1))
```

Time

```
sim.dta$sp1.Time <- speaker.dta[match(sim.dta$sp1,
                                  speaker.dta$Speaker_ID),"Birthyear"]
sim.dta$sp2.Time <- speaker.dta[match(sim.dta$sp2,
                                  speaker.dta$Speaker_ID),"Birthyear"]
sim.dta$Time <- scales::rescale(sim.dta$sp1.Time *
                                  sim.dta$sp2.Time,to=c(0,1))
```

Correlations between fixed effects

```
data.frame(cor_matrix(sim.dta[,c("Distance","Indig","Size","Time")]))
```

	Distance	Indig	Size	Time
Distance	1.000	-0.124	-0.071	0.052
Indig	-0.124	1.000	-0.296	0.050
Size	-0.071	-0.296	1.000	0.095
Time	0.052	0.050	0.095	1.000

Linear model on dissimilarity

- Converting dissimilarity values to similarities

- Testing the linear model

```r
sim.lm.0 <- lm(sim.dta$value ~ 1,data=sim.dta)
    data.frame(summary(sim.lm.0)$coef)
```

	Estimate	Std..Error	t.value	Pr...t..
(Intercept)	0.6379017	0.0012098	527.2837	0

Model expansion with:

- forwards update
- model comparison with analysis of variance
- model selection based on difference between AIC values

```r
sim.lm.D <- update(sim.lm.0, . ~ . +Distance)
    anova(sim.lm.0,sim.lm.D)
```

Res.Df	RSS	Df	Sum of Sq	F	Pr(>F)
12245	219.4683	NA	NA	NA	NA
12244	201.4563	1	18.01202	1094.725	0

```r
    round(AIC(sim.lm.0)-AIC(sim.lm.D),digits=1)
```

```
## [1] 1046.7
```

```r
sim.lm.DT <- update(sim.lm.D, . ~ . +Time)
    anova(sim.lm.D,sim.lm.DT)
```

Res.Df	RSS	Df	Sum of Sq	F	Pr(>F)
12244	201.4563	NA	NA	NA	NA
12243	200.4817	1	0.9745812	59.51563	0

```r
    round(AIC(sim.lm.D)-AIC(sim.lm.DT),digits=1)
```

```
## [1] 57.4
```

```r
sim.lm.DTP <- update(sim.lm.DT, . ~ . +Size)
    anova(sim.lm.DT,sim.lm.DTP)
```

Res.Df	RSS	Df	Sum of Sq	F	Pr(>F)
12243	200.4817	NA	NA	NA	NA
12242	196.8840	1	3.59769	223.6998	0

```
    round(AIC(sim.lm.DT)-AIC(sim.lm.DTP),digits=1)
```

```
## [1] 219.8
```

```
sim.lm.DTPI <- update(sim.lm.DTP, . ~ . +Indig)
    anova(sim.lm.DTP,sim.lm.DTPI)
```

Res.Df	RSS	Df	Sum of Sq	F	Pr(>F)
12242	196.8840	NA	NA	NA	NA
12241	191.2128	1	5.671292	363.0631	0

```
    round(AIC(sim.lm.DTP)-AIC(sim.lm.DTPI),digits=1)
```

```
## [1] 355.9
```

Coefficients of the winner model

```
nrow(sim.dta)
```

```
## [1] 12246
```

```
data.frame(round(summary(sim.lm.DTPI)$coefficients,digits=3))
```

	Estimate	Std..Error	t.value	Pr...t..
(Intercept)	0.425	0.010	40.483	0
Distance	0.262	0.008	34.844	0
Time	0.056	0.007	7.552	0
Size	-0.147	0.018	-8.384	0
Indig	0.086	0.005	19.054	0

6.3.3 Variants with wide geographical distribution

```
  maya.dta$Variant <- as.factor(maya.dta$Variant)
  maya.dta$Concept_ID <- as.factor(maya.dta$Concept_ID)
  variant.sums <- data.frame(maya.dta %>%
                            group_by(Concept_ID)%>%
                            dplyr::count(Variant))
  concept.sums <- data.frame(valid.dta %>%
                            dplyr::count(Concept_ID))
  variant.sums$concept.sum <- concept.sums[match(variant.sums$Concept_ID,
                                  concept.sums$Concept_ID),"n"]
  variant.sums$Variant.p <- variant.sums$n/variant.sums$concept.sum
  DI.density <- ggplot(variant.sums, aes(x = Variant.p)) +
                                  xlab("Distribution index") +
                                  geom_density()
```

```
  mean(variant.sums$Variant.p)
```

```
## [1] 0.4165293
```

```
  sd(variant.sums$Variant.p)
```

```
## [1] 0.3155451
```

```
print(DI.density)
```

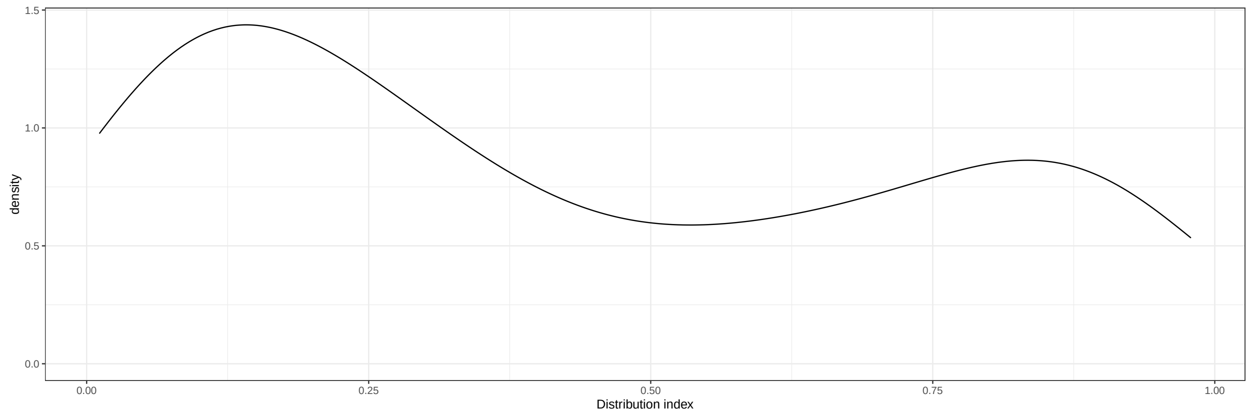

Figure 7: Density of the distribution indices of indigenous variants

```
png(paste0("figs/DI.density.png"),width =1600, height=800, res=300)
  print(DI.density)
  dev.off()
```

```
  maya.dta$Variant.p <-
                variant.sums[match(paste0(maya.dta$Concept_ID,maya.dta$Variant),
                paste0(variant.sums$Concept_ID,variant.sums$Variant)),"Variant.p"]

  diffusion.aggr <- aggregate(x = maya.dta$Variant.p,
                              by = list(maya.dta$Speaker_ID,
                                        maya.dta$Longitude,
                                        maya.dta$Latitude,
                                        maya.dta$Location,
                                        maya.dta$Birthyear,
                                        maya.dta$Pop.size.log),
                              FUN = mean)
  colnames(diffusion.aggr) <- c("Speaker","Longitude","Latitude","Location",
                                "Birthyear","Pop.size.log","Variant.p")
    plot.map.gradient(diffusion.aggr,"distr. index",.6)
    plot.gradient.indig <- plot.gradient
```

```
plot.gradient.indig
```

Rescaling: beta regression requires values in (0,1)

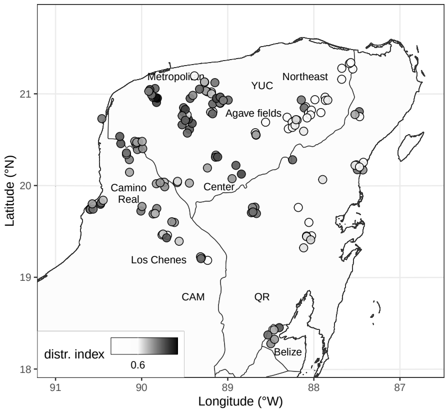

Figure 8: Occurrence of Spanish words

```r
maya.dta$Variant.p <- scales::rescale(maya.dta$Variant.p,to=c(.001,0.999))
maya.dta$Pop.size <- scales::rescale(maya.dta$Pop.size.log,to=c(0.001,0.999))
maya.dta$Time <- scales::rescale(maya.dta$Birthyear,to=c(0.001,0.999))
maya.dta$Concept <- as.factor(maya.dta$Concept_ID)
maya.dta$Ind.prop <- scales::rescale(maya.dta$Ind.prop,to=c(0.001,0.999))
nrow(maya.dta)
```

```
## [1] 6304
```

Correlation matrix

```r
data.frame(cor_matrix(maya.dta[,c("Pop.size","Ind.prop","Time")]))
```

	Pop.size	Ind.prop	Time
Pop.size	1.000	-0.674	0.135
Ind.prop	-0.674	1.000	0.063
Time	0.135	0.063	1.000

Generalized Additive Mixed-Effects Model

- calculating a gam with the smooth terms (Concept and Space)
- stepwise forwards update with model comparison

```r
gam.indigenous.0 = gam(Variant.p ~ s(Concept, bs="re")+
                          s(Longitude, Latitude, bs="tp",k=15),
                          data=maya.dta,method="ML", family="betar")
gam.indigenous.T <- update(gam.indigenous.0, . ~ . +Time)
  cm.ind.0.T <- compareML(gam.indigenous.0,gam.indigenous.T,suggest.report = T)$table
gam.indigenous.TP <- update(gam.indigenous.T, . ~ . +Pop.size)
  cm.ind.T.TP <- compareML(gam.indigenous.T,gam.indigenous.TP,suggest.report = T)$table
gam.indigenous.TI <- update(gam.indigenous.T, . ~ . +Ind.prop)
  cm.ind.T.TI <- compareML(gam.indigenous.T,gam.indigenous.TI,suggest.report = T)$table
```

```r
cm.ind.0.T
```

Model	Score	Edf	Difference	Df	p.value	Sig.
gam.indigenous.0	-3054.636	5				
gam.indigenous.T	-3057.027	6	2.391	1.000	0.029	*

```r
cm.ind.T.TP
```

Model	Score	Edf	Difference	Df	p.value	Sig.
gam.indigenous.T	-3057.027	6				
gam.indigenous.TP	-3057.793	7	0.765	1.000	0.216	

```r
cm.ind.T.TI
```

Model	Score	Edf	Difference	Df	p.value	Sig.
gam.indigenous.T	-3057.027	6				
gam.indigenous.TI	-3058.252	7	1.225	1.000	0.117	

Coefficients of the winner model

```r
  gam.indigenous <- gam.indigenous.T
    summary(gam.indigenous)
```

```
##
## Family: Beta regression(4.275)
## Link function: logit
##
## Formula:
## Variant.p ~ s(Concept, bs = "re") + s(Longitude, Latitude, bs = "tp",
##     k = 15) + Time
##
## Parametric coefficients:
##             Estimate Std. Error z value Pr(>|z|)
## (Intercept)  0.74009    0.12105   6.114 9.73e-10 ***
## Time        -0.13742    0.06281  -2.188   0.0287 *
## ---
## Signif. codes:  0 '***' 0.001 '**' 0.01 '*' 0.05 '.' 0.1 ' ' 1
##
## Approximate significance of smooth terms:
##                         edf Ref.df Chi.sq p-value
## s(Concept)            50.49   51.0 5045.7  <2e-16 ***
## s(Longitude,Latitude) 10.35   12.4  119.4  <2e-16 ***
## ---
## Signif. codes:  0 '***' 0.001 '**' 0.01 '*' 0.05 '.' 0.1 ' ' 1
##
## R-sq.(adj) =  0.434   Deviance explained = 55.9%
## -ML =  -3057  Scale est. = 1         n = 6304
```

Creating plot of the smooth term of Space

```
        plot.gam(gam.indigenous)
```

6.4 Spanish Variants

- coding languages as 0 (Maya) and 1 (Spanish)

```
  valid.dta$Variant.p <- as.numeric(as.factor(valid.dta$Language))-1
```

- reliability (Cronbach's alpha)

```
valid.lang.2 <- dcast(subset(valid.dta,select = c(Speaker_ID, Concept_ID, Language)),
                        Speaker_ID ~ Concept_ID)
valid.lang <- valid.lang.2[,c(2:ncol(valid.lang.2))]
valid.lang <- valid.lang %>%
                  mutate_all(funs(type.convert(as.integer(as.factor(.))-1)),
                             na.rm = T, as.is = FALSE)
valid.lang <- valid.lang %>%
                  mutate_all(funs(type.convert(as.logical(as.integer(as.factor(.))-1))),
                             na.rm = T, as.is = FALSE)
#summary(psych::alpha(valid.lang, na.rm=T))
  #Likely variables with missing values are  C034 C020 C021 C011 C008 C029
valid.subset <- subset(valid.lang,select=-c(C008, C011, C020, C021, C029, C034))
  summary(psych::alpha(valid.subset, na.rm=T))
```

```
##
## Reliability analysis
##  raw_alpha std.alpha G6(smc) average_r S/N   ase  mean    sd median_r
##       0.76      0.74    0.85      0.19 2.8 0.024 0.034 0.051     0.15
```

- calculating the proportions of Spanish per speaker by aggregation
- rescaling fixed factors to a (0,1) interval

```
  valid.dta$Pop.size <- scales::rescale(valid.dta$Pop.size.log,to=c(.001,.999))
  valid.dta$Time <- scales::rescale(valid.dta$Birthyear,to=c(.001,.999))
  valid.dta$Ind.prop <- scales::rescale(valid.dta$Ind.prop,to=c(.001,.999))
  valid.dta$Concept <- as.factor(valid.dta$Concept_ID)
  valid.dta$Language <- as.factor(valid.dta$Language)
  valid.dta.aggr <- aggregate(x = valid.dta[,c("Speaker_ID","Latitude",
                                             "Longitude","Variant.p")],
                                             by = list(valid.dta$Speaker_ID,
                                                       valid.dta$Longitude,
                                                       valid.dta$Latitude), FUN = mean)
  plot.map.gradient(valid.dta.aggr,"%Spanish",0)
  plot.gradient.spanish <- plot.gradient
```

```
plot.gradient.spanish
```

Correlation matrix

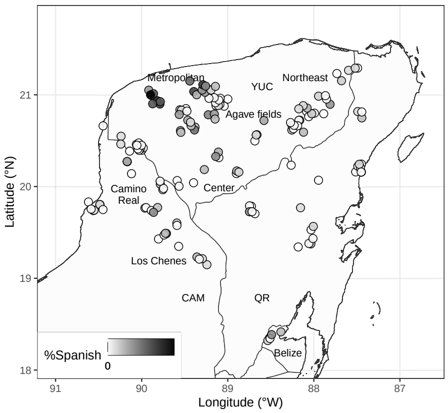

Figure 9: Occurrence of Spanish words

```
data.frame(cor_matrix(valid.dta[,c("Pop.size","Ind.prop","Time")]))
```

	Pop.size	Ind.prop	Time
Pop.size	1.000	-0.674	0.137
Ind.prop	-0.674	1.000	0.061
Time	0.137	0.061	1.000

Generalized Additive Mixed-Effects Model

- calculating a gam with the smooth terms (Concept and Space)
- stepwise forwards update with model comparison

```
gam.loans.0 = mgcv::gam((Language=="Spanish") ~
                              s(Longitude, Latitude, bs="tp",k=15)+
                              s(Concept, bs="re"),
                            data=valid.dta,
                            method="ML",
                            family=binomial)
gam.loans.T <- update(gam.loans.0, . ~ . +Time)
  cm.loans.0.T <- compareML(gam.loans.0,gam.loans.T,suggest.report = T)$table
gam.loans.TP <- update(gam.loans.T, . ~ . +Pop.size)
  cm.loans.T.TP <- compareML(gam.loans.T,gam.loans.TP,suggest.report = T)$table
gam.loans.TI <- update(gam.loans.T, . ~ . +Ind.prop)
  cm.loans.T.TI <- compareML(gam.loans.T,gam.loans.TI,suggest.report = T)$table
```

```
cm.loans.0.T
```

Model	Score	Edf	Difference	Df	p.value	Sig.
gam.loans.0	464.4198	5				
gam.loans.T	461.4400	6	2.980	1.000	0.015	*

```r
cm.loans.T.TP
```

Model	Score	Edf	Difference	Df	p.value	Sig.
gam.loans.T	461.4400	6				
gam.loans.TP	461.4393	7	0.001	1.000	0.971	

```r
cm.loans.T.TI
```

Model	Score	Edf	Difference	Df	p.value	Sig.
gam.loans.T	461.4400	6				
gam.loans.TI	461.4325	7	0.007	1.000	0.903	

Coefficients of the winner model

```r
gam.loans <- gam.loans.T
  print(summary(gam.loans))
```

```
##
## Family: binomial
## Link function: logit
##
## Formula:
## (Language == "Spanish") ~ s(Longitude, Latitude, bs = "tp", k = 15) +
##     s(Concept, bs = "re") + Time
##
## Parametric coefficients:
##             Estimate Std. Error z value Pr(>|z|)
## (Intercept)  -6.9182     0.6449 -10.728   <2e-16 ***
## Time          1.3480     0.5589   2.412   0.0159 *
## ---
## Signif. codes:  0 '***' 0.001 '**' 0.01 '*' 0.05 '.' 0.1 ' ' 1
##
## Approximate significance of smooth terms:
##                         edf Ref.df Chi.sq p-value
## s(Longitude,Latitude) 12.06  13.48  132.5  <2e-16 ***
## s(Concept)            36.44  51.00  332.1  <2e-16 ***
## ---
## Signif. codes:  0 '***' 0.001 '**' 0.01 '*' 0.05 '.' 0.1 ' ' 1
##
## R-sq.(adj) =  0.384   Deviance explained = 51.7%
## -ML = 461.44  Scale est. = 1         n = 6475
```

Creating plot of the smooth term of Space

```r
plot.gam(gam.loans)
```

7 Sources

7.1 References

[1] "Database of Global Administrative Areas" at https://gadm.org/download_country.html. (accessed 2021-12-02)

[2] Dawson, Michael R.W. 2015. "Computing The Fit Of An MDS Solution Using R", in *Cognition and Reality*, blogspot: http://cognitionandreality.blogspot.com/2015/04/computing-fit-of-mds-solution-using-r.html (accessed 2021-12-02)

[3] INEGI, Censo de Población y Vivienda 2010; at https://www.inegi.org.mx/programas/ccpv/2010/.

[4] INEGI, Censo de Población y Vivienda 2010,Infraestructura y Características Socioeconómicas de las Localidades con menos de 5 mil habitantes, Descripción de la estructura de datos; at https://www.inegi.org.mx/contenidos/programas/ccpv/2010/doc/fd_resloc_2010.pdf (accessed 2021-11-29)

[5] INEGI, Censo de Población y Vivienda 2010, Tabulados, at https://www.inegi.org.mx/programas/ccpv/2010/#Tabulados (accessed 2021-11-29).

7.2 R and package versions

```
print(sessionInfo(), RNG = F, locale = F,loadedOnly=T)
```

```
## R version 4.1.1 (2021-08-10)
## Platform: x86_64-w64-mingw32/x64 (64-bit)
## Running under: Windows 10 x64 (build 19041)
##
## Matrix products: default
##
## attached base packages:
## [1] splines   stats4    stats     graphics  grDevices utils     datasets
## [8] methods   base
##
## other attached packages:
##  [1] itsadug_2.4          plotfunctions_1.4   mgcv_1.8-36
##  [4] nlme_3.1-152         lme4_1.1-27.1       Matrix_1.3-4
##  [7] regclass_1.6         randomForest_4.6-14 rpart_4.1-15
## [10] VGAM_1.1-5           bestglm_0.37.3      leaps_3.1
## [13] scales_1.1.1         psych_2.1.9         cluster_2.1.2
## [16] MASS_7.3-54          geosphere_1.5-10    sp_1.4-5
## [19] gmt_2.0.2            pdp_0.7.0           ggmap_3.0.0
## [22] ggplot2_3.3.5        dplyr_1.0.7         varhandle_2.0.5
## [25] reshape2_1.4.4       writexl_1.4.0       readxl_1.3.1
##
## loaded via a namespace (and not attached):
##  [1] bitops_1.0-7        sf_1.0-2            httr_1.4.2
##  [4] grpreg_3.4.0        tools_4.1.1         utf8_1.2.2
##  [7] R6_2.5.0            KernSmooth_2.23-20  DBI_1.1.1
## [10] colorspace_2.0-2    withr_2.4.2         tidyselect_1.1.1
## [13] gridExtra_2.3       mnormt_2.0.2        compiler_4.1.1
## [16] cli_3.0.1           glmnet_4.1-2        labeling_0.4.2
## [19] classInt_0.4-3      proxy_0.4-26        stringr_1.4.0
```

```
## [22] digest_0.6.27      minqa_1.2.4         rmarkdown_2.10
## [25] jpeg_0.1-9         pkgconfig_2.0.3     htmltools_0.5.1.1
## [28] highr_0.9          rlang_0.4.11        rstudioapi_0.13
## [31] farver_2.1.0       shape_1.4.6         generics_0.1.0
## [34] magrittr_2.0.1     s2_1.0.6            Rcpp_1.0.7
## [37] munsell_0.5.0      fansi_0.5.0         lifecycle_1.0.0
## [40] stringi_1.7.3      yaml_2.2.1          plyr_1.8.6
## [43] grid_4.1.1         parallel_4.1.1      pls_2.7-3
## [46] crayon_1.4.1       lattice_0.20-44     tmvnsim_1.0-2
## [49] knitr_1.33         pillar_1.6.2        boot_1.3-28
## [52] rjson_0.2.20       codetools_0.2-18    wk_0.5.0
## [55] glue_1.4.2         evaluate_0.14       rpart.plot_3.1.0
## [58] png_0.1-7          vctrs_0.3.8         nloptr_1.2.2.2
## [61] foreach_1.5.1      RgoogleMaps_1.4.5.3 cellranger_1.1.0
## [64] gtable_0.3.0       purrr_0.3.4         tidyr_1.1.3
## [67] assertthat_0.2.1   xfun_0.25           e1071_1.7-8
## [70] class_7.3-19       survival_3.2-11     tibble_3.1.3
## [73] iterators_1.0.13   units_0.7-2         ellipsis_0.3.2
```
